# Multifunctional nanofibrous membranes enhance diabetic wound healing by inhibiting endothelial pyroptosis and regulating macrophage polarization

**DOI:** 10.1093/burnst/tkag005

**Published:** 2026-01-19

**Authors:** Shuang Deng, Ting Ying, Xu Zhang, Farnaz Ghorbani, Wen Luo, Chengqing Yi, Dejian Li

**Affiliations:** Department of Orthopedics, Shanghai Pudong Hospital, Fudan University Pudong Medical Center, 2800 Gongwei Road, Pudong, Shanghai 201399, China; Henan Key Laboratory of Natural Medicine Innovation and Transformation, Henan University, 85 Minglun Street, Kaifeng 475004, China; Shanghai YangZhi Rehabilitation Hospital (Shanghai Sunshine Rehabilitation Center), School of Medicine, Tongji University, 2209 Gangxing Road, Songjiang, Shanghai 200092, China; Department of Orthopedics, Shanghai Pudong Hospital, Fudan University Pudong Medical Center, 2800 Gongwei Road, Pudong, Shanghai 201399, China; Department of Translational Health Sciences, University of Bristol, Dorothy Hodgkin Building, Whtison Street, Bristol BS1 3NY, United Kingdom; Henan Key Laboratory of Natural Medicine Innovation and Transformation, Henan University, 85 Minglun Street, Kaifeng 475004, China; Department of Orthopedics, Shanghai Pudong Hospital, Fudan University Pudong Medical Center, 2800 Gongwei Road, Pudong, Shanghai 201399, China; Department of Orthopedics, Shanghai Pudong Hospital, Fudan University Pudong Medical Center, 2800 Gongwei Road, Pudong, Shanghai 201399, China

**Keywords:** Diabetic wound healing, Nanofibrous scaffold, Metal–organic frameworks, Endothelial pyroptosis, Macrophage polarization

## Abstract

**Background:**

Persistent oxidative stress and aberrant inflammatory responses are major contributors to delayed wound healing in diabetic patients. Endothelial cell pyroptosis, a form of inflammatory programmed cell death, plays a critical role in vascular dysfunction and impaired tissue regeneration in diabetic wounds. Targeting endothelial pyroptosis therefore represents a promising therapeutic strategy. This study aims to develop a multifunctional nanofibrous scaffold capable of suppressing oxidative stress-induced endothelial pyroptosis while modulating the inflammatory microenvironment to promote angiogenesis and diabetic wound repair.

**Methods:**

In this study, a pH-responsive nanoplatform based on zinc–imidazolate metal–organic frameworks (ZIF-8) was constructed for the controlled delivery of luteolin (Lut), a natural flavonoid with anti-inflammatory and antioxidant properties. The physicochemical characteristics, drug-loading efficiency, and pH-responsive release behavior of Lut@ZIF-8 nanoparticles were systematically evaluated. The effects of Lut@ZIF-8 on oxidative stress, endothelial pyroptosis, and angiogenic function were investigated *in vitro*, while therapeutic efficacy was further assessed in a diabetic mouse wound model using Lut@ZIF-8-loaded fibrous scaffolds.

**Results:**

Lut@ZIF-8 nanoparticles exhibited uniform morphology, high drug-loading efficiency, and sustained drug release under mildly acidic conditions mimicking the diabetic wound microenvironment. *In vitro*, Lut@ZIF-8 effectively suppressed reactive oxygen species accumulation and inhibited endothelial cell pyroptosis by downregulating the activation of NLRP3 inflammasome components, including caspase-1 and GSDMD, thereby preserving endothelial barrier integrity and angiogenic capacity. *In vivo*, Lut@ZIF-8-loaded scaffolds significantly reduced inflammatory cytokine expression, enhanced collagen deposition, promoted neovascularization and re-epithelialization, and ultimately accelerated wound closure in diabetic mice.

**Conclusions:**

The pH-responsive Lut@ZIF-8 nanoplatform effectively modulates oxidative stress and endothelial cell pyroptosis in diabetic wounds, thereby promoting angiogenesis and tissue regeneration. This strategy provides a promising and innovative therapeutic approach for the treatment of chronic diabetic wounds.

## Graphical Abstract

**Graphical Abstract:**
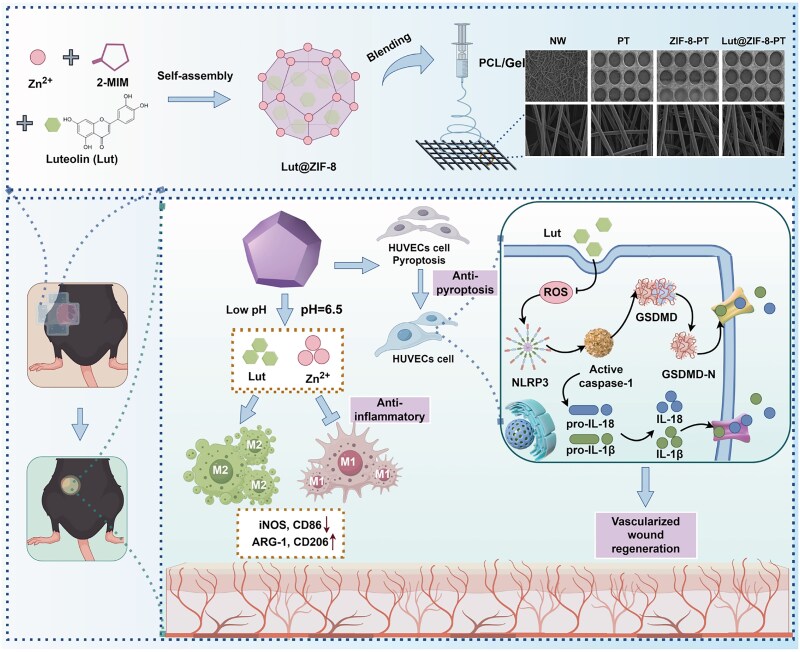

## Highlights

- Lut@ZIF-8-PT patterned nanofiber scaffolds were developed with robust physicochemical stability, biocompatibility, and sustained dual release of luteolin and Zn^2+^.
- The scaffolds promote endothelial cell migration, angiogenesis, and M2 macrophage polarization, thereby remodeling the inflammatory microenvironment.
- Endothelial pyroptosis is inhibited via suppression of the ROS–NLRP3–GSDMD axis, contributing to improved vascular regeneration.
- In a diabetic mouse wound model, the scaffolds accelerated wound closure, enhanced collagen deposition, and supported neovascularization, highlighting their potential as multifunctional therapeutic platforms for chronic wound repair.

## Background

Chronic non-healing wounds are among the most severe complications in patients with diabetes mellitus, affecting over 25% of diabetic individuals during their lifetime and often leading to infection, amputation, and significant healthcare burdens [1, 2]. Delayed healing in diabetic wounds arises from a complex interplay of cellular and molecular dysfunctions, including persistent M1 macrophage-mediated inflammation [3], endothelial cell dysfunction and pyroptosis [4], elevated oxidative stress [5], impaired angiogenesis [6], and extracellular matrix (ECM) remodeling disorders [7]. Unlike acute wounds, which progress through tightly regulated phases, diabetic wounds are frequently arrested in the inflammatory stage, failing to transition to regenerative processes [8, 9]. Central to this pathology is M1 macrophage polarization, which drives excessive release of pro-inflammatory cytokines such as TNF-α, IL-1β, and IL-6, thereby exacerbating tissue damage [10, 11]. This inflammatory milieu also triggers endothelial pyroptosis—a caspase-1-dependent programmed cell death characterized by cell swelling, membrane rupture, and release of IL-18 and IL-1β through inflammasome activation—a critical barrier to angiogenesis and wound closure in diabetic tissues [12–15]. Simultaneously, hyperglycemia-induced oxidative stress via reactive oxygen species (ROS) accumulation disrupts cellular architecture, impairs neovascularization, and amplifies inflammatory cascades [16–18]. Despite advances in wound care, current clinical interventions, including topical agents, growth factors, and negative pressure therapy, often target single pathways and fail to restore the complex microenvironment required for efficient healing [19, 20]. Thus, there is an urgent need for multifunctional therapeutic platforms capable of concurrently modulating inflammation, oxidative stress, and tissue regeneration.

In this context, natural flavonoids have emerged as promising therapeutic agents owing to their broad bioactivities. Luteolin (Lut), a plant-derived flavone, exhibits potent anti-inflammatory, antioxidant, and pro-angiogenic properties [21–23]. Mechanistically, Lut suppresses inflammasome activation, reduces ROS via the Nrf2/HO-1 pathway, and enhances endothelial tube formation through VEGF upregulation [24–27], making it an attractive candidate for diabetic wound therapy. However, poor aqueous solubility, low stability, and rapid systemic clearance severely limit its clinical application [28]. To overcome these barriers, metal–organic frameworks (MOFs)—porous crystalline materials composed of metal ions and organic ligands—have been increasingly explored as smart drug carriers [29]. Among them, ZIF-8, formed by Zn^2+^ and 2-methylimidazole, offers high surface area, pH-sensitive degradation suitable for acidic inflammatory environments, and sustained bioactive Zn^2+^ release [30–32]. Zn^2+^ itself promotes keratinocyte proliferation, modulates immune responses, and mitigates oxidative stress via SOD activation [33, 34].

Based on these insights, we developed a Lut@ZIF-8 platform via co-precipitation to improve Lut stability, enable dual controlled release of Lut and Zn^2+^, and synergistically regulate inflammation, pyroptosis, and redox balance in diabetic wounds. To further enhance tissue regenerative performance, Lut@ZIF-8 was incorporated into patterned poly (caprolactone) (PCL)/gelatin nanofibrous membranes fabricated by electrospinning. These scaffolds mimic native ECM architecture, facilitating cell adhesion, migration, and integration [35–37], and feature both randomly oriented and aligned layers to guide angiogenesis, modulate macrophage phenotypes, and promote re-epithelialization [38, 39].

This multifunctional scaffold was systematically evaluated in a full-thickness diabetic wound model. Our results demonstrated enhanced collagen deposition, revascularization, and epidermal regeneration, coupled with inhibition of M1 macrophage polarization and endothelial pyroptosis, thereby remodeling the inflammatory microenvironment, as confirmed by immunofluorescence, Western blot, flow cytometry, and transcriptomic analyses. RNA-seq further revealed downregulation of the NOD-like receptor signaling pathway, supporting the scaffold’s immunoregulatory effects.

In summary, we successfully fabricated patterned nanofiber scaffolds integrating Lut@ZIF-8 MOFs, significantly improving Lut’s drug-loading efficiency, stability, and bioavailability. Importantly, the scaffolds exhibited remarkable anti-inflammatory, antioxidant, and pro-healing effects in diabetic wounds, providing mechanistic insights into how Lut@ZIF-8-based scaffolds promote tissue regeneration.

## Methods

### Materials

PCL with average molecular weight of ~80 kDa, gelatin (gel strength ~250 Bloom), hexafluoro-2-propanol (99.9%), acetone (ACS, 98%), and Lut (99.8%) were all obtained directly from Aladdin Chemicals Reagent Co. (Shanghai, China). N, N-Dimethylformamide (DMF, 99%), dichloro-methane (DCM, 99.9%), methanol (CH3OH, 99.9%), and zinc nitrate hexahydrate (Zn(NO_3_)_2_·6H_2_O, 99%) were purchased from Fuchen Chemical Reagents Co., Ltd (Shanghai, China). Phosphate-buffered saline (PBS, pH 7.4), Span 80 (ACS, 98%), and sodium dodecyl sulfate (SDS, 99.8%) were purchased from Sigma-Aldrich (Shanghai, China). Ethanol (C_2_H_5_OH, 100%) was purchased from Richjoint Chemical Reagent Co. Ltd (Shanghai, China). All the reagents and solvents were commercially available and used as received without any further purifying treatment.

### Preparation of ZIF-8 and Lut@ZIF-8 nanoparticles

ZIF-8 was synthesized via a facile room-temperature precipitation method. Zn(NO_3_)_2_·6H_2_O (150 mg, 0.5 mmol) was dissolved in 5 ml of deionized water to obtain solution A. Separately, 2-methylimidazole (330 mg, 4 mmol) was dissolved in 10 mL of methanol to obtain solution B. Solutions A and B were mixed rapidly and allowed to react at room temperature for 1 min. The resulting white precipitate was collected by centrifugation at 10 000 rpm for 5 min, washed several times with methanol, and dried under vacuum at 60°C overnight to remove residual solvents.

To prepare Lut@ZIF-8, a co-precipitation method was employed. Zn(NO_3_)_2_·6H_2_O (150 mg, 0.5 mmol) was dissolved in 5 ml of deionized water to obtain solution C, while a mixture of 2-methylimidazole (330 mg, 4 mmol) and Lut (5.0 mg, 0.0174 mmol) was dissolved in 10 ml of methanol to obtain solution D. Solutions C and D were quickly mixed and allowed to react at room temperature for 1 min. The resulting yellow precipitate was collected by centrifugation at 10 000 rpm for 5 min, thoroughly washed with methanol, and dried in a vacuum oven at 60°C overnight.

### Fabrication of micro/nanofibrous scaffolds

PCL and gelatin (Gel) were dissolved in a mixed solvent of DCM and DMF (volume ratio DCM:DMF = 4:1) at a specific ratio to prepare a 5% (w/v) homogeneous and stable precursor solution after overnight stirring at room temperature. The solution was electrospun using a non-patterned collector to obtain randomly oriented fibrous membranes, denoted as NW. When a circular patterned collector was employed, the resulting patterned fibrous membranes were designated as PT. For the Lut@ZIF-8-PT group, 0.5% Lut@ZIF-8 was incorporated into the precursor solution before electrospinning, and the solution was collected using the circular patterned template. The electrospinning parameters were maintained at: applied voltage of 18.5 kV, constant flow rate of 0.02 ml·min^−1^, needle-to-collector distance of 15 cm, and collection time of 30 min. All experiments were conducted at room temperature (40% RH). The obtained electrospun fiber membranes were dried in a vacuum oven. All fabricated hierarchical micro/nanofibrous scaffolds were stored under vacuum for 24 hours to completely remove residual solvents prior to characterization.

### Morphological observation of ZIF-8, Lut@ZIF-8, and electrospun scaffolds

The morphology and microstructure of ZIF-8, Lut@ZIF-8, and the fabricated scaffolds were observed using a field-emission scanning electron microscope (Phenom G2 Pro, Japan). The elemental composition of ZIF-8 and Lut@ZIF-8 was analyzed using energy-dispersive X-ray spectroscopy (EDS). The particle size distribution of the nanoparticles was calculated using ImageJ software based on SEM images. The morphology and microstructure of the sample were observed using a transmission electron microscope (TEM, HT7800, acceleration voltage 200 kV). The specific method for sample preparation is as follows: Drop a uniformly dispersed nanoparticle solution onto a carbon film copper grid, and observe it after natural drying at RT.

### Characterization of ZIF-8 and Lut@ZIF-8

XRD was employed to analyze the phase composition of Lut@ZIF-8. The measurements were conducted using an X-ray diffractometer with Cu-Kα radiation (λ = 1.541874 Å) at 40 kV and 40 mA. The scanning range was set from 5° to 80° (2θ) with a scan rate of 2°/min and a step size of 0.02°. Fourier Transform Infrared Spectroscopy (FTIR) was performed using the KBr pellet method to analyze Lut, ZIF-8, and Lut@ZIF-8. The spectra were recorded in the wavenumber range of 4000–500 cm^−1^ with 32 scans for each sample. The Zetasizer Nano ZS (Malvern ZS90, UK) was used to evaluate the zeta potential of ZIF-8, Lut, and Lut@ZIF-8 according to the instruction. The surface area analyzer (ASAP 2020, Micromeritics Co., USA) was utilized to perform the N_2_ adsorption–desorption measurements at 77 K to obtain the surface area and pore size of ZIF-8 and Lut@ZIF-8. The specific surface areas were calculated by the Brunauer–Emmett–Teller (BET) method according to the adsorption isotherms of N_2_ molecules at liquid nitrogen temperature (196°C).

### *In vitro* cumulative release study

The total release amount of Lut from Lut@ZIF-8 was determined as follows. First, 1 mg of Lut@ZIF-8 powder was added to PBS buffer solutions (pH 7.4 and 6.5, respectively) containing 1 wt% Span-80 to evaluate the release characteristics of Lut. The concentration of Lut in the release medium was measured using a microplate reader (ELISA reader) within the wavelength range of 200–900 nm. For scaffold-based samples (Lut-PT and Lut@ZIF-8-PT), each sample was numbered, cut into 2.0 × 2.0 cm^2^ pieces, and weighed prior to the release experiments. Similarly, Lut@ZIF-8 powder samples were also numbered and weighed according to a defined mass. All samples (Lut@ZIF-8, Lut-PT, and Lut@ZIF-8-PT) were immersed in 10 ml of PBS containing 1 wt% Span-80 and placed in a shaker at 37°C with gentle agitation (100 rpm). At predetermined time intervals, 4 ml of the release medium was collected and replaced with an equal volume of fresh PBS–Span 80 solution (pH 7.4 or 6.5) to maintain sink conditions. The released Lut concentration in the collected samples was quantified using a microplate reader at 200–900 nm, with the corresponding fresh PBS–Span-80 solution (pH 7.4 or 6.5) serving as the blank control.

To ensure stable and reproducible drug release behavior, 1.0 wt% Span-80 was incorporated into the PBS release medium. The addition of Span 80 serves multiple purposes. First, as a nonionic surfactant, it enhances the dispersibility and wettability of hydrophobic drug molecules in the aqueous phase, preventing aggregation and enabling a uniform release profile. Second, by minimizing drug molecule aggregation, Span-80 helps maintain stable release conditions, which is essential for achieving consistent and physiologically relevant release kinetics. Notably, 1 wt% Span-80 does not alter the crystal structure or chemical integrity of ZIF-8, as confirmed by XRD analysis after the release experiments (Figure S2, see online supplementary material). Furthermore, this concentration falls within the biocompatible range reported in the literature (≤ 2 wt%), and our cell viability assays indicated no detectable cytotoxicity (Supplementary Figure 3). Therefore, 1 wt% Span 80 was selected as the release mediator to achieve an optimal balance among drug dispersibility, release reproducibility, carrier stability, and cell compatibility.

### Cell culture

Human umbilical vein endothelial cells (HUVECs) were obtained from Thermo Fisher Scientific (Cat# C0035C) and cultured in Medium 200 supplemented with low serum growth supplement (Thermo Fisher Scientific, Cat# M200500). RAW 264.7 murine macrophage cells were purchased from ATCC (Cat# TIB-71) and maintained in Dulbecco’s Modified Eagle Medium (Thermo Fisher Scientific, Cat# 11965092) supplemented with 10% fetal bovine serum (Gibco, Cat# 26140079) and 1% penicillin–streptomycin (Gibco, Cat# 15140122). All cells were incubated at 37°C in a humidified atmosphere containing 5% CO₂. All cell experiments under high-glucose conditions were conducted at 30 mM, with LPS at a concentration of 100 ng/ml.

### Cell proliferation

To evaluate the effects of different concentrations of Lut@ZIF-8-PT electrospun scaffolds on cell proliferation. Sterilized electrospun scaffolds (φ = 8 mm) were pre-soaked in PBS, and extraction media were prepared. According to the aforementioned method, which release medium prepared, the extracts were then incubated with HUVECs in 48-well plates for 24 h. Cell viability and cytotoxicity were subsequently assessed using the standard Cell Counting Kit-8 (CCK-8) assay.

To evaluate the effect of electrospun scaffolds on cell proliferation, RAW264.7 and HUVEC cells were used. Sterilized electrospun scaffolds (φ = 8 mm) were pre-soaked in PBS, and extraction media were prepared. RAW264.7 and HUVEC cells were seeded into 48-well plates at a density of 7 × 10^3^ cells per well and cultured with the scaffold extracts for 1, 3, and 5 days. Cell proliferation was assessed using a standard CCK-8 assay.

### Hemolysis assay for hemocompatibility

The hemocompatibility of ZIF-8-PT and Lut@ZIF-8-PT was evaluated via an in vitro hemolysis assay using their respective extract solutions. Fresh mouse blood was collected in Ethylenediaminetetraacetic acid (EDTA) tubes (Becton Dickinson, Cat# 367841) and centrifuged at 1000 × g for 10 min to isolate red blood cells (RBCs). The RBCs were washed three times with sterile PBS (HyClone, Cat# SH30256.01) and diluted to 2% (v/v) suspension. The sterilized electrospun scaffold were dispersed in PBS and incubated with RBC suspension at 37°C for 2 h. PBS alone served as the negative control, while 0.1% Triton X-100 (Sigma-Aldrich, Cat# T9284) served as the positive control. Following incubation, samples were centrifuged at 1000 × g for 10 min, and the absorbance of the supernatant was measured at 541 nm to quantify released hemoglobin. The hemolysis percentage was calculated as follows:

Hemolysis(%) = (A_sample_ − A_negative_)/(A_positive_ − A_negative_) × 100.

Hemolysis rate of less than 5% was considered acceptable for biomaterial hemocompatibility.

### Live/dead cell staining

Cell viability was further evaluated using the LIVE/DEAD™ Cell Imaging Kit (488/570; Thermo Fisher Scientific, Cat# R37601). After treatment, cells were stained according to the manufacturer’s instructions and imaged using a fluorescence microscope (Nikon Eclipse Ti).

### Wound healing assay

HUVECs were seeded in 6-well plates and grown to confluence. A uniform scratch was made using a sterile 200 μl pipette tip, and cells were washed to remove debris. Sterile extract solution from the electrospun Lut@ZIF-8-PT scaffold was added, and images were captured at 0 and 24 h using an inverted microscope (Olympus IX71). The wound area was quantified using ImageJ software.

### Tube formation assay

Matrigel (Corning, Cat# 356234) was added to 96-well plates and allowed to polymerize at 37°C for 30 min. HUVECs (1 × 10^4^ cells/well) were seeded onto the Matrigel-coated wells and cultured in the presence or absence of sterilized extract solution from the electrospun Lut@ZIF-8-PT scaffold. After 6 h, tube formation was observed under a microscope, and the total tube length and number of junctions were quantified using ImageJ software.

### Flow cytometry analysis

RAW264.7 cells were collected after 24 h of treatment with aseptically treated electrospun Lut@ZIF-8-PT scaffold extract and subjected to flow cytometry analysis. After washing, cells were fixed and permeabilized using the Cytofix/Cytoperm kit (BD Biosciences, product number 554714) according to the manufacturer’s instructions, and cells were subjected to Fc receptor blocking (TrueStain FcX, BioLegend) before incubation with fluorescent dye-conjugated antibodies for 30 minutes: Anti-CD86-PE(E-AB-F0994D, Elabscience, China), anti-CD206-PE (E-AB-F1135D, Elabscience, China) and anti-CD11b-FITC (E-AB-F1081C, Elabscience, China). Specifically, cells were fixed in 4% paraformaldehyde for 15 min and subsequently permeabilized in 0.2% triton-X 100 for 10 min at room temperature during the intracellular cytokines staining procedure.

### Immunofluorescence staining

Cells were fixed with 4% paraformaldehyde, permeabilized with 0.1% Triton X-100, and blocked with 5% Bovine Serum Albumin (BSA). Primary antibodies used included anti-CD206 (Thermo Fisher Scientific, Cat# PA5–46994), anti-iNOS (Proteintech, Cat# 22226–1-AP), anti-NLRP3 (Abcam, Cat# ab263899), and anti-GSDMD-N (Abcam, Cat# ab219800). After incubation with appropriate secondary antibodies, nuclei were counterstained with DAPI (Thermo Fisher Scientific, Cat# D1306), and images were captured using a confocal microscope (Zeiss LSM 900). Exposure times and image acquisition settings were kept constant across all experimental groups, and fluorescence thresholds were applied consistently during image analysis. Raw images for all groups are provided in the supplementary materials to allow independent evaluation. All experimental groups had their Best Fit threshold set to 2.00 and 0.01, with a Gain of 800 V.

### Western blot analysis

Proteins were extracted using Radio Immunoprecipitation Assay (RIPA) buffer containing protease and phosphatase inhibitors (Thermo Fisher Scientific, Cat# 78440). Equal amounts of protein were separated by SDS-PAGE and transferred onto Polyvinylidene Fluoride (PVDF) membranes. Membranes were blocked with 5% non-fat milk and incubated with primary antibodies against CD86 (Proteintech, Cat# 13395–1-AP), iNOS (Proteintech, Cat# 22226–1-AP), CD206 (Thermo Fisher Scientific, Cat# PA5–46994), Arg-1 (Cell Signaling Technology, Cat# 93668), NLRP3 (AdipoGen, Cat# AG-20B-0014), GSDMD-N (Abcam, Cat# ab209845), cleaved caspase-1 (Cell Signaling Technology, Cat# 89332), IL-1β (Cell Signaling Technology, Cat# 12703), IL-18 (Abcam, Cat# ab191860), and NF-κB p65 (Cell Signaling Technology, Cat# 8242). After incubation with HRP-conjugated secondary antibodies, bands were visualized using an ECL detection kit (Thermo Fisher Scientific, Cat# 32106).

### Quantitative real-time PCR (RT-qPCR)

Total RNA was extracted using TRIzol reagent (Thermo Fisher Scientific, Cat# 15596018), and cDNA was synthesized using the High-Capacity cDNA Reverse Transcription Kit (Thermo Fisher Scientific, Cat# 4368814). RT-qPCR was performed using PowerUp SYBR Green Master Mix (Thermo Fisher Scientific, Cat# A25742) on a QuantStudio 3 RT-qPCR System. Primer sequences for IL-6, TNF-α, IL-10, ARG-1, IL-18, HMGB1, Caspase-1, ASC, NF-κB, IL-1β, GSDMD, and NLRP3 were designed using Primer-BLAST and synthesized by Integrated DNA Technologies. The PCR reactions were performed in a 20 μl reaction volume, and the amplification conditions were as follows: 95°C for 3 minutes, followed by 40 cycles of 95°C for 10 s and 60°C for 30 s. Gene expression levels were normalized to GAPDH as the internal control, and relative expression levels were calculated using the 2^-ΔΔCt^ method. All primer sequences used for RT-qPCR are shown in Table 1.

**Table 1:** Primers used for quantitative RT-qPCR analysis

Name	Primer sequence: forward primers, reverse primers
IL-6	Forward: 5′-TACCACTTCACAAGTCGGAGGC-3′ Reverse: 5′-CTGCAAGTGCATCATCGTTGTTC-3′
TNF-α	Forward: 5′-GGTGCCTATGTCTCAGCCTCTT-3′ Reverse: 5′-GCCATAGAACTGATGAGAGGGAG-3′
IL-10	Forward: 5′-CGGGAAGACAATAACTGCACCC-3′ Reverse: 5′-CGGTTAGCAGTATGTTGTCCAGC-3′
ARG-1	Forward: 5′-CATTGGCTTGCGAGACGTAGAC-3′ Reverse: 5′-GCTGAAGGTCTCTTCCATCACC-3′
IL-18	Forward: 5′-GACAGCCTGTGTTCGAGGATATG-3′ Reverse: 5′-TGTTCTTACAGGAGAGGGTAGAC-3′
HMGB1	Forward: 5′-GAGCGTCTCAGTGGCAAAAG-3′ Reverse: 5′-CTTGCCATCCTGGTCCCTTT-3′
Caspase-1	Forward: 5′-GGCACATTTCCAGGACTGACTG-3′ Reverse: 5′-GCAAGACGTGTACGAGTGGTTG-3′
ASC	Forward: 5′-CTGCTCAGAGTACAGCCAGAAC-3′ Reverse: 5′-CTGTCCTTCAGTCAGCACACTG-3′
NF-κB	Forward: 5′-CAGATCAATGGCTACACAGG-3′ Reverse: 5′-GAGTTTCGGGTAGGAGAGGA-3′
IL-1β	Forward: 5′-TGCCACCTTTTGACAGTGATG-3′ Reverse: 5′-AAGGTCCACGGGAAAGACAC-3′
GSDMD	Forward: 5′-AGCCAGGAGGAGGAGGAGAT-3′ Reverse: 5′-TCGGAGCAGGAGGTGGAGCAG-3′

*IL* interleukin, *TNF-α* tumor necrosis factor-alpha, *ARG-1* arginase-1, *HMGB* high mobility group box 1, *NF-κB* nuclear factor kappa-light-chain-enhancer of activated B cells, *GSDMD* gasdermin D, Quantitative real-time PCR (RT-qPCR)

### ROS detection

Intracellular ROS levels were measured using the DCFDA/H2DCFDA Cellular ROS Assay Kit (Abcam, Cat# ab113851). HUVECs were incubated with 25 μM DCFDA for 45 minutes at 37°C, followed by treatment with sterilized extract solution from the electrospun Lut@ZIF-8-PT scaffold. Fluorescence intensity was measured using a microplate reader (excitation/emission: 485/535 nm) and observed under a fluorescence microscope.

### RNA sequencing and bioinformatics analysis

Total RNA was extracted from HUVECs treated with sterilized extract solution from the electrospun Lut@ZIF-8-PT scaffold using the RNeasy Mini Kit (Qiagen, Cat# 74104). HUVECs were cultured under high glucose (HG, 30 mM) conditions for RNA-seq experiments. RNA integrity was assessed using the Agilent 2100 Bioanalyzer, with RNA Integrity Number (RIN) ≥ 8.0 for all samples. Three biological replicates were used per condition. Libraries were prepared using the NEBNext Ultra II RNA Library Prep Kit (New England Biolabs, Cat# E7770) and sequenced on an Illumina NovaSeq 6000 platform, generating ~40 million paired-end reads per sample with an average alignment rate of 95% to the human reference genome (GRCh38). Differential gene expression analysis was performed using DESeq2, with a false discovery rate (FDR) threshold of 0.05 to identify significant genes. Pathway enrichment analysis was conducted using the Kyoto Encyclopedia of Genes and Genomes (KEGG) database.

### Ethical considerations

The animal experiments conducted in this study were approved by the Animal Ethics Committee of Shanghai Pudong Hospital (the animal ethics approval number: 2024-MS-IIT-D-02) in accordance with ethical principles. All surgical interventions, treatments and post-operative animal care procedures were performed in accordance with the National Institutes of Health Guide for the Care and Use of Laboratory Animals.

### In vivo diabetic wound healing model

Male C57BL/6 mice (8 weeks old) were rendered diabetic via intraperitoneal injection of streptozotocin (Sigma-Aldrich, Cat# S0130) at a dose of 50 mg/kg for 5 consecutive days. Blood glucose levels were measured from the tail vein using a glucometer (Roche Diagnostics), and mice with fasting blood glucose ≥16.7 mmol/L on two consecutive tests were considered diabetic and included in subsequent experiments. Full-thickness excisional wounds (10 mm in diameter) were created on the dorsal skin, and 10 mm × 10 mm electrospun scaffolds were applied to the wound sites. Animals were randomly assigned to experimental groups (n = 6 per group) using a random number generator. Wound closure assessment, histological scoring, and immunofluorescence quantification were performed by investigators blinded to the treatment groups. Sample size was chosen based on previous publications in similar wound healing models and was not pre-determined by formal statistical power calculation. Wound closure was monitored and photographed at designated time points.

### Histological and immunohistochemical analysis

Wound tissues were harvested, fixed in 10% formalin, embedded in paraffin, and sectioned. Hematoxylin and eosin (H&E) staining and Masson’s trichrome staining were performed using standard protocols. For immunohistochemistry, sections were incubated with anti-CD31 antibody (Abcam, Cat# ab28364) to assess neovascularization. Images were captured using a light microscope, and quantitative analysis was conducted using ImageJ.

### Statistical analysis

All experiments were performed with at least three independent biological replicates (n ≥ 3), and data are presented as mean ± standard deviation (SD). The number of replicates (n) and independent experiments are specified in the corresponding figure legends. Normality of data distribution was assessed using the Shapiro–Wilk test, and homogeneity of variance was evaluated using Levene’s test. For comparisons among more than two groups at a single time point, one-way analysis of variance (ANOVA) followed by Tukey’s honestly significant difference post hoc test, which controls the family-wise error rate for multiple pairwise comparisons, was applied. For datasets involving two factors, two-way repeated-measures ANOVA was performed, followed by Bonferroni-adjusted post hoc comparisons to correct for multiple testing across time points and treatment groups. For RNA-seq analysis, FDR correction (Benjamini–Hochberg procedure) was applied, and adjusted *P*-values <0.05 were considered significant. All statistical analyses were conducted using SPSS Statistics 25.0 (IBM, USA) and GraphPad Prism 9.0 (GraphPad Software, USA). All statistical tests were two-tailed, and differences were considered statistically significant at *P* < 0.05.

## Results

### Physicochemical characterization of ZIF-8 and Lut@ZIF-8 nanoparticles

**Figure 1:**
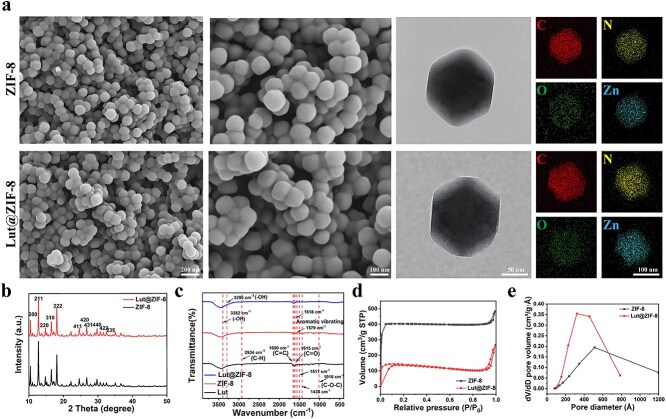
Morphological and physicochemical characterization of ZIF-8 and Lut@ZIF-8 nanoparticles. (a) SEM and TEM images of ZIF-8 and Lut@ZIF-8 showing well-defined nanostructures and EDS elemental mapping for ZIF-8 and Lut@ZIF-8. Scale bar: 50 nm, 100 nm and 200 nm. (b) XRD patterns of ZIF-8 and Lut@ZIF-8. (c) FTIR absorption spectra indicating the presence of luteolin in Lut@ZIF-8. (d-e) Pore size distribution curves of ZIF-8 and Lut@ZIF-8. *ZIF-8* zinc–imidazolate metal–organic frameworks, *Lut* luteolin, *SEM* scanning electron microscope, *TEM* transmission electron microscope, *EDS* energy dispersive spectrometer, *XRD* X-ray diffraction, *FTIR* fourier transform infrared spectroscopy

To verify the successful synthesis and Lut loading into the ZIF-8 framework, comprehensive physicochemical characterizations were conducted. SEM images revealed well-defined rhombic dodecahedral morphologies with smooth surfaces and uniform particle sizes for both ZIF-8 and Lut@ZIF-8 nanoparticles (Figure 1a). EDS elemental mapping further confirmed the homogeneous distribution of Zn, C, N, and O elements, indicating that the elemental composition of ZIF-8 was well preserved after Lut encapsulation. Particle size analysis showed average diameters of 99.36 ± 11.2 nm for ZIF-8 and 101.17 ± 11.42 nm for Lut@ZIF-8, demonstrating that Lut loading did not significantly affect nanoscale dimensions or dispersion characteristics (Figure S1, see online supplementary material).

XRD analysis revealed characteristic diffraction peaks at 2θ = 10.44°, 12.83°, and 18.10°, corresponding to the (200), (211), and (222) crystal planes of ZIF-8, confirming the preservation of the typical ZIF-8 crystalline structure after Lut incorporation (Figure 1b). In addition, XRD patterns of ZIF-8 before and after immersion in Span 80 further verified the structural stability of the framework under surfactant exposure (Figure S2, see online supplementary material). FTIR spectra provided additional evidence of successful Lut encapsulation, with characteristic absorption peaks at 1650 and 1615 cm^−1^ attributed to C=C/C=O stretching and aromatic vibrations, along with bands corresponding to aromatic and aldehyde groups, a broad O–H stretching band, and COO^−^ asymmetric/symmetric vibrations (Figure 1c).

To optimize drug loading performance, the effects of solvent composition and Lut/ZIF-8 mass ratio were systematically investigated. A methanol/water ratio of 3:1 (v/v) yielded the highest drug loading (1.27 mg/mg) and encapsulation efficiency (69.10%) among the tested conditions (Table S1, see online supplementary material). Further optimization of the Lut/ZIF-8 mass ratio revealed that balanced drug loading and encapsulation efficiency suitable for subsequent experiments was achieved under optimized conditions (Table S2, see online supplementary material).

The porous characteristics of ZIF-8 and Lut@ZIF-8 were evaluated by N₂ adsorption–desorption analysis. Both samples exhibited typical type IV isotherms with capillary condensation behavior, indicating the presence of uniform mesoporous channels (Figure 1d–e). After Lut loading, the BET surface area decreased from 1152.48 to 407.89 m^2^/g, accompanied by reductions in average pore diameter and total pore volume, reflecting partial pore occupation by Lut molecules (Table S3, see online supplementary material). These results further confirm successful incorporation of Lut within the ZIF-8 framework.

Surface chemical composition and bonding states were further analyzed by X-ray photoelectron spectroscopy (XPS), which revealed distinct changes in the Zn 2p, C 1 s, and N 1 s spectra after Lut loading, supporting the formation of coordination interactions between Lut and the ZIF-8 framework (Figure S3, see online supplementary material). Solid-state ^13C^NMR analysis provided additional molecular-level evidence of Lut encapsulation, with characteristic carbon signals from Lut clearly observed in the Lut@ZIF-8 spectrum (Figure S4, see online supplementary material).

Zeta potential measurements showed a pronounced shift from +15.9 mV for pristine ZIF-8 to −10.3 mV after Lut loading, indicating successful incorporation of negatively charged Lut molecules and altered surface charge characteristics (Figure S5, see online supplementary material). Degradation behavior was further examined under physiological (pH 7.4) and weakly acidic (pH 6.5) conditions to simulate normal and diabetic wound microenvironments. A gradual decrease in pH was observed over time, particularly under weakly acidic conditions, reflecting progressive framework degradation and release of Zn^2+^ ions and 2-methylimidazole (Figure S6, see online supplementary material). Concurrently, the zeta potential continuously decreased during degradation, reaching −24.7 mV after 14 days, indicative of Zn–N coordination bond dissociation and adsorption of anionic species (Figure S7, see online supplementary material).

Collectively, these results demonstrate that Lut@ZIF-8 nanoparticles possess well-defined morphology, preserved crystallinity, favorable porosity, and pH-responsive degradation behavior, enabling controlled Lut release in weakly acidic microenvironments and supporting their application as a bioactive nanocarrier for diabetic wound therapy.

### Morphological and elemental characterization of electrospun nanofiber scaffolds

**Figure 2:**
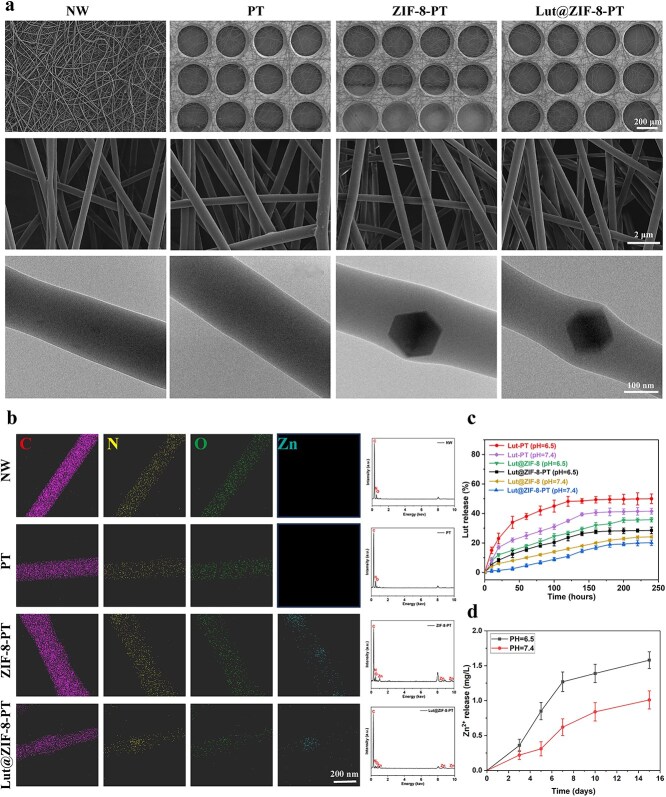
Morphological and elemental characterization of electrospun nanofiber scaffolds. (a) SEM and TEM images showing the surface morphology of NW, PT, ZIF-8-PT, and Lut@ZIF-8-PT electrospun scaffolds. The pristine PCL/gel shows a uniform nanofibrous architecture, while ZIF-8 and Lut@ZIF-8 incorporation enhances fiber roughness and complexity. Elemental mapping of Zn in Lut@ZIF-8-PT indicates uniform MOF distribution throughout the scaffold structure. Scale bar: 200 μm, 2 μm and 100 nm. (b) SEM-EDS elemental mapping images of NW, PT, ZIF-8-PT, and Lut@ZIF-8-PT scaffolds, showing the spatial distribution of C, N, O, and Zn elements. Quantitative EDS intensity analysis indicates increased levels of Zn and N in the ZIF-8-containing groups, confirming successful incorporation of MOFs into the fiber matrix. Scale bar: 200 nm. (c) Cumulative release profile of Lut from the prepared samples. (d) Quantitative determination of Zn^2+^ ion concentration released into PBS at various time points up to 15 days, reflecting the gradual release of Zn^2+^ from the Lut@ZIF-8 scaffolds. Data are expressed as mean ± SD (n = 3 independent experiments). Statistical comparisons were performed using two-way repeated-measures ANOVA (time × treatment) with Bonferroni correction. ^*^P < 0.05, ^**^P < 0.01, ^***^P < 0.001, ^****^P < 0.0001. *ZIF-8* zinc–imidazolate metal–organic frameworks, *Lut* luteolin, *SEM* scanning electron microscope, *TEM* transmission electron microscope, NW non-patterned fibrous membrane, *PT* patterned fibrous membrane, *PCL* poly(caprolactone), *MOF* metal–organic framework, *PBS* phosphate-buffered saline, *SD* standard deviation, *ANOVA* analysis of variance

The morphology of electrospun scaffolds (NW, PT, ZIF-8-PT, and Lut@ZIF-8-PT) was characterized by SEM and TEM (Figure 2a). NW scaffolds exhibited densely packed, smooth, and randomly oriented nanofibers, whereas PT, ZIF-8-PT, and Lut@ZIF-8-PT scaffolds displayed well-defined patterned micro/nano-architectures fabricated using a patterned collector. Such hierarchical topographies have been reported to facilitate cell adhesion, proliferation, and tissue ingrowth [40]. While NW and PT fibers maintained smooth surfaces and uniform diameters, incorporation of ZIF-8 slightly increased fiber diameter heterogeneity without observable nanoparticle aggregation. Notably, Lut@ZIF-8-PT scaffolds showed discernible nanoparticle features along the fiber surfaces, which are advantageous for sustained drug release. TEM observations further confirmed the absence of nanoparticles within NW and PT fibers, whereas ZIF-8-PT and Lut@ZIF-8-PT exhibited clear intrafibrillar nanoparticle distribution, indicating successful encapsulation of MOFs within the electrospun matrix.

Elemental composition and spatial distribution were analyzed by SEM-EDS mapping (Figure 2b). NW and PT scaffolds primarily consisted of C, N, and O elements, while ZIF-8-PT and Lut@ZIF-8-PT scaffolds exhibited a distinct and uniformly distributed Zn signal across the fibrous network. The homogeneous Zn distribution confirms the successful and stable incorporation of ZIF-8-based nanocarriers into the patterned scaffolds.

To overcome the instability and burst release associated with free Lut, ZIF-8 was employed as a nanocarrier owing to its high porosity, abundant coordination sites, and pH-responsive degradation behavior. As illustrated in Figure 2c, Lut@ZIF-8-PT scaffolds exhibited a controlled release profile, with ~28% of Lut released within the first 48 h, followed by sustained release over 7 days. In contrast, Lut-loaded PT scaffolds without ZIF-8 released nearly 48% of the payload within 48 h and rapidly reached equilibrium, indicating pronounced burst release. These results demonstrate that ZIF-8 effectively retards premature drug diffusion and enables prolonged delivery suitable for chronic wound treatment.

Consistent with the pH-responsive nature of ZIF-8, cumulative Zn^2+^ release from Lut@ZIF-8-PT scaffolds was significantly higher under mildly acidic conditions (pH ≈ 6.5) than under physiological conditions (pH 7.4). After 15 days, Zn^2+^ concentrations reached 1.58 mg/L in acidic media compared with 1.01 mg/L at neutral pH (Figure 2d), remaining within biologically safe and therapeutically relevant ranges.

Finally, scaffold degradation behavior was evaluated to ensure compatibility with tissue regeneration dynamics. As shown in Figure S8, PT, ZIF-8-P,T, and Lut@ZIF-8-PT scaffolds exhibited <30% mass loss within the first 7 days, primarily attributed to gelatin hydrolysis. After 14 days, the degradation rates of all groups became comparable, indicating near-complete gelatin degradation while maintaining overall scaffold integrity during the early healing phase.

### In vitro biocompatibility and angiogenic potential of the Scaffoldg

To evaluate the biointerface performance and biosafety of Lut@ZIF-8-PT scaffolds, preliminary cytocompatibility screening was first performed. Because Span 80 was used during scaffold fabrication, its potential cytotoxicity was assessed independently. CCK-8 assays demonstrated that Span 80 at the applied concentrations exerted no significant adverse effects on HUVEC viability over 1, 3, and 5 days, confirming its suitability for subsequent scaffold preparation (Figure S9, see online supplementary material).

Next, the optimal Lut@ZIF-8 loading concentration was determined using HUVEC metabolic activity assays. As shown in Figure S10, low loading (0.1%) showed no cytotoxicity but limited bioactivity, whereas moderate loading (0.5%) significantly enhanced cell metabolic activity. In contrast, high loading (1%) resulted in reduced cell viability. Live/Dead staining further confirmed that 0.5% Lut@ZIF-8-PT supported robust cell survival with minimal dead cells, while 1% loading induced cytotoxicity (Figure S11, see online supplementary material). Accordingly, 0.5% Lut@ZIF-8 was selected for all subsequent experiments.

Hemocompatibility is a critical requirement for wound dressings. In vitro hemolysis assays demonstrated that PT, ZIF-8-PT, and Lut@ZIF-8-PT scaffolds induced negligible red blood cell lysis, with hemolysis ratios well below the accepted safety threshold, indicating excellent blood compatibility (Figure S12, see online supplementary material).

**Figure 3:**
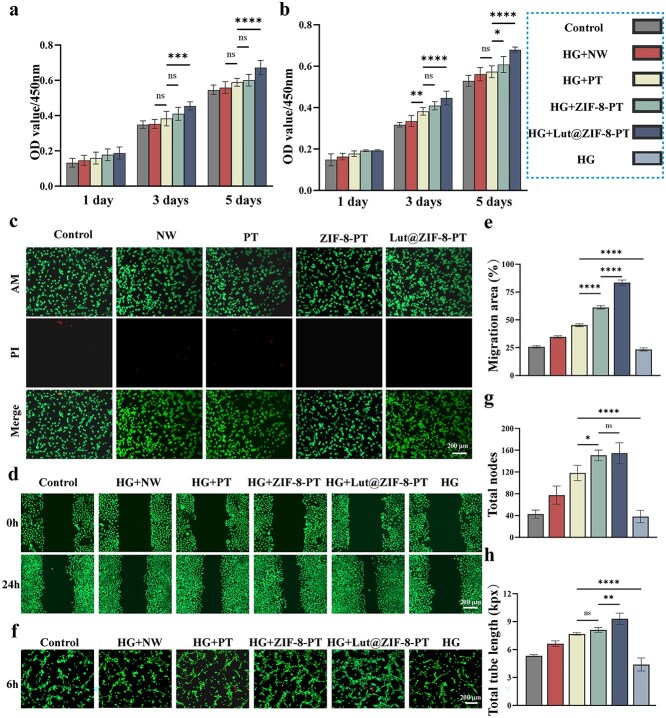
Evaluation of in vitro biocompatibility and angiogenic potential of the scaffolds. (a) CCK-8 assay of HUVEC viability at days 1, 3, and 5. (b) CCK-8 assay of RAW264.7 viability at the same time points. (c) Live/dead staining of HUVECs cultured on NW, PT, ZIF-8-PT, and Lut@ZIF-8-PT for 24 h. Scale bar: 200 μm. (d) Representative scratch wound images of HUVECs at 0 and 24 h. Scale bar: 200 μm. (e) Quantification of wound closure rate. (f) Tube formation of HUVECs after treatment with scaffold extracts. Scale bar: 200 μm. (g) Quantification of the total number of capillary-like structures. (h) Quantification of total tube length formed by HUVECs. Control: Control group; HG: High-glucose model group(30 mM); HG + NW: High-glucose+NW; HG + PT: High-glucose+PT; HG + ZIF-8-PT: High-glucose +ZIF-8-PT; HG + Lut@ZIF-8-PT:High-glucose+Lut@ZIF-8-PT, data represent mean ± SD (n = 3 independent experiments). Statistical significance was determined using one-way ANOVA or two-way repeated-measures ANOVA (time × treatment), followed by Tukey’s or Bonferroni post hoc tests as appropriate. ^*^P < 0.05, ^**^P < 0.01, ^***^P < 0.001, ^****^P < 0.0001. *ZIF-8* zinc–imidazolate metal–organic frameworks, *Lut* luteolin, *OD* optical density, *HG* high glucose, *NW* non-patterned fibrous membrane, *PT* patterned fibrous membrane, *HUVEC* human umbilical vein endothelial cells, *ANOVA* analysis of variance, *SD* standard deviation

The overall biocompatibility of the scaffolds was further evaluated using HUVECs and RAW264.7 macrophages. CCK-8 assays revealed that all scaffold extracts exhibited minimal cytotoxicity over 1, 3, and 5 days. Notably, Lut@ZIF-8-PT slightly promoted HUVEC proliferation compared with other groups, indicating favorable endothelial compatibility (Figure 3a). RAW264.7 macrophages showed comparable viability across all groups, confirming low cytotoxicity toward immune cells (Figure 3b). Live/Dead staining corroborated these results, with predominantly viable cells observed on Lut@ZIF-8-PT scaffolds (Figure 3c).

Given the critical role of endothelial migration and angiogenesis in wound healing, the pro-angiogenic potential of the scaffolds was further assessed. Scratch wound assays demonstrated that HUVECs treated with Lut@ZIF-8-PT extracts exhibited significantly accelerated wound closure at 24 h compared with other groups (Figure 3d and e). Consistently, Matrigel tube formation assays showed that Lut@ZIF-8-PT markedly enhanced capillary-like network formation, as evidenced by increased tube numbers and total tube length (Figure 3f–h). These findings indicate that Lut@ZIF-8-PT scaffolds effectively promote endothelial migration and angiogenesis, likely attributable to the sustained release of Lut and Zn^2+^ ions.

### Modulation of macrophage polarization toward an anti-inflammatory phenotype by Lut@ZIF-8-PT scaffold

To assess the immunomodulatory effects of Lut@ZIF-8-PT on macrophage polarization, RAW264.7 cells were treated with extracts from different scaffold formulations and analyzed using flow cytometry, immunofluorescence, RT-qPCR, and western blotting. Fluorescent ROS staining revealed substantial intracellular ROS accumulation in the LPS and NW groups, whereas Lut@ZIF-8-PT treatment significantly reduced ROS levels in RAW264.7 cells (Figure S13, see online supplementary material).

**Figure 4:**
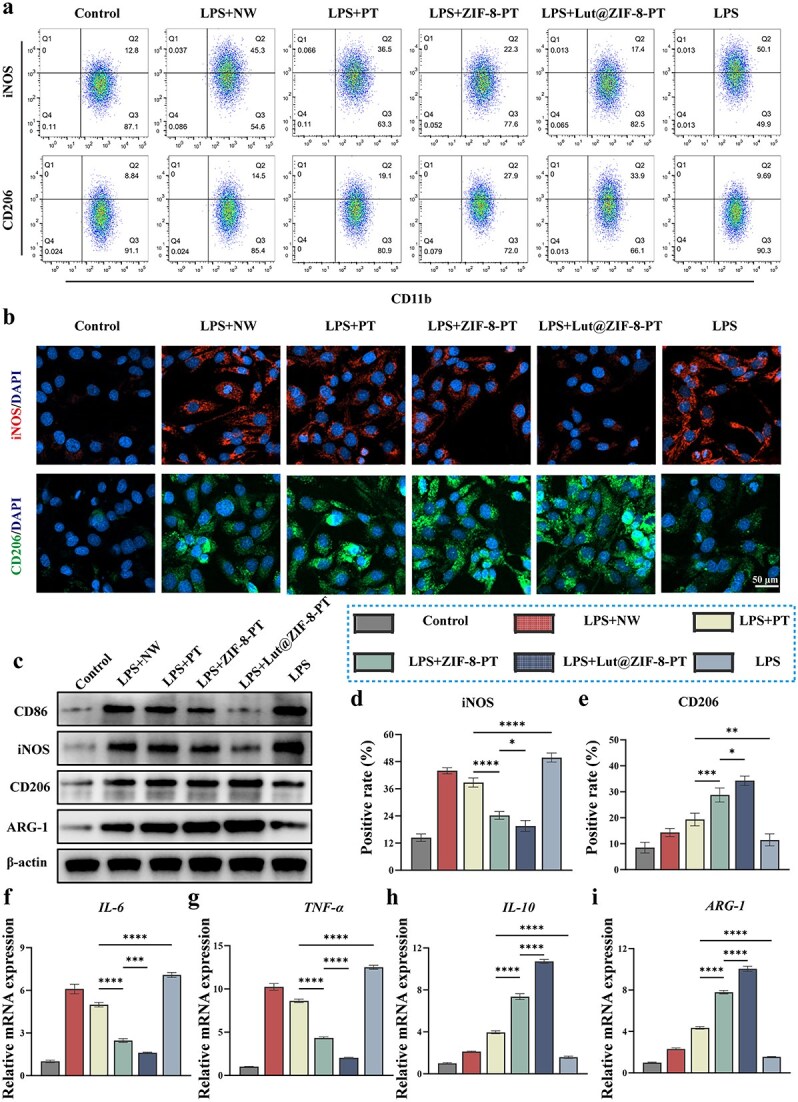
In vitro immunomodulatory effects of the scaffolds on macrophage polarization. (a) Flow cytometry analysis of RAW264.7 cells stained with CD11b/CD206 and CD11b/iNOS antibodies. (b) Immunofluorescence staining for CD206 (green) and iNOS (red) expression in macrophages. Scale bar: 50 μm. (c) Western blot analysis of polarization markers (CD86, iNOS, CD206, and ARG-1). (d-e) Quantification of CD206 and iNOS fluorescence intensity. (f-i) RT-qPCR analysis of gene expression levels of IL-6, TNF-α, IL-10, and ARG-1. Control: Control group; LPS: LPS treated group(200 ng/ml); LPS + NW: LPS treated+NW; LPS + PT: LPS treated+PT; LPS + ZIF-8-PT: LPS treated+ZIF-8-PT; LPS + Lut@ZIF-8-PT: LPS treated+Lut@ZIF-8-PT, data are expressed as mean ± SD (n = 3 independent experiments). One-way ANOVA with Tukey’s post hoc test was used to determine statistical significance. ^*^P < 0.05, ^**^P < 0.01, ^***^P < 0.001, ^****^P < 0.0001. *ZIF-8* zinc–imidazolate metal–organic frameworks, *Lut* luteolin, *LPS* lipopolysaccharide, *iNOS* inducible nitric oxide synthase, *DAPI* 4′,6-diamidino-2-phenylindole, *ARG-1* arginase-1, *IL* interleukin, *SD* standard deviation, *TNF-α* tumor necrosis factor-alpha, *RT-qPCR* reverse transcription quantitative polymerase chain reaction

Flow cytometric analysis, employing dual staining for CD11b/CD206 and CD11b/iNOS to identify M2 and M1 populations, respectively, demonstrated a marked increase in CD206^+^ (M2) and a decrease in iNOS^+^ (M1) macrophages upon Lut@ZIF-8-PT treatment compared to other groups (Figure 4a), indicating a shift toward an anti-inflammatory phenotype. Immunofluorescence staining corroborated these findings, with enhanced CD206 (green) and reduced iNOS (red) signal intensity in the Lut@ZIF-8-PT group (Figure 4b). Quantitative analysis confirmed significant promotion of M2 polarization and suppression of M1 activation (Figure 4c and d).

Western blot analysis further validated these observations. Protein levels of M1 markers CD86 and iNOS were significantly downregulated, whereas M2 markers CD206 and Arg-1 were upregulated in the Lut@ZIF-8-PT group (Figure 4e), as confirmed by densitometric analysis (Figure S14, see online supplementary material). RT-qPCR analysis aligned with protein-level results, showing that Lut@ZIF-8-PT treatment significantly decreased transcription of pro-inflammatory genes IL-6 and TNF-α while increasing anti-inflammatory genes IL-10 and ARG-1 (Figure 4f–i).

Collectively, these results demonstrate that Lut@ZIF-8-PT effectively promotes macrophage polarization toward an anti-inflammatory M2-like phenotype, providing a favorable immunological microenvironment for tissue regeneration and wound healing.

### Attenuation of oxidative stress and transcriptomic insights into the underlying mechanism of Lut@ZIF-8-PT scaffold

To evaluate the antioxidative capacity of the Lut@ZIF-8-PT scaffold and explore its underlying molecular mechanisms in vascular endothelial cells, intracellular ROS levels were first assessed in HUVECs, followed by RNA sequencing (RNA-seq) analysis to characterize scaffold-induced transcriptomic alterations.

**Figure 5:**
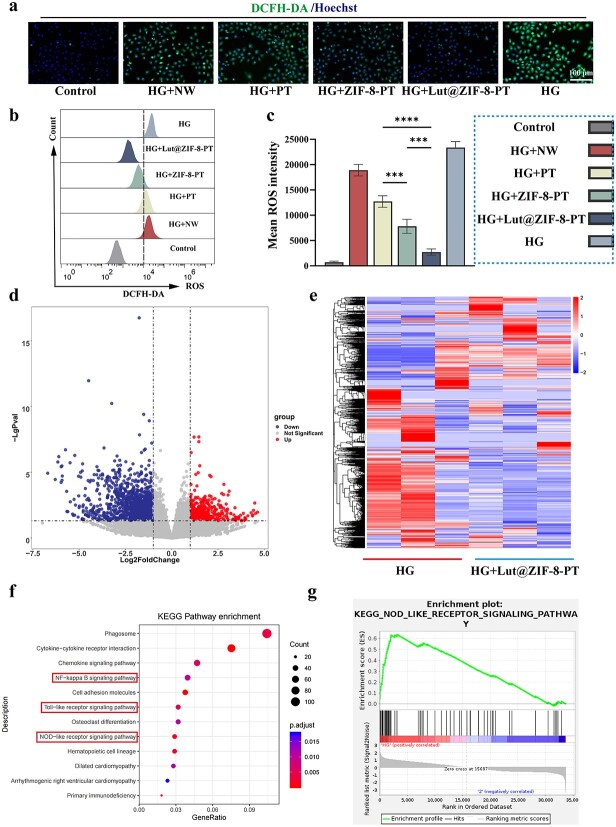
Lut@ZIF-8-PT scaffold reduces intracellular ROS levels and modulates pyroptosis-associated gene expression in HUVECs under high-glucose. (a) Representative fluorescence microscopy images showing intracellular ROS levels after treatment. Scale bar: 100 μm. (b) Quantification of ROS-positive cells by flow cytometry. (c) Quantification of ROS-positive cells by fluorescence microscopy images. (d) Volcano plot showing differentially expressed genes between treatment groups. (e) Heatmap of transcriptomic profiles highlighting top differentially expressed genes. (f) KEGG pathway enrichment analysis of significant gene sets. (g) Enrichment plot for pyroptosis-related pathways. Control: Control group; HG: High-glucose model group (30 mM); HG + NW: High-glucose+NW group; HG + PT: High-glucose+PT group; HG + ZIF-8-PT: High-glucose+ZIF-8-PT group; HG + Lut@ZIF-8-PT:High-glucose+Lut@ZIF-8-PT group, data are expressed as mean ± SD (n = 3 independent experiments). One-way ANOVA was used for single time-point comparisons, and FDR-adjusted p-values were applied in RNA-seq analyses. ^*^P < 0.05, ^**^P < 0.01, ^***^P < 0.001, ^****^P < 0.0001. *ZIF-8* zinc–imidazolate metal–organic frameworks, *Lut* luteolin, *HUVEC* human umbilical vein endothelial cells, *ANOVA* analysis of variance, *SD* standard deviation, *HG* high glucose, *NW* non-patterned fibrous membrane, *PT* patterned fibrous membrane, *ROS* reactive oxygen species, *DCFH-DA* 2′,7′-dichlorodihydrofluorescein diacetate, *KEEG* kyoto encyclopedia of genes and genomes, *FDR* false discovery rate

Fluorescent ROS staining revealed pronounced intracellular ROS accumulation in the control and NW groups, whereas HUVECs treated with Lut@ZIF-8-PT exhibited markedly reduced ROS fluorescence intensity, indicating effective attenuation of oxidative stress (Figure 5a). Consistently, flow cytometric analysis demonstrated a significant reduction in the proportion of ROS-positive cells in the Lut@ZIF-8-PT group compared with other treatments, further confirming the robust antioxidative effect of the composite scaffold (Figure 5b and c).

To gain deeper insight into the molecular basis underlying ROS suppression and endothelial protection, RNA-seq analysis was performed on HUVECs exposed to different scaffold formulations. The volcano plot revealed a distinct transcriptomic profile in the Lut@ZIF-8-PT group relative to the control, with a substantial number of genes showing significant upregulation or downregulation (Figure 5d). Hierarchical clustering analysis further demonstrated clear separation between treatment groups, highlighting the unique global gene expression pattern induced by Lut@ZIF-8-PT treatment (Figure 5e).

Functional enrichment analysis provided mechanistic insights into these transcriptomic changes. KEGG pathway enrichment revealed that differentially expressed genes were predominantly involved in inflammation- and pyroptosis-associated pathways, including NOD-like receptor signaling and TNF signaling pathways (Figure 5f). In addition, gene set enrichment plots confirmed significant modulation of oxidative stress–related pathways accompanied by suppression of programmed inflammatory cell death, suggesting coordinated regulation of redox balance and inflammatory responses (Figure 5g).

Collectively, these findings demonstrate that the Lut@ZIF-8-PT scaffold effectively attenuates oxidative stress in endothelial cells and induces broad transcriptomic reprogramming, with key enrichment in pathways related to inflammasome activation and pyroptosis. These results provide transcriptomic evidence supporting the subsequent mechanistic studies on NLRP3-mediated endothelial pyroptosis suppression.

### Inhibition of endothelial pyroptosis via suppression of the NLRP3-GSDMD Signaling pathway

To investigate the mechanisms by which the Lut@ZIF-8-PT scaffold regulates endothelial cell pyroptosis, we examined the expression and activation status of key pyroptosis-associated mediators, including NLRP3, GSDMD, and downstream inflammatory cytokines. A combination of immunofluorescence staining, RT-qPCR, and western blot analysis was employed to comprehensively assess the involvement of the NLRP3–GSDMD signaling axis.

**Figure 6:**
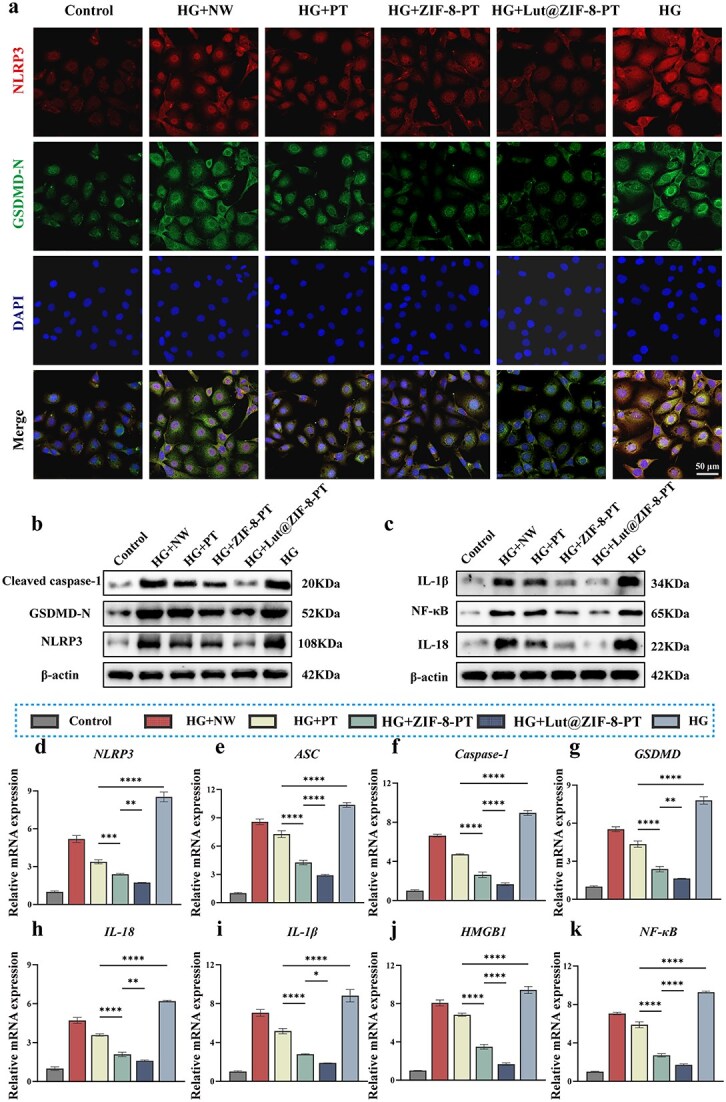
Lut@ZIF-8-PT scaffold inhibits pyroptosis in HUVECs through suppression of the NLRP3-GSDMD signaling pathway. (a) Immunofluorescence co-staining of NLRP3 and cleaved GSDMD (GSDMD-N) in HUVECs. Scale bar: 50 μm. (b) Western blot analysis of pyroptosis-related proteins including GSDMD-N, cleaved-Caspase-1, and NLRP3. (c) Expression of IL-1β, IL-18, and NF-κB proteins as assessed by western blot. (d–k) qPCR quantification of key genes involved in pyroptosis: IL-18, HMGB1, Caspase-1, ASC, NF-κB, IL-1β, GSDMD, and NLRP3. Control: Control group; HG: High-glucose model group (30 mM); HG + NW: High-glucose+NW group; HG + PT: High-glucose+PT group; HG + ZIF-8-PT: High-glucose+ZIF-8-PT group; HG + Lut@ZIF-8-PT:High-glucose+Lut@ZIF-8-PT group, data are presented as mean ± SD (n = 3 independent experiments). Statistical significance was determined using one-way ANOVA with Tukey’s post hoc test. ^*^P < 0.05, ^**^P < 0.01, ^***^P < 0.001, ^****^P < 0.0001. *NLRP3* NOD-like receptor family pyrin domain containing 3, *HUVEC* human umbilical vein endothelial cells, *ANOVA* analysis of variance, *SD* standard deviation, *ZIF-8* zinc–imidazolate metal–organic frameworks, *Lut* luteolin, *IL* interleukin, *TNF-α* tumor necrosis factor-alpha, *ARG-1* arginase-1, *HMG*B high mobility group box 1, *NF-κB* nuclear factor kappa-light-chain-enhancer of activated B cells, *GSDMD* gasdermin D

Immunofluorescence co-staining revealed pronounced colocalization of NLRP3 and cleaved GSDMD (GSDMD-N) in HUVECs exposed to high-glucose (HG) conditions and in the NW group, indicating activation of endothelial pyroptosis. In contrast, HUVECs treated with Lut@ZIF-8-PT exhibited markedly reduced fluorescence intensity and diminished colocalization of NLRP3 and GSDMD-N, suggesting effective suppression of NLRP3 inflammasome activation and subsequent GSDMD cleavage (Figure 6a). These observations were further validated by western blot analysis, which demonstrated that protein levels of cleaved caspase-1, GSDMD-N, and NLRP3 were significantly decreased in the Lut@ZIF-8-PT group (Figure 6b and c and Figure S15, see online supplementary material).

Consistent with the protein-level findings, RT-qPCR analysis revealed significant downregulation of pyroptosis- and inflammation-related genes, including IL-18, HMGB1, Caspase-1, ASC, NF-κB, IL-1β, GSDMD, and NLRP3, in HUVECs treated with the Lut@ZIF-8-PT scaffold compared with control groups (Figure 6d–k).

**Figure 7:**
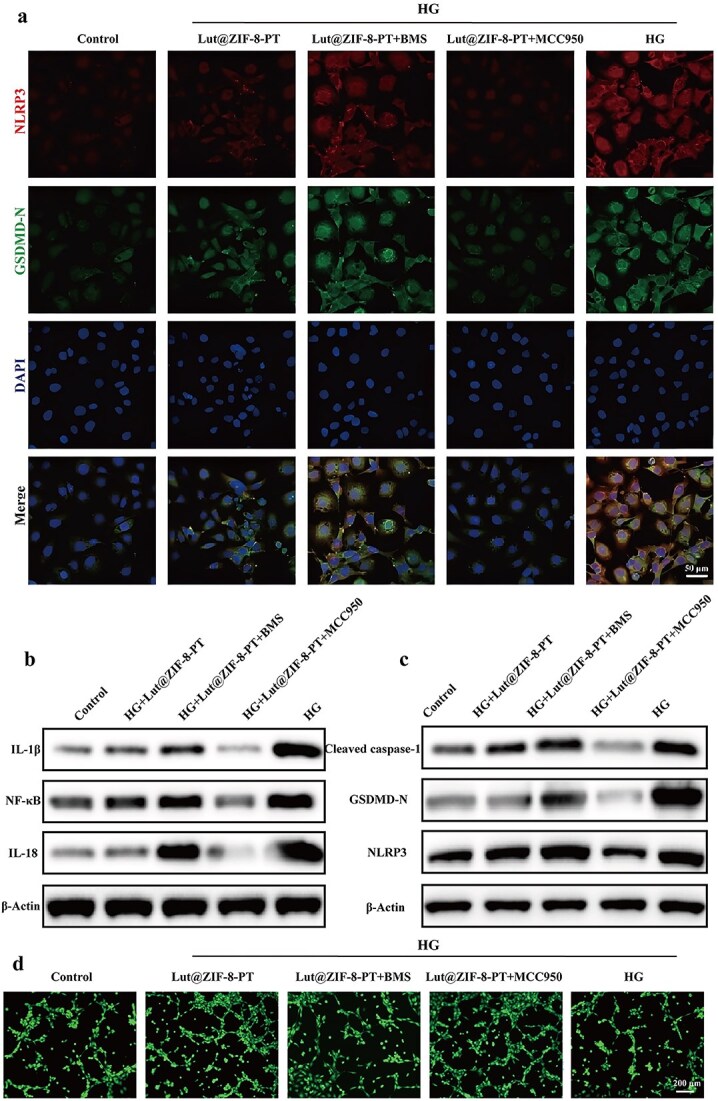
Lut@ZIF-8-PT scaffolds inhibit endothelial cell pyroptosis through NLRP3-GSDMD signaling pathway. (a) Immunofluorescence co-staining of NLRP3 (red) and cleaved GSDMD (GSDMD-N, green) with DAPI (blue) in HUVECs. Scale bar: 50 μm. (b) Western blot analysis of the inflammatory factors IL-1β, IL-18 and NF-κB in different treatment groups showed that Lut@ZIF-8-PT was significantly down-regulated, MCC950 further inhibited, and BMS further aggravated. (c) Western blot detection of cleaved caspase-1, GSDMD-N, and NLRP3, demonstrating inhibition of pyroptosis execution by Lut@ZIF-8-PT and enhanced effect with pharmacological inhibitors. (d) Representative fluorescent images of HUVEC tube formation under each treatment condition. Scale bar: 200 μm. Lut@ZIF-8-PT restored angiogenic capacity impaired by HG, and co-treatment with MCC950 further improved tube formation. Control: Control group; HG: High-glucose model group (30 mM); HG + Lut@ZIF-8-PT: High-glucose+Lut@ZIF-8-PT group; HG + Lut@ZIF-8-PT + BMS: High-glucose+Lut@ZIF-8-PT + BMS-98629 group (1 μM); HG + Lut@ZIF-8-PT + MCC950: High-glucose+Lut@ZIF-8-PT +  MCC950 group (1 μM), data are presented as mean ± SD (n = 3 independent experiments). Statistical significance was determined using one-way ANOVA with Tukey’s post hoc test. ^*^P < 0.05, ^**^P < 0.01, ^***^P < 0.001, ^****^P < 0.0001. *NLRP3* NOD-like receptor family pyrin domain containing 3, *HUVEC* human umbilical vein endothelial cells, *ANOVA* analysis of variance, *SD* standard deviation, *ZIF-8* zinc–imidazolate metal–organic frameworks, *Lut* luteolin, *IL* interleukin, *TNF-α* tumor necrosis factor-alpha, *ARG-1* arginase-1, *HMGB* high mobility group box 1, *NF-κB* nuclear factor kappa-light-chain-enhancer of activated B cells, *GSDMD* gasdermin D, *HG* high glucose, *NW* non-patterned fibrous membrane, *PT* patterned fibrous membrane

To further establish the causal role of the NLRP3 inflammasome in Lut@ZIF-8-PT–mediated inhibition of endothelial pyroptosis, rescue experiments were conducted using pharmacological modulators of NLRP3. Specifically, HUVECs were treated with BMS-986299 (NLRP3 agonist) or MCC950 (selective NLRP3 inhibitor) during Lut@ZIF-8-PT exposure, followed by HG stimulation to induce pyroptosis. Immunofluorescence co-staining showed strong colocalization of NLRP3 and GSDMD-N in the HG and BMS-986299-treated groups, confirming robust activation of pyroptosis. In contrast, Lut@ZIF-8-PT treatment markedly attenuated NLRP3 and GSDMD-N signals, and co-treatment with MCC950 further reduced fluorescence intensity, indicating enhanced suppression of endothelial pyroptosis upon NLRP3 inhibition (Figure 7a).

**Figure 8:**
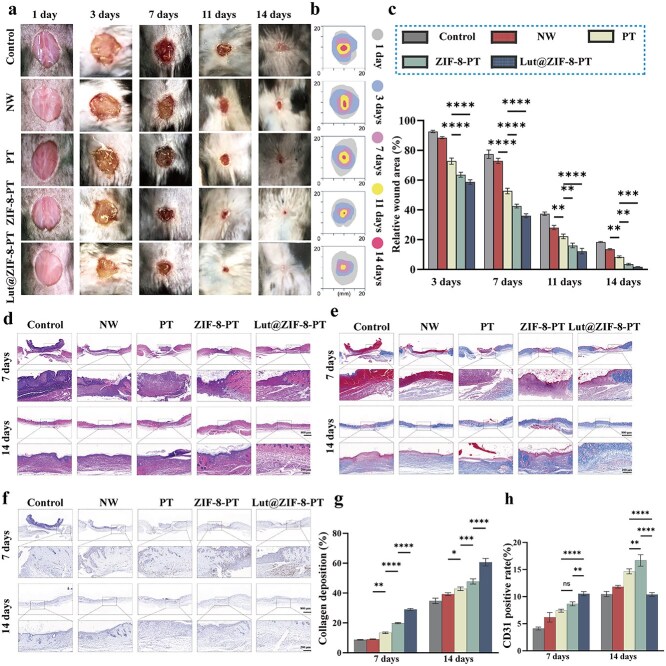
Lut@ZIF-8-PT scaffold promotes *in vivo* wound healing in diabetic mice. (a) Representative images showing wound size changes over 1, 3, 7, 11, and 14 days. (b) Simulated healing trajectories for each group. (c) Quantitative analysis of wound area percentage during healing. (d) H&E staining of wound sections. Scale bar: 800 μm & 200 μm. (e) Masson’s trichrome staining of wound sections. Scale bar: 800 μm & 200 μm. (f) CD31 immunohistochemical staining of neovascular structures in wound tissue. Scale bar: 800 μm & 200 μm. (g) Quantification of collagenous fiber deposition based on Masson’s trichrome staining. (h) Quantification of CD31-positive vessel density. Data are shown as mean ± SD (n = 5 mice per group). Control: STZ group; NW: STZ + NW group; PT: STZ + PT group; ZIF-8-PT: STZ + ZIF-8-PT group; Lut@ZIF-8-PT: STZ + Lut@ZIF-8-PT group, statistical significance was determined using two-way repeated-measures ANOVA (time × treatment) for wound closure curves and one-way ANOVA for single time-point comparisons, followed by Bonferroni or Tukey’s post hoc test as appropriate. ^*^P < 0.05, ^**^P < 0.01, ^***^P < 0.001, ^****^P < 0.0001. *H&E* hematoxylin and eosin, *STZ* streptozotocin, *ZIF-8* zinc–imidazolate metal–organic frameworks, *Lu*t luteolin, *GSDMD* gasdermin D, *HG* high glucose, *NW* non-patterned fibrous membrane, *PT* patterned fibrous membrane, *ANOVA* analysis of variance, *SD* standard deviation

**Figure 9:**
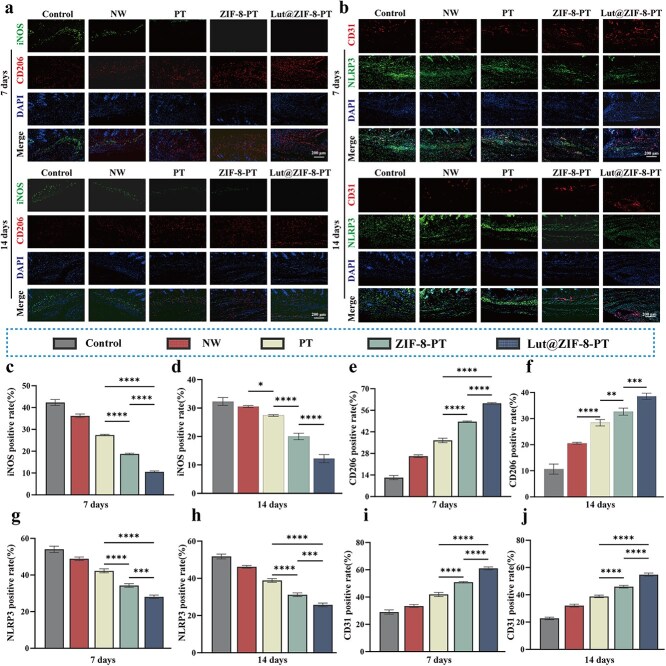
Lut@ZIF-8-PT scaffold modulates macrophage polarization, inhibits endothelial pyroptosis, and enhances angiogenesis in diabetic wounds. (a) Immunofluorescence staining of iNOS (green, M1 macrophages) and CD206 (red, M2 macrophages) in wound tissues at days 7 and 14. Scale bar: 200 μm. (b) Immunofluorescence co-staining of CD31 (red, endothelial cells) with NLRP3 (green) in wound tissues at days 7 and 14, showing localization of pyroptotic signaling within endothelial cells. Scale bar: 200 μm. (c-f) Quantification of iNOS (c: 7 day; d: Day 14) and CD206 (e: Day 7; f: Day 14) fluorescence intensity in different treatment groups, demonstrating reduced M1 and increased M2 macrophage infiltration after Lut@ZIF-8-PT treatment. (g-h) Quantification of NLRP3 fluorescence intensity at days 7 (g) and 14 (h), showing significant suppression of endothelial inflammasome activation by the scaffold. (i-j) Quantification of CD31 fluorescence intensity at days 7 (i) and 14 (j), indicating enhanced endothelial cell proliferation and neovascularization in the Lut@ZIF-8-PT group. Control: STZ group; NW: STZ + NW group; PT: STZ + PT group; ZIF-8-PT: STZ + ZIF-8-PT group; Lut@ZIF-8-PT: STZ + Lut@ZIF-8-PT group, data are presented as mean ± SD (n = 3 mice per group). Statistical significance was determined using one-way ANOVA with Tukey’s post hoc test. ^*^P < 0.05, ^**^P < 0.01, ^***^P < 0.001, ^****^P < 0.0001. *ZIF-8* zinc–imidazolate metal–organic frameworks, *Lut* luteolin, *LPS* lipopolysaccharide, *iNOS* inducible nitric oxide synthase, *DAPI* 4′,6-diamidino-2-phenylindole, *ARG-1* arginase-1, *IL* interleukin, *SD* standard deviation, *HG* high glucose, *NW* non-patterned fibrous membrane, *PT* patterned fibrous membrane, *ANOVA* analysis of variance, *NLRP3* NOD-like receptor family pyrin domain containing 3

Western blot analysis corroborated these findings, showing that cleaved caspase-1, GSDMD-N, and NLRP3 protein levels were significantly elevated under HG stimulation but markedly reduced following Lut@ZIF-8-PT treatment. Notably, co-treatment with MCC950 resulted in a further decrease in pyroptosis-related protein expression, confirming the central involvement of the NLRP3–GSDMD axis (Figure 7b and c). In parallel, the expression of pro-inflammatory mediators, including IL-1β, IL-18, and NF-κB, was significantly downregulated in Lut@ZIF-8-PT–treated cells, with additional suppression observed upon MCC950 co-treatment.

Functional assays further demonstrated that endothelial angiogenic capacity was markedly impaired under HG conditions, as evidenced by reduced tube formation. Lut@ZIF-8-PT treatment significantly restored angiogenic activity, and pre-treatment with MCC950 further enhanced tube formation, indicating that inhibition of endothelial pyroptosis contributes to the pro-angiogenic effects of the scaffold (Figure 7d).

Collectively, these results demonstrate that Lut@ZIF-8-PT inhibits endothelial cell pyroptosis predominantly through suppression of the NLRP3–GSDMD signaling pathway. Rescue experiments using both an NLRP3 agonist and a selective inhibitor further substantiate the causal relationship between inflammasome modulation and the protective and pro-angiogenic effects of the scaffold.

### Lut@ZIF-8-PT scaffold accelerates cutaneous wound healing in diabetic mice

The therapeutic efficacy of Lut@ZIF-8-PT was systematically evaluated in streptozotocin-induced diabetic mice using a full-thickness excisional wound model. Macroscopic examination revealed that wounds treated with Lut@ZIF-8-PT exhibited significantly accelerated closure over a 14-day period compared with the NW, PT, and ZIF-8-PT groups (Figure 8a–c). Simulated wound healing trajectories further confirmed faster epithelialization in the Lut@ZIF-8-PT group.

Histological analyses using H&E and Masson’s trichrome staining demonstrated markedly enhanced re-epithelialization, reduced inflammatory cell infiltration, and denser, more organized collagen deposition in the Lut@ZIF-8-PT-treated wounds (Figure 8d–e). Importantly, H&E staining of major organs revealed no apparent histopathological abnormalities (Figure S16, see online supplementary material), indicating favorable in vivo biosafety. In parallel, CD31 immunohistochemical staining showed a significantly increased microvessel density in the Lut@ZIF-8-PT group, indicative of enhanced angiogenesis (Figure 8f). Simultaneous quantitative assessment of collagen fiber deposition based on Masson’s trichrome staining and CD31-positive vessel density also demonstrated that in the wounds of the Lut@ZIF-8-PT treatment group, collagen deposition was more compact and angiogenesis was enhanced (Figure 8g and h).

Immunofluorescence analysis of macrophage phenotypes revealed a pronounced reduction in iNOS-positive M1 macrophages and a concomitant increase in CD206-positive M2 macrophages in wounds treated with Lut@ZIF-8-PT, suggesting effective modulation of the inflammatory microenvironment toward a reparative phenotype (Figure 9a). To further elucidate the molecular basis of these effects, co-immunostaining of the pyroptosis marker NLRP3 with the endothelial marker CD31 was performed. Lut@ZIF-8-PT treatment markedly reduced NLRP3 expression in CD31-positive endothelial cells compared with the control and NW groups, indicating suppression of endothelial pyroptosis (Figure 9b).

Quantitative analyses performed at days 7 and 14 post-injury confirmed a significantly decreased M1/M2 macrophage ratio and increased endothelial cell density in the Lut@ZIF-8-PT group (Figure 9c–j). Collectively, these findings demonstrate that Lut@ZIF-8-PT accelerates diabetic wound healing by attenuating endothelial pyroptosis via the NLRP3–GSDMD pathway, promoting M2 macrophage polarization, and enhancing angiogenesis and extracellular matrix remodeling, thereby facilitating the proliferative and remodeling phases of cutaneous repair.

## Discussion

In this study, we developed a Lut-loaded ZIF-8 nanoplatform (Lut@ZIF-8) and further integrated it into a patterned PCL/Gel nanofibrous scaffold (Lut@ZIF-8-PT) to address the multifactorial barriers of diabetic wound healing. Structural and compositional analyses confirmed successful Lut incorporation and preservation of ZIF-8 crystallinity, indicating good stability of the nanocarrier system. Importantly, Lut@ZIF-8-PT enabled sustained co-release of Lut and Zn^2+^ over an extended period without an initial burst, which is advantageous for maintaining long-term bioactivity in the hostile diabetic wound microenvironment where free small molecules are rapidly cleared.

The Lut@ZIF-8-PT scaffold exhibited excellent biocompatibility toward endothelial cells and macrophages, supporting its suitability for biomedical application. Beyond cytocompatibility, the scaffold significantly enhanced endothelial cell migration and angiogenic potential, suggesting a direct contribution to revascularization, a key limiting factor in diabetic wound repair.

Macrophage-mediated immune regulation represents another critical determinant of wound healing outcomes. Our results demonstrate that Lut@ZIF-8-PT effectively promoted macrophage polarization toward an anti-inflammatory, pro-regenerative M2 phenotype, accompanied by suppression of M1-associated markers. This immunomodulatory effect is particularly relevant for diabetic wounds, which are characterized by prolonged inflammation and impaired transition to the proliferative phase.

Mechanistically, the beneficial immunoregulatory and pro-angiogenic effects of Lut@ZIF-8-PT were closely associated with suppression of oxidative stress and inflammasome activation. The scaffold markedly reduced intracellular ROS accumulation in endothelial cells, thereby limiting activation of the NLRP3 inflammasome and downstream GSDMD-mediated pyroptosis. Inhibition of endothelial pyroptosis is expected to reduce the release of pro-inflammatory cytokines such as IL-1β and IL-18, consequently mitigating M1 macrophage recruitment and facilitating a pro-healing immune microenvironment. This ROS–NLRP3–pyroptosis–macrophage polarization axis provides a coherent mechanistic framework linking endothelial protection to immune modulation and tissue regeneration.

Transcriptomic analyses further supported these mechanistic insights, revealing downregulation of oxidative stress- and pyroptosis-related gene networks alongside upregulation of angiogenesis-associated pathways. These findings were corroborated by molecular validation of key inflammasome components and inflammatory mediators, consistent with previous reports implicating the AGEs/ROS/NLRP3 axis in diabetic wound pathology [13] and identifying endothelial pyroptosis as a major obstacle to angiogenesis [41].

The therapeutic relevance of Lut@ZIF-8-PT was confirmed in a diabetic mouse wound model, where the scaffold significantly accelerated wound closure, enhanced collagen deposition, promoted neovascularization, and favorably remodeled the immune microenvironment. Compared with previously reported hydrogel dressings or single-agent delivery systems [42, 43], this platform uniquely integrates sustained bioactive co-delivery, antioxidative regulation, inflammasome inhibition, and immune modulation within a single scaffold, enabling simultaneous targeting of interconnected pathological processes in diabetic wounds.

Despite these promising findings, several limitations should be acknowledged. Additional rescue experiments, such as pharmacological inhibition of NLRP3, would further strengthen causal validation of the proposed mechanism. Moreover, evaluation in infection-relevant wound models and long-term safety studies in large animals will be necessary to advance clinical translation.

In summary, this work highlights the rational design and multifunctional therapeutic potential of the Lut@ZIF-8-PT scaffold for diabetic wound healing. By coordinating sustained drug delivery, immune regulation, suppression of oxidative stress and endothelial pyroptosis, and promotion of angiogenesis, this system offers an integrated strategy to overcome key barriers in chronic wound repair and provides a solid foundation for future translational development.

## Conclusions

Overall, this study demonstrates that Lut@ZIF-8-PT patterned nanofibrous scaffolds serve as an effective multifunctional platform for diabetic wound healing by integrating sustained Lut/Zn^2+^ co-release, immunomodulation, and antioxidative regulation. The scaffolds promote angiogenesis and collagen deposition, accelerate wound closure, and alleviate chronic inflammation by suppressing ROS–NLRP3–GSDMD–mediated endothelial pyroptosis while driving macrophage polarization toward a pro-regenerative M2 phenotype. These findings highlight the potential of nanofibrous scaffold–based strategies to simultaneously target multiple pathological barriers in chronic wounds and support their future translational application in diabetic wound management and related inflammatory tissue repair.

## Supplementary Material

Supplementary_Figure_1_tkag005

Supplementary_Figure_2_tkag005

Supplementary_Figure_3_tkag005

Supplementary_Figure_4_tkag005

Supplementary_Figure_5_tkag005

Supplementary_Figure_6_tkag005

Supplementary_Figure_7_tkag005

Supplementary_Figure_8_tkag005

Supplementary_Figure_9_tkag005

Supplementary_Figure_10_tkag005

Supplementary_Figure_11_tkag005

Supplementary_Figure_12_tkag005

Supplementary_Figure_13_tkag005

Supplementary_Figure_14_tkag005

Supplementary_Figure_15_tkag005

Supplementary_Figure_16_tkag005

Supplementary_table_1_tkag005

Supplementary_table_2_tkag005

Supplementary_table_3_tkag005

SUPPORTING_INFOMATION_tkag005
