# Supplementary figures and images for: Multifunctional nanofibrous membranes enhance diabetic wound healing by inhibiting endothelial pyroptosis and regulating macrophage polarization
Source: Burns Trauma. 2026 Jan 19;14:tkag005. doi: 10.1093/burnst/tkag005 (PMC13011808; doi:10.1093/burnst/tkag005)

**a**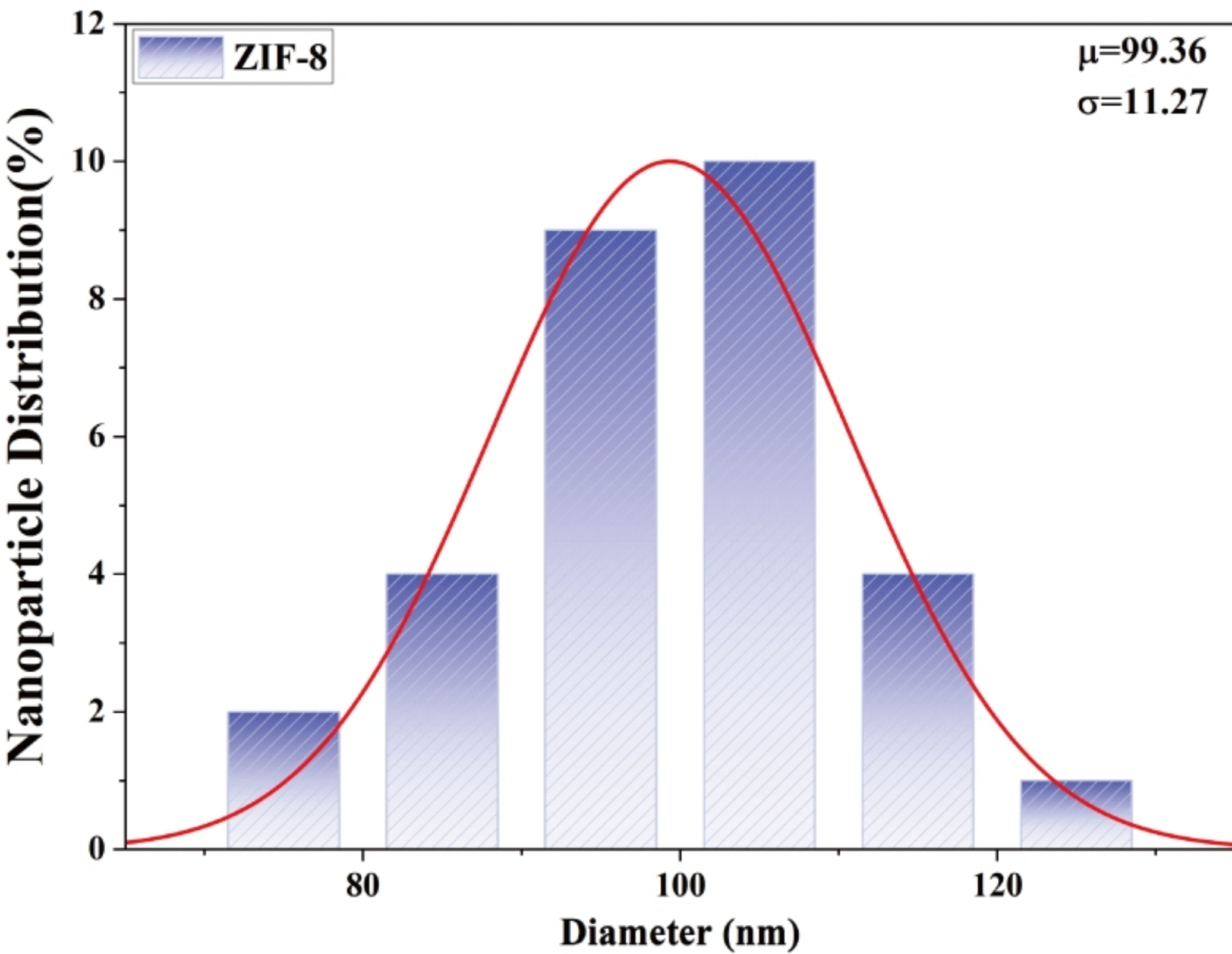**b**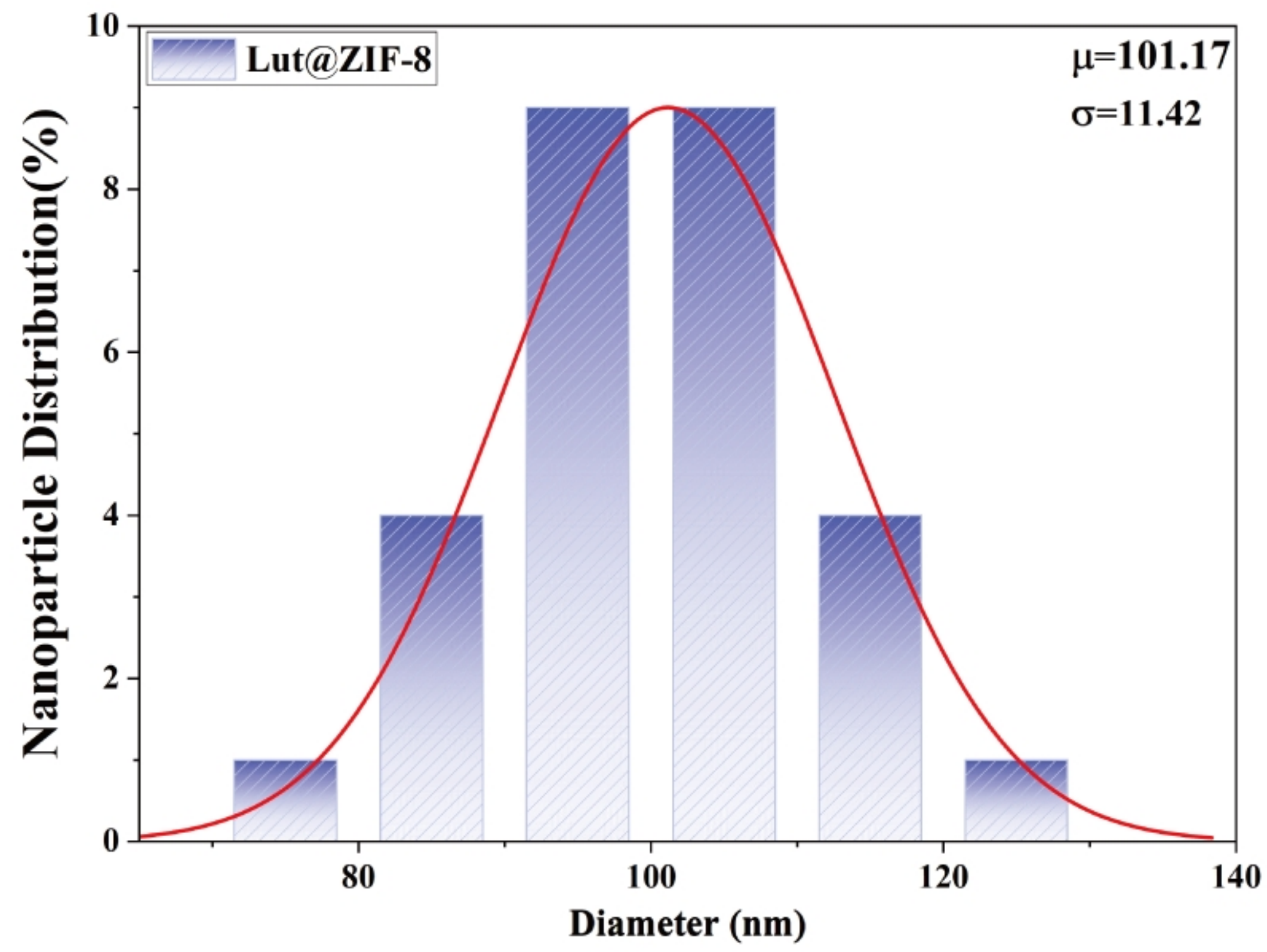

Supplement: Supplementary_Figure_1_tkag005 [file supplementary_figure_1_tkag005.pdf]

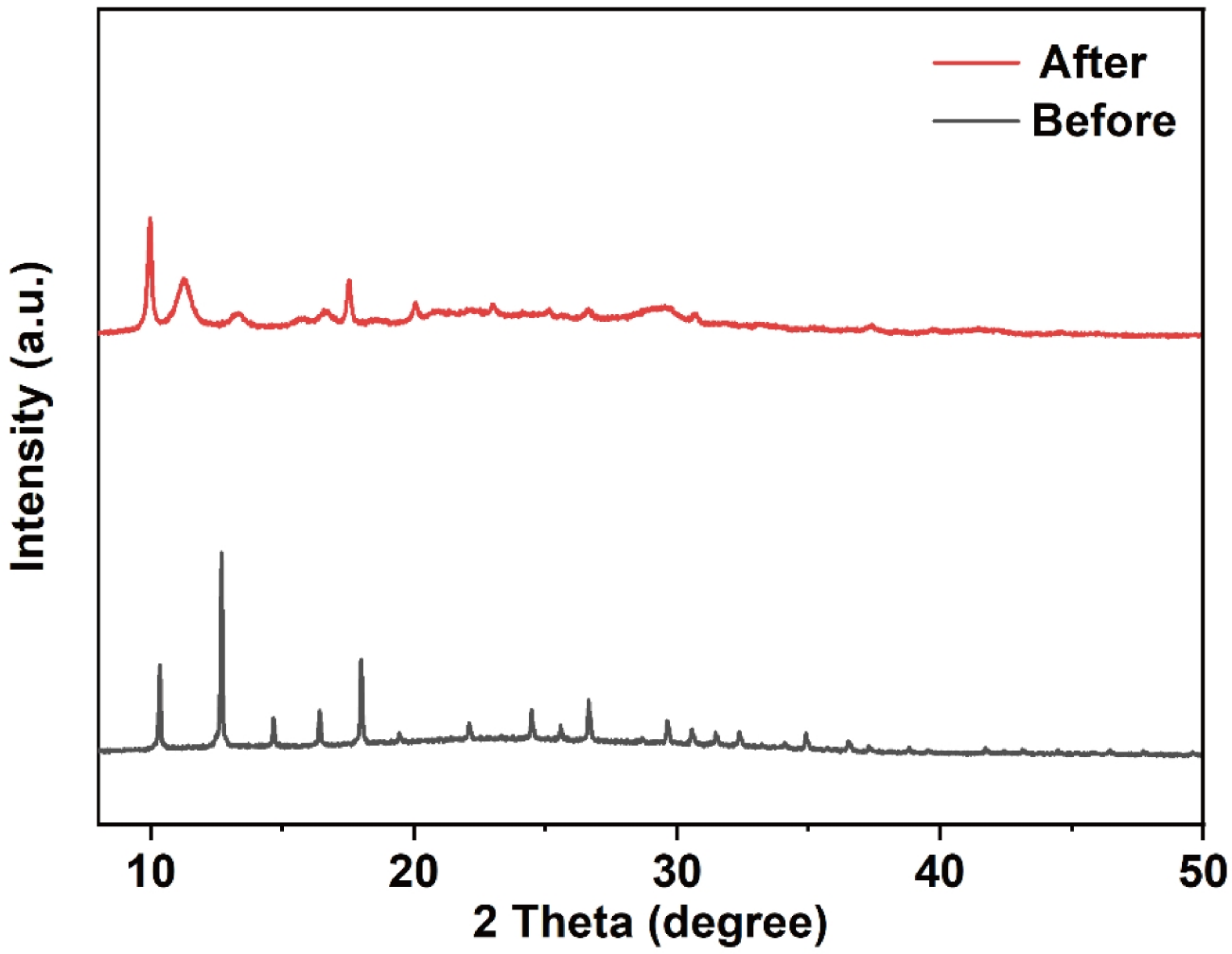

Supplement: Supplementary_Figure_2_tkag005 [file supplementary_figure_2_tkag005.pdf]

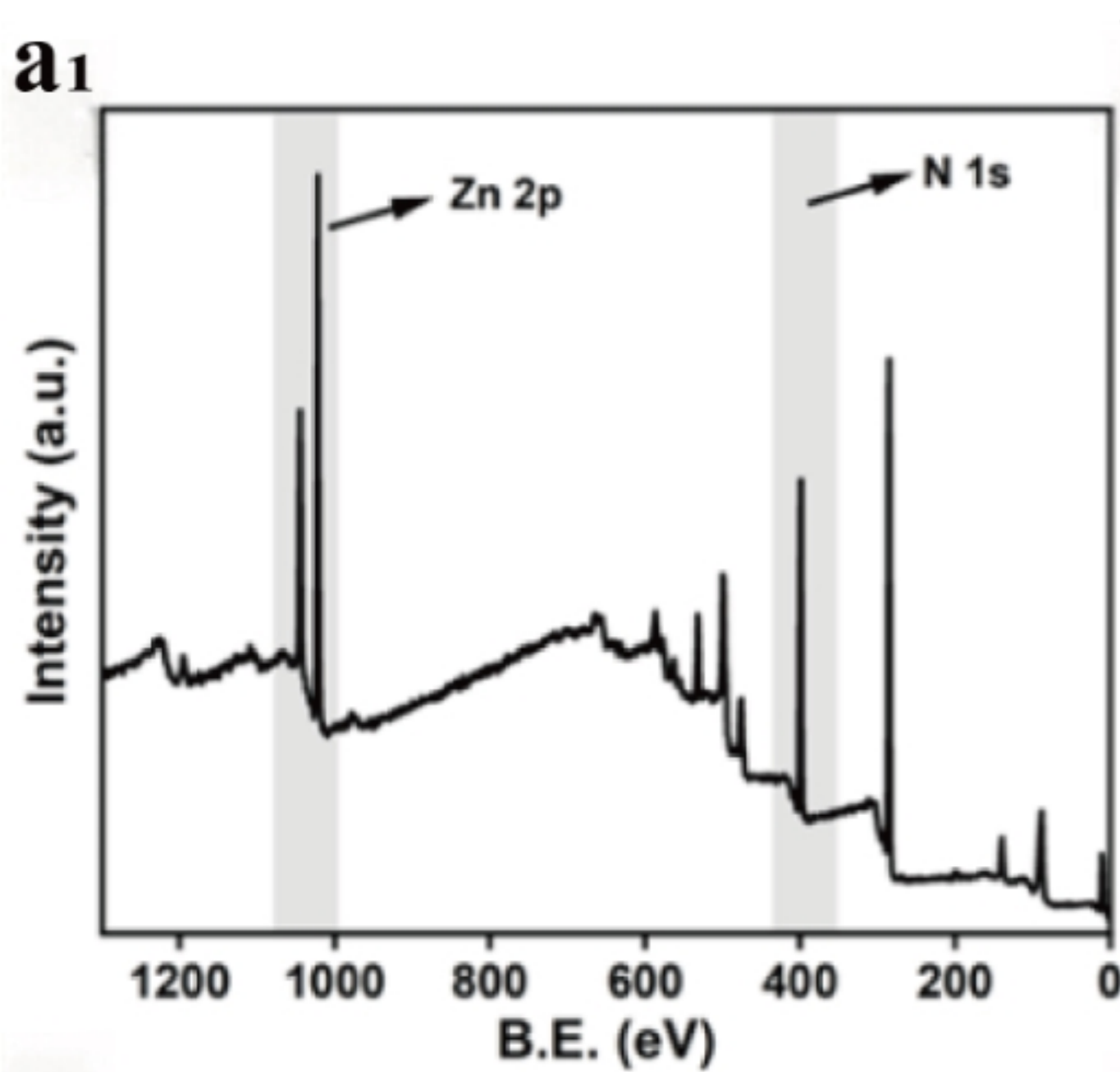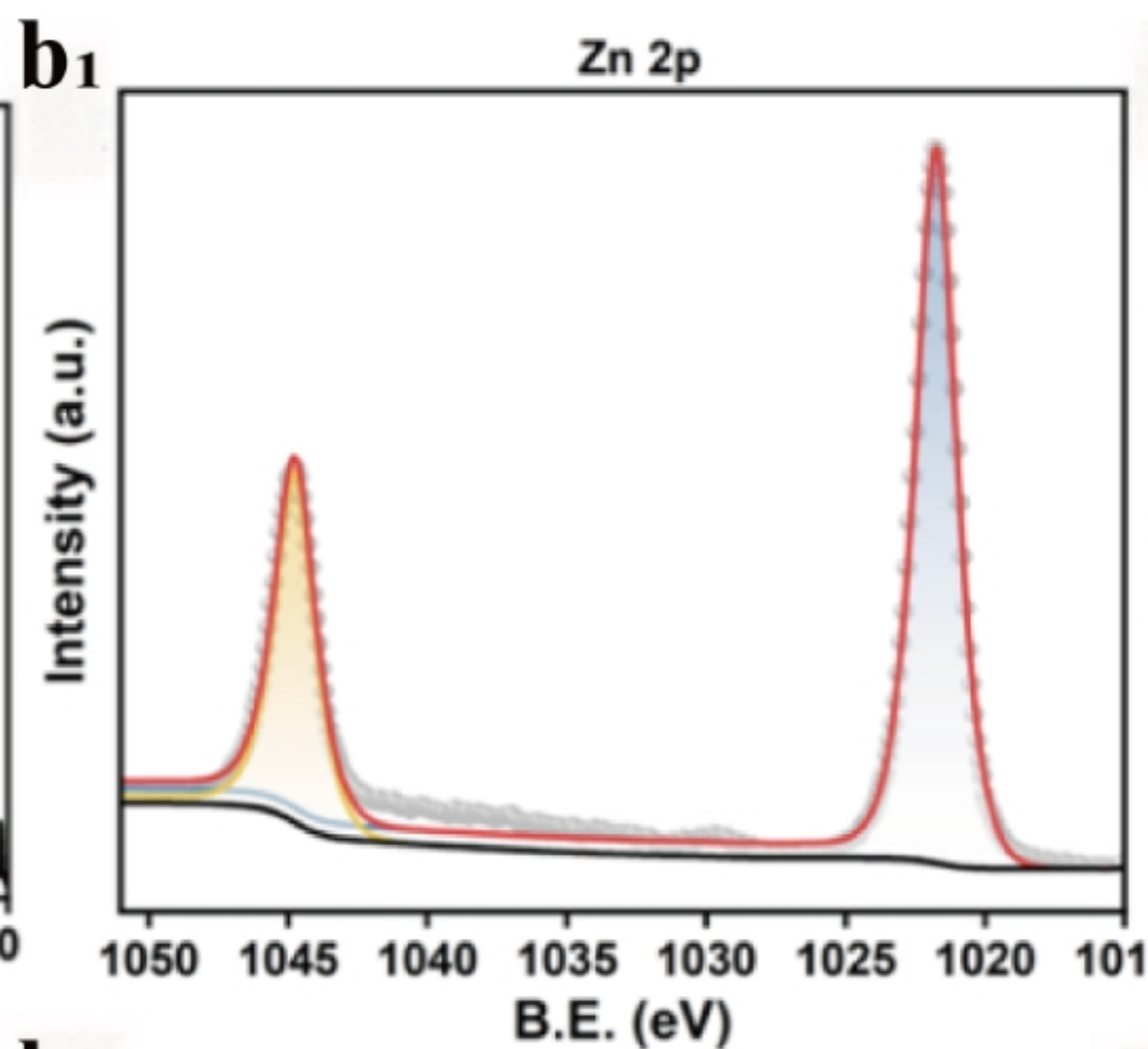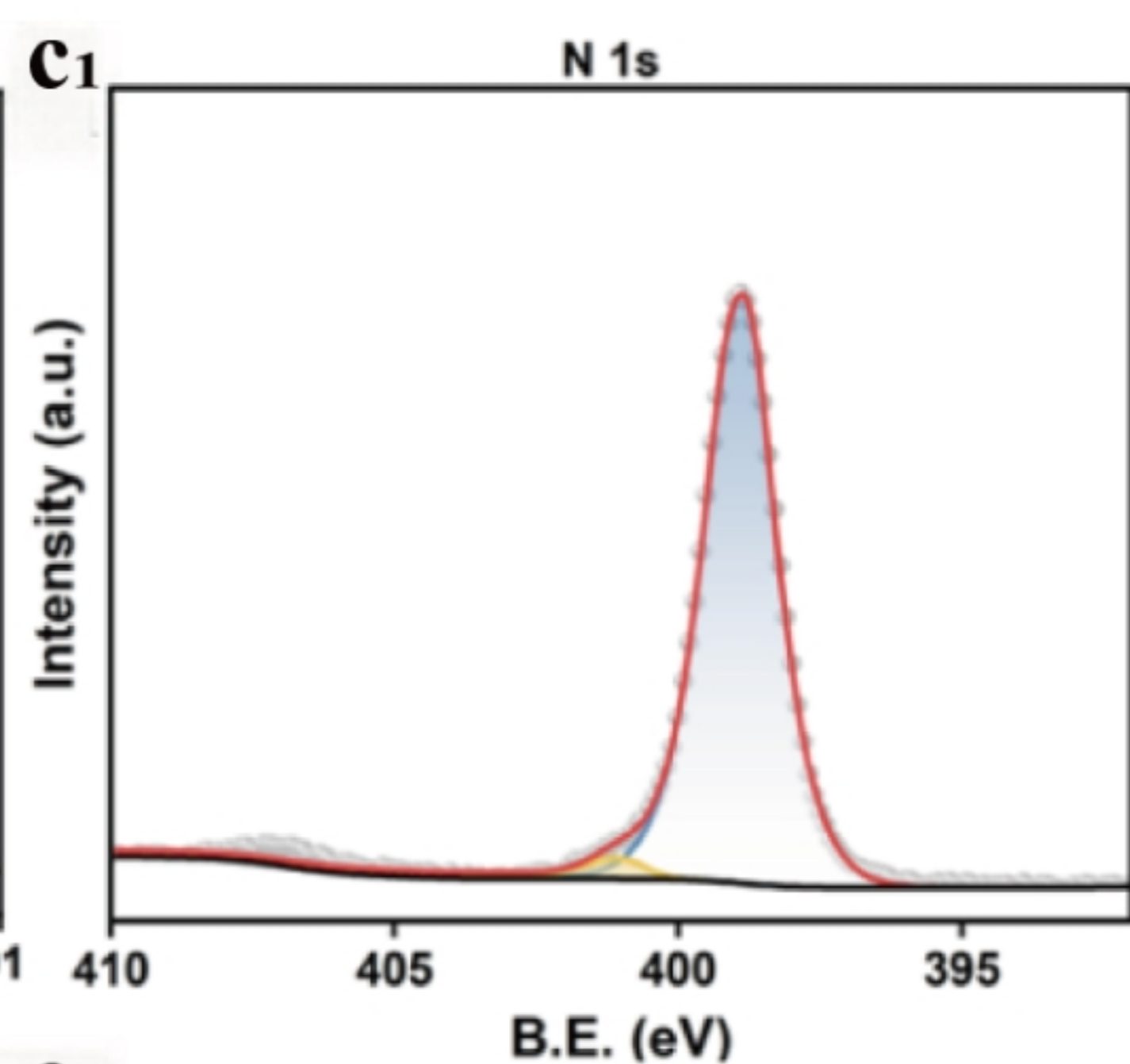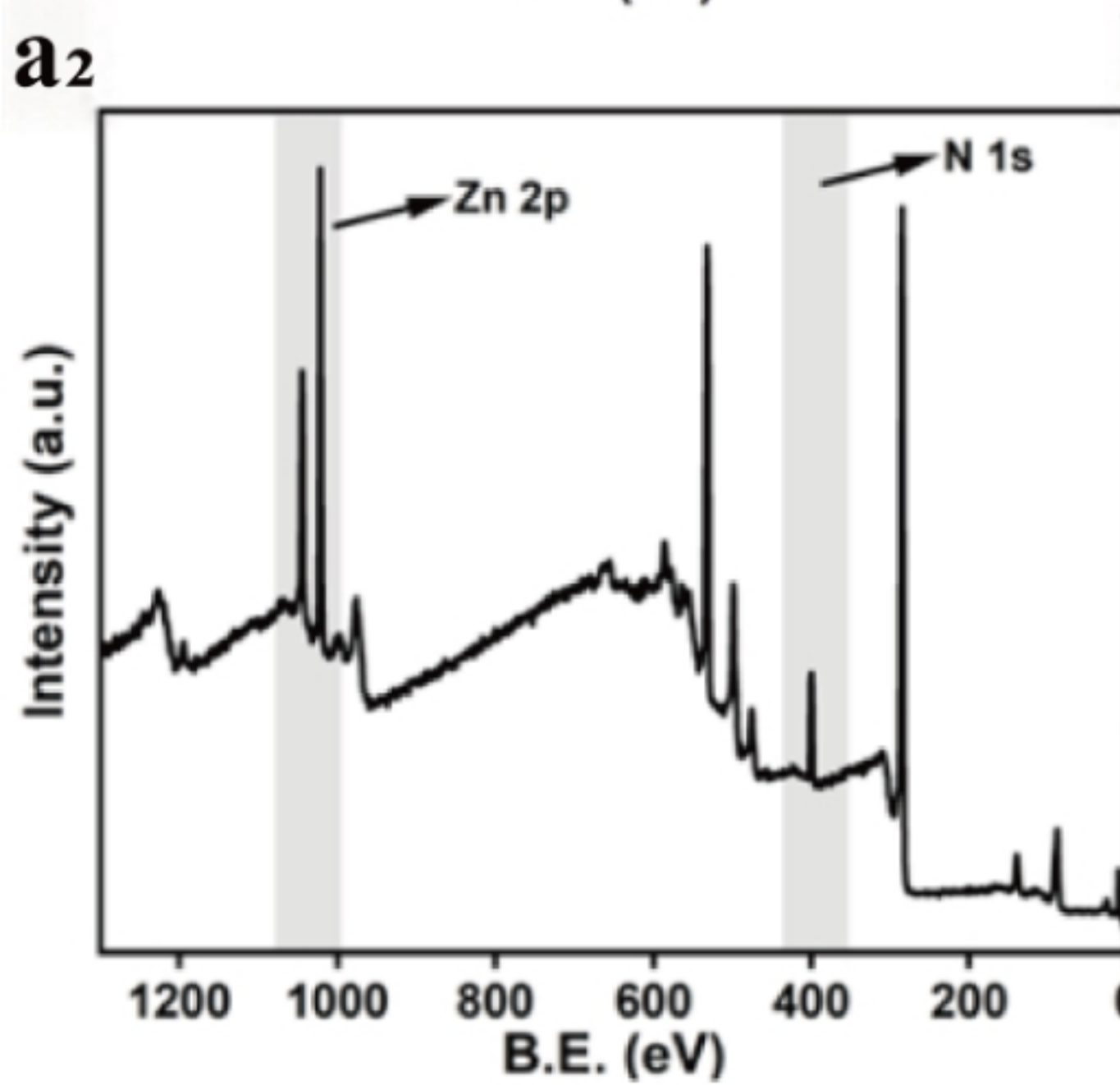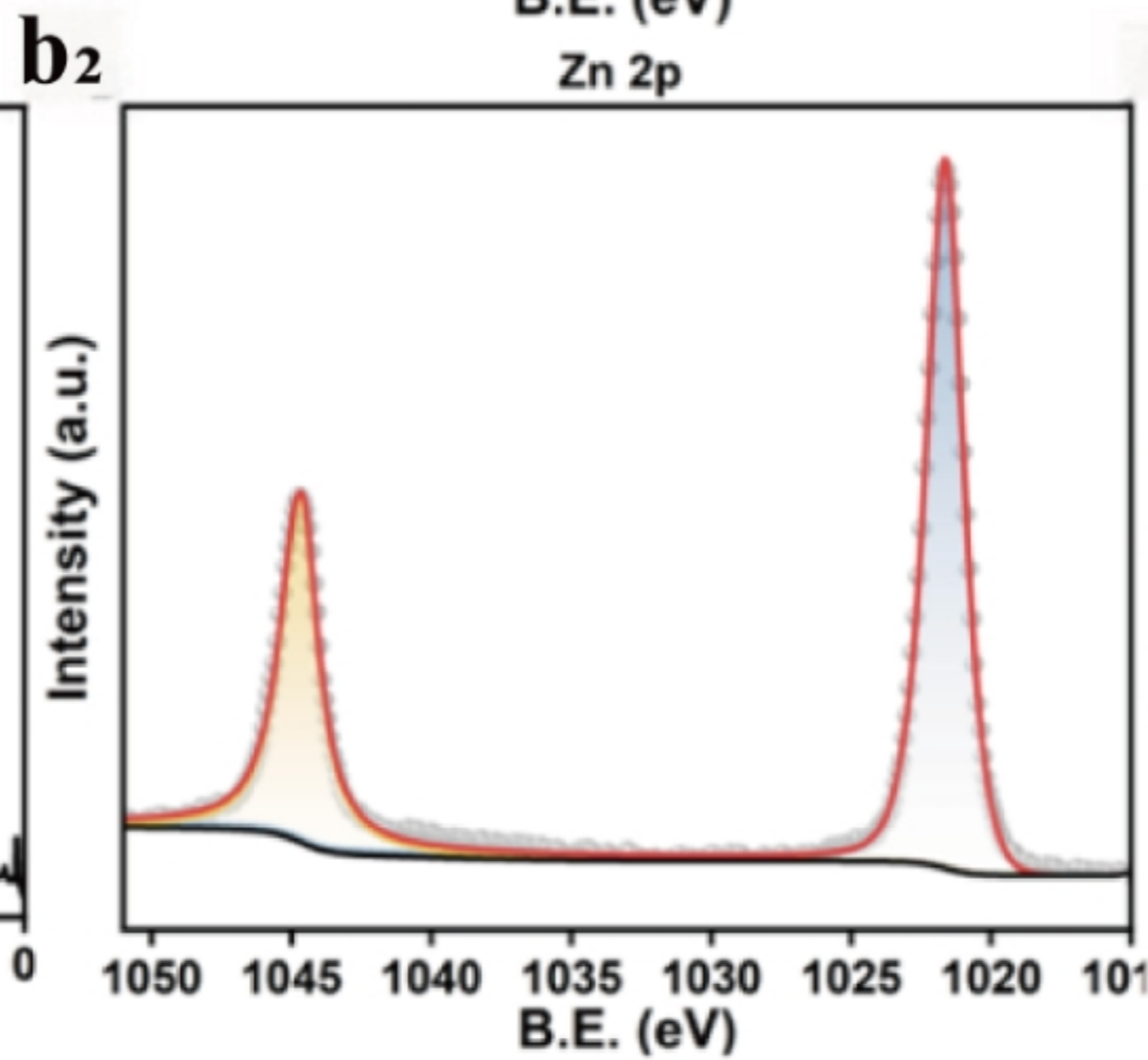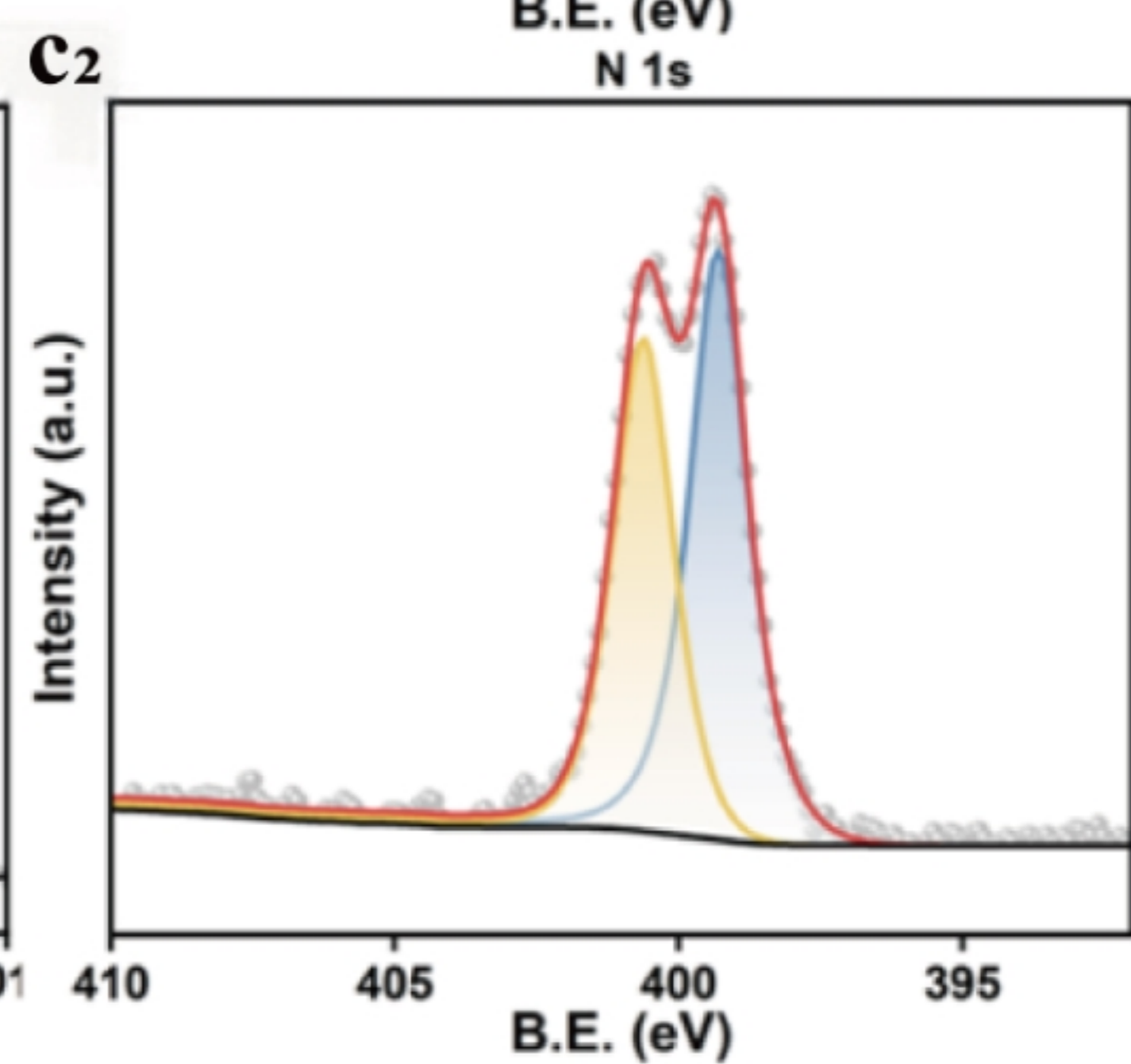

Supplement: Supplementary_Figure_3_tkag005 [file supplementary_figure_3_tkag005.pdf]

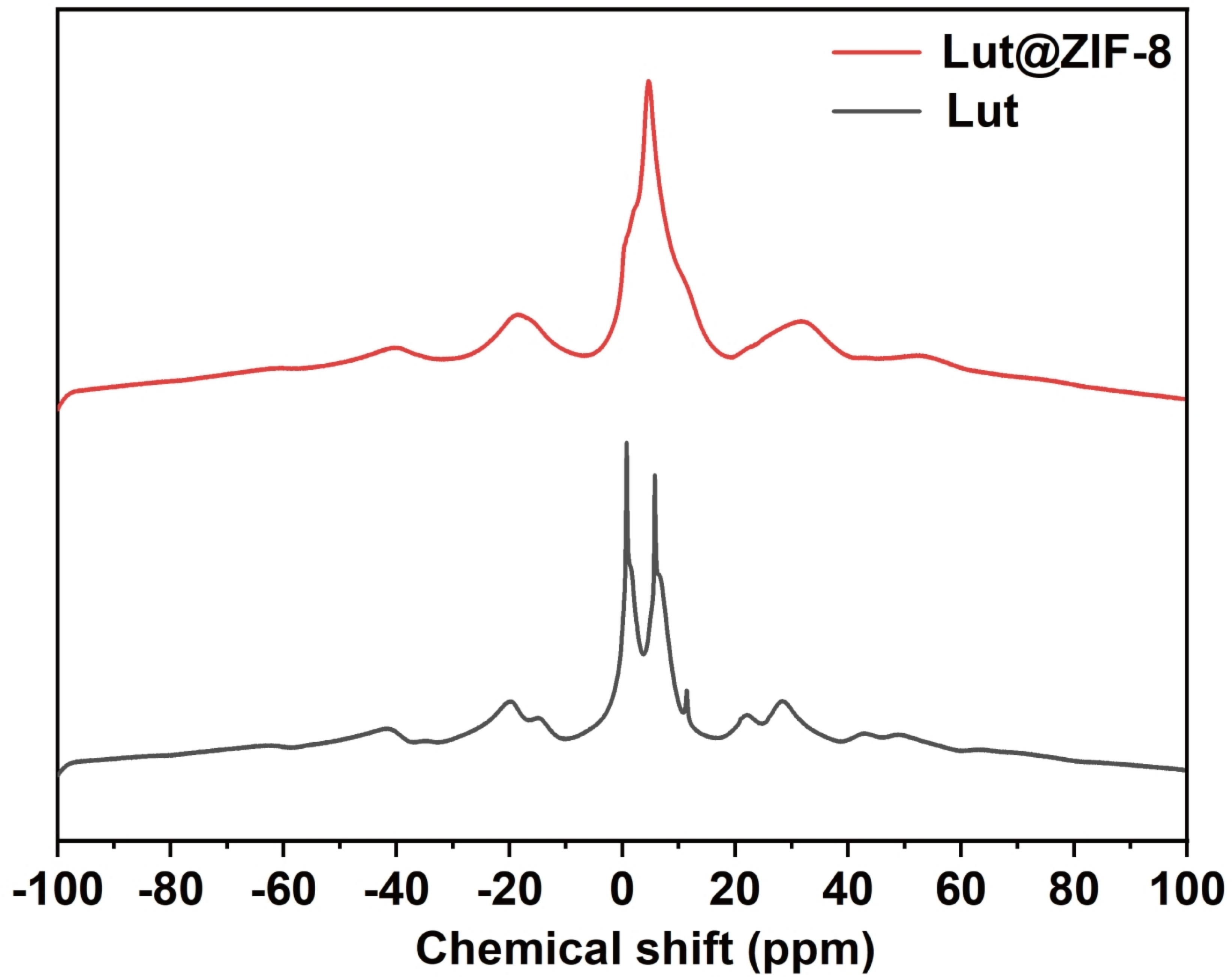

Supplement: Supplementary_Figure_4_tkag005 [file supplementary_figure_4_tkag005.pdf]

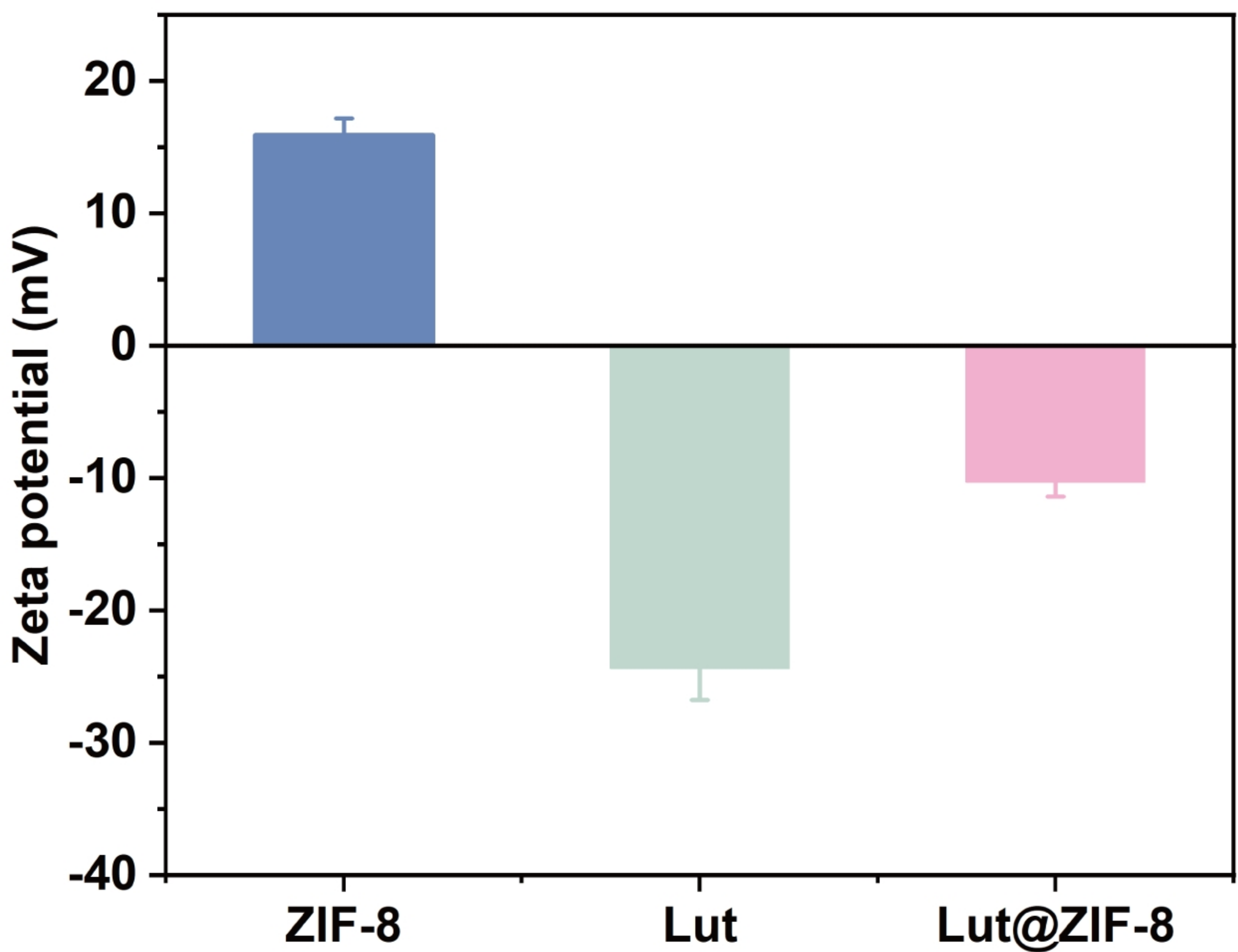

Supplement: Supplementary_Figure_5_tkag005 [file supplementary_figure_5_tkag005.pdf]

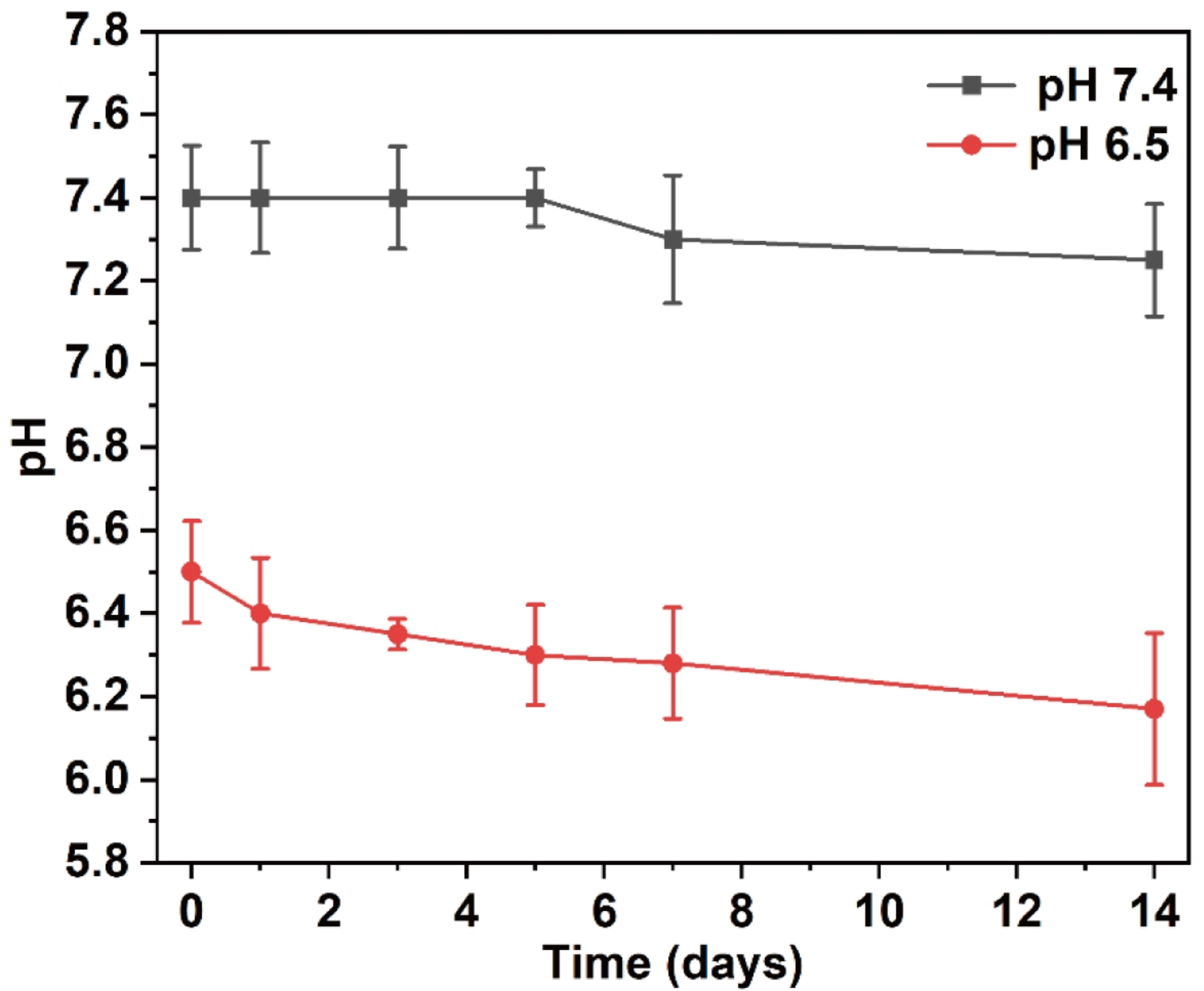

Supplement: Supplementary_Figure_6_tkag005 [file supplementary_figure_6_tkag005.pdf]

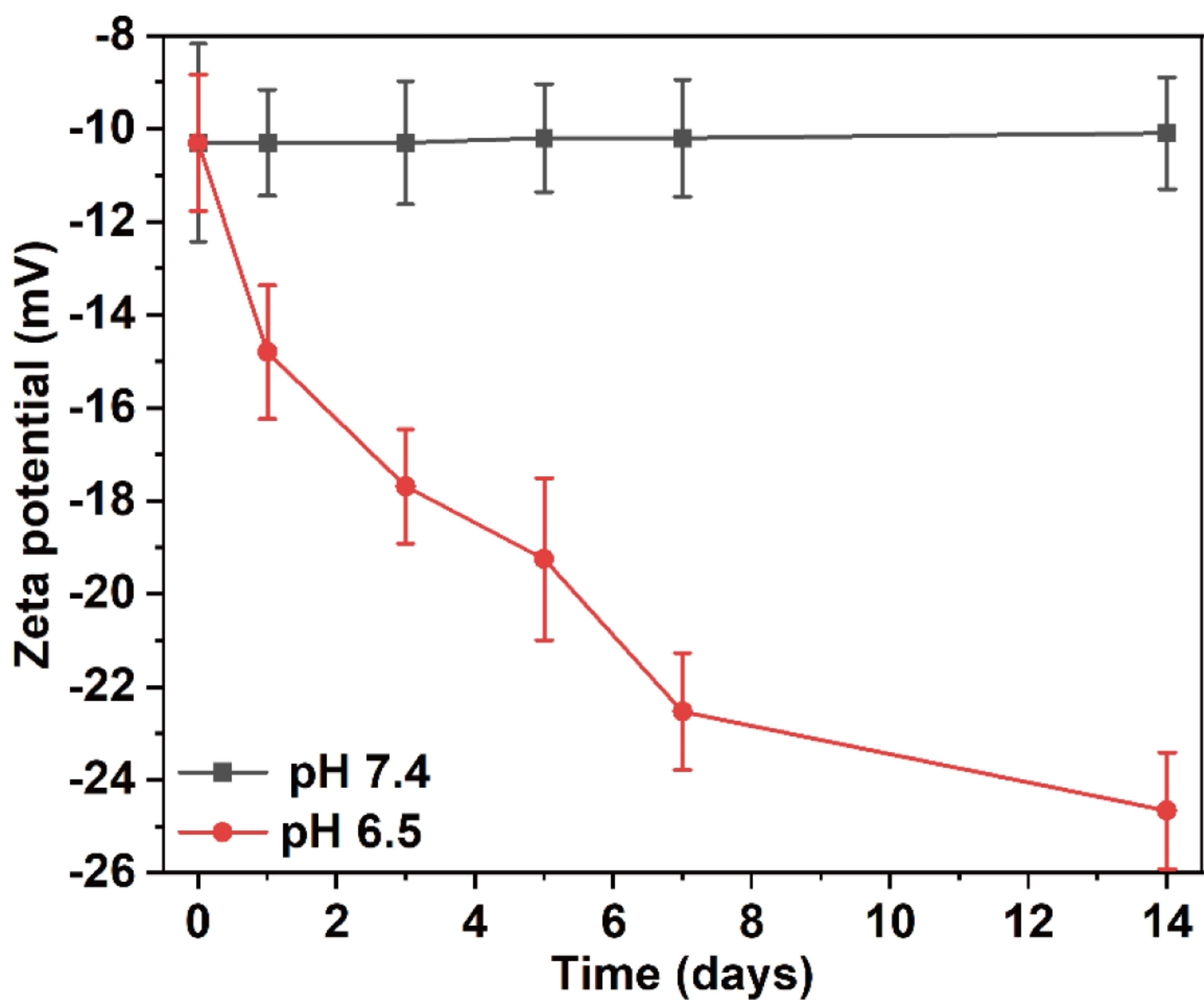

Supplement: Supplementary_Figure_7_tkag005 [file supplementary_figure_7_tkag005.pdf]

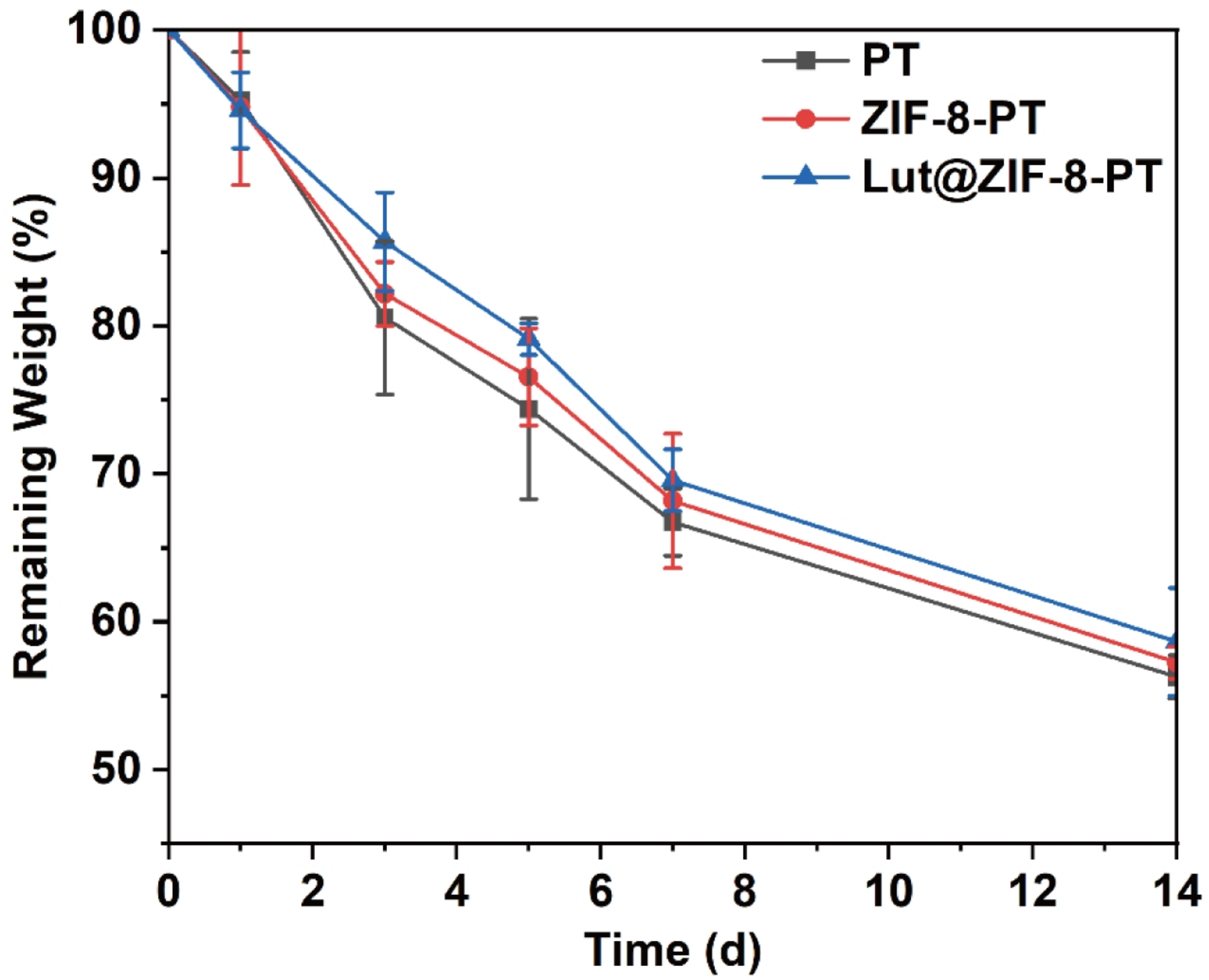

Supplement: Supplementary_Figure_8_tkag005 [file supplementary_figure_8_tkag005.pdf]

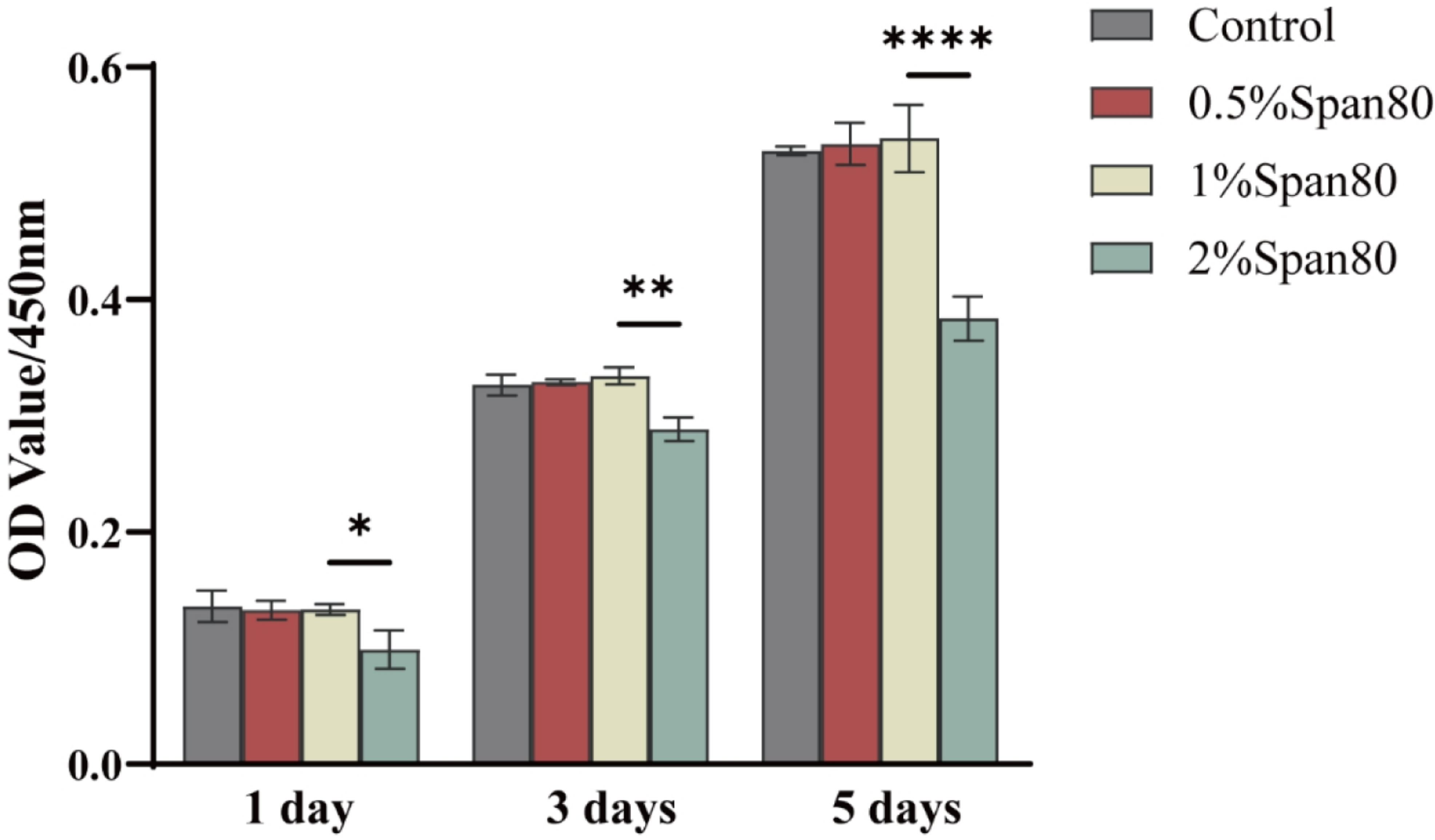

Supplement: Supplementary_Figure_9_tkag005 [file supplementary_figure_9_tkag005.pdf]

OD Value/450nm

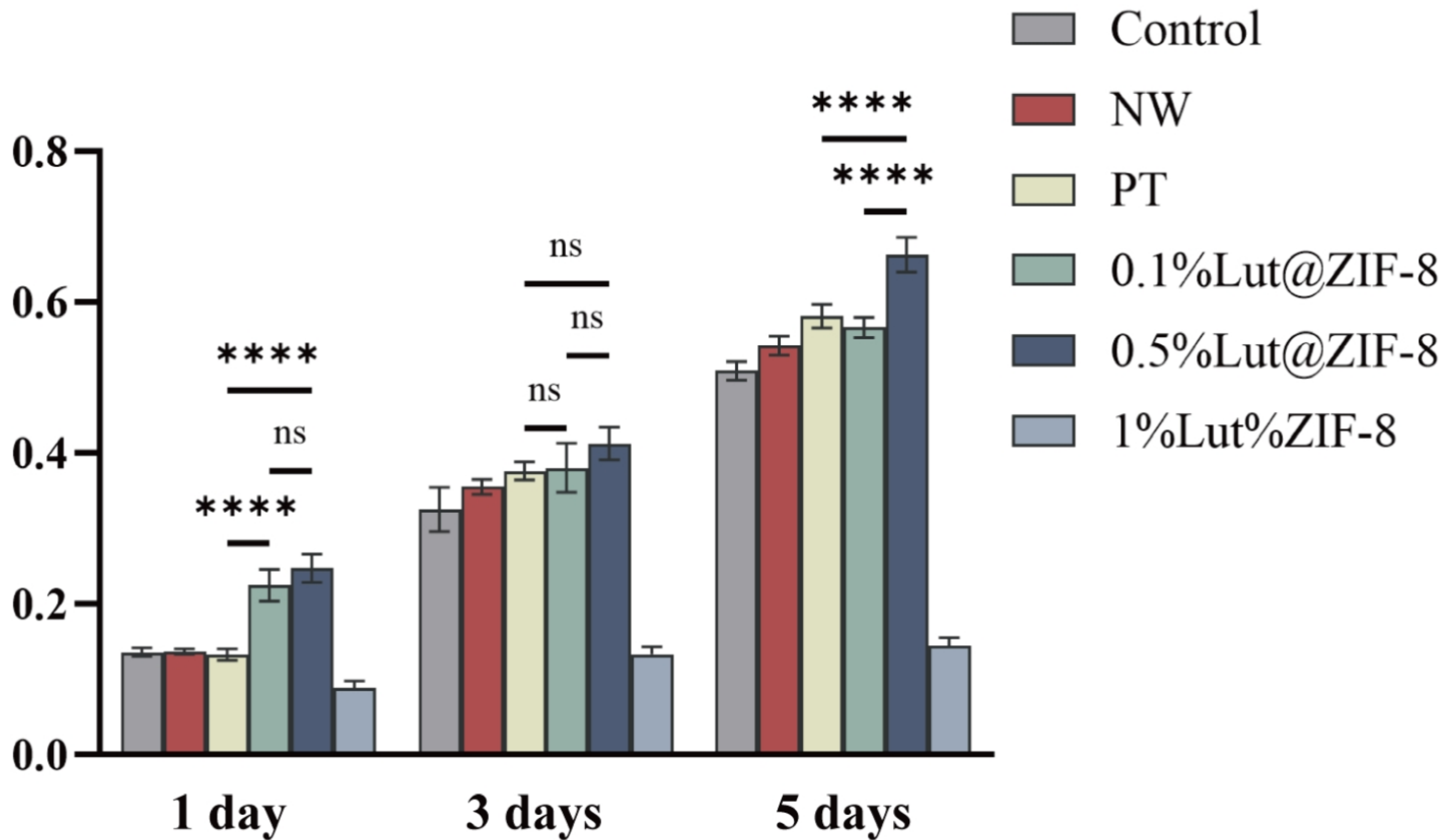

Supplement: Supplementary_Figure_10_tkag005 [file supplementary_figure_10_tkag005.pdf]

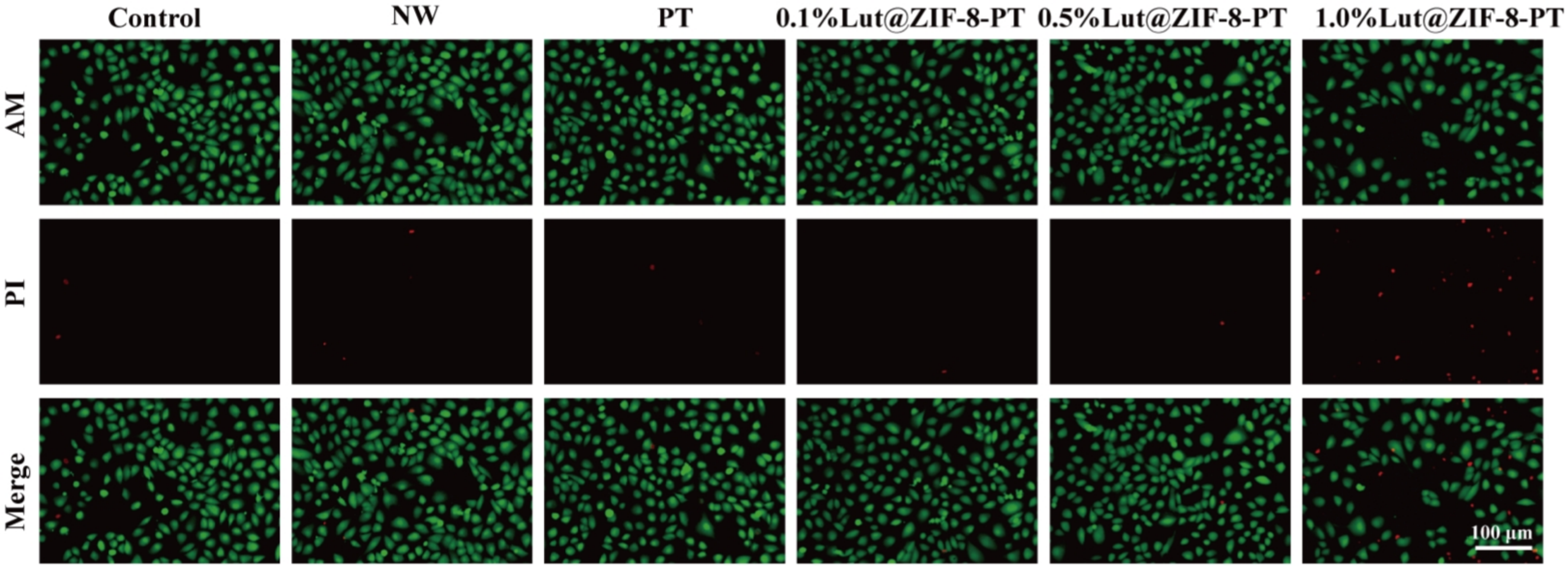

Supplement: Supplementary_Figure_11_tkag005 [file supplementary_figure_11_tkag005.pdf]

Hemolysis ratio (%)

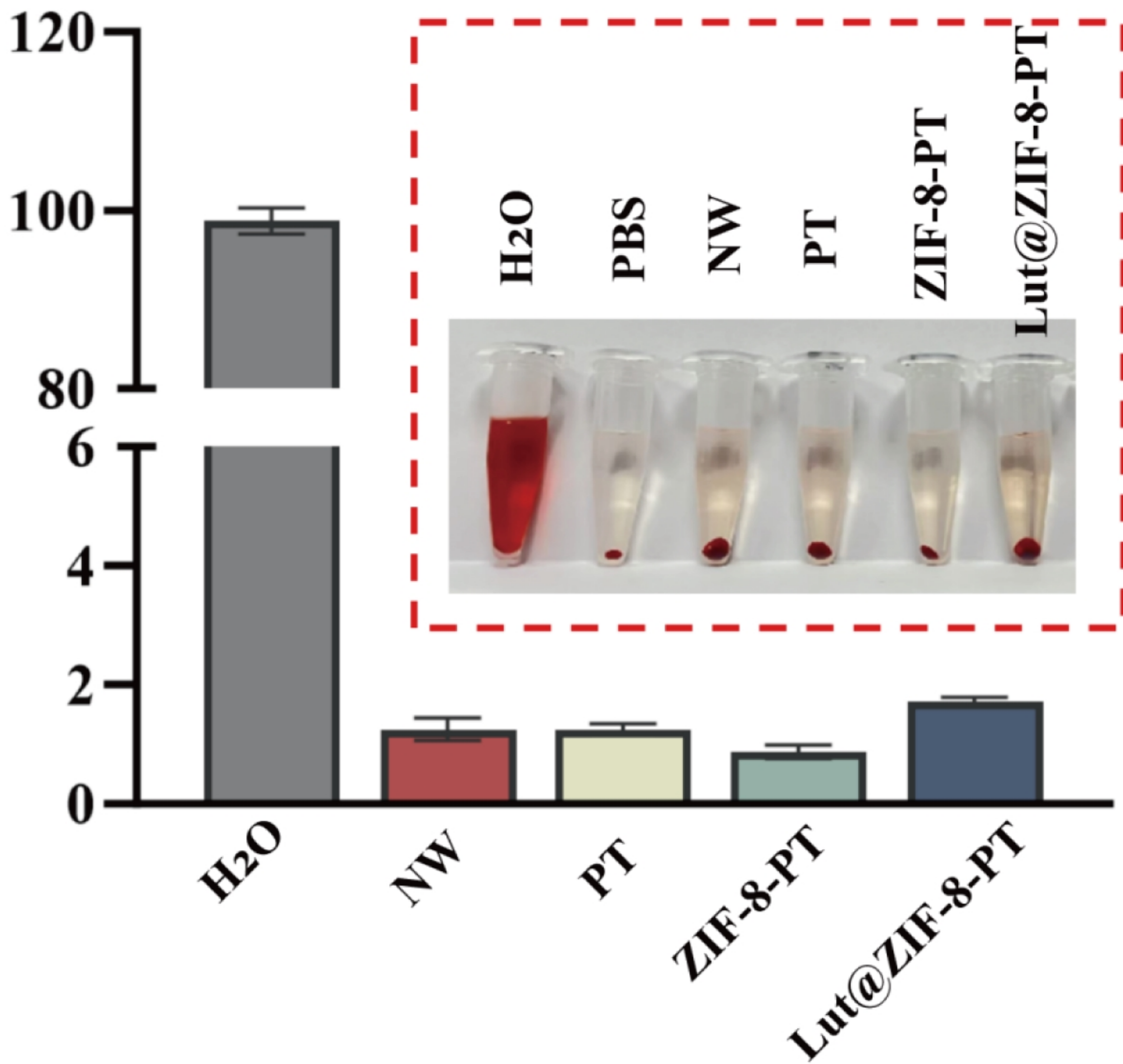

Supplement: Supplementary_Figure_12_tkag005 [file supplementary_figure_12_tkag005.pdf]

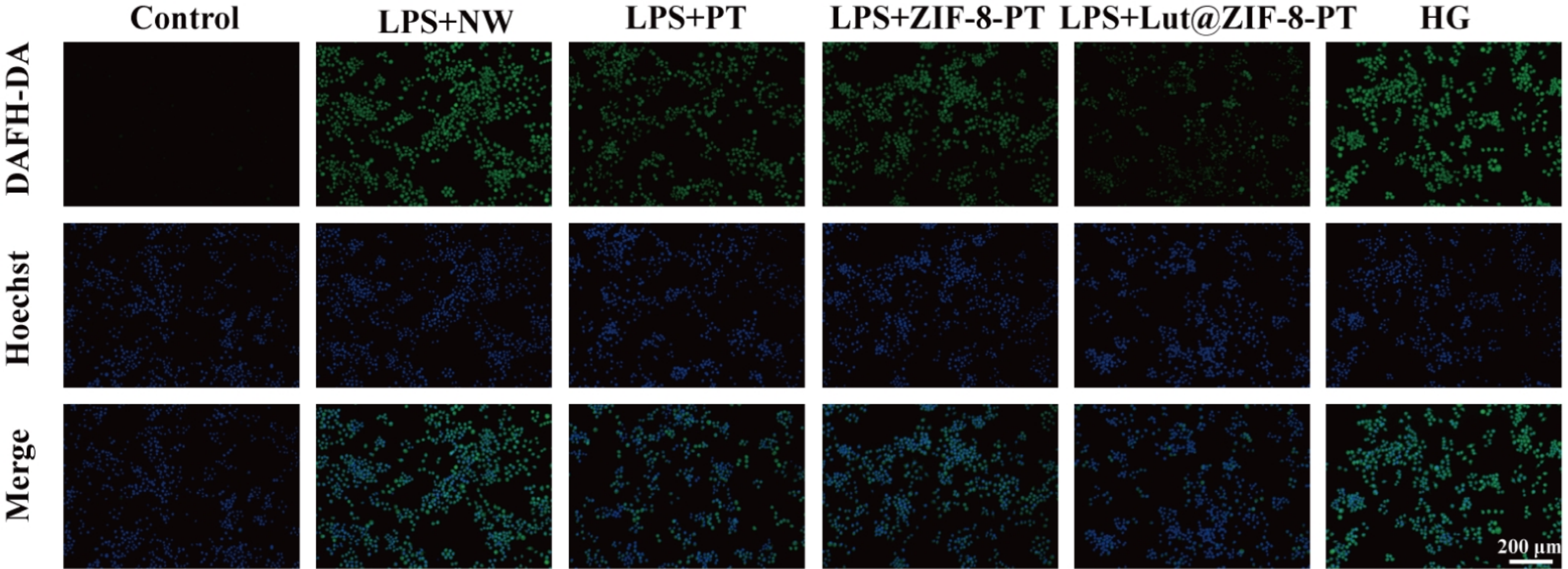

Supplement: Supplementary_Figure_13_tkag005 [file supplementary_figure_13_tkag005.pdf]

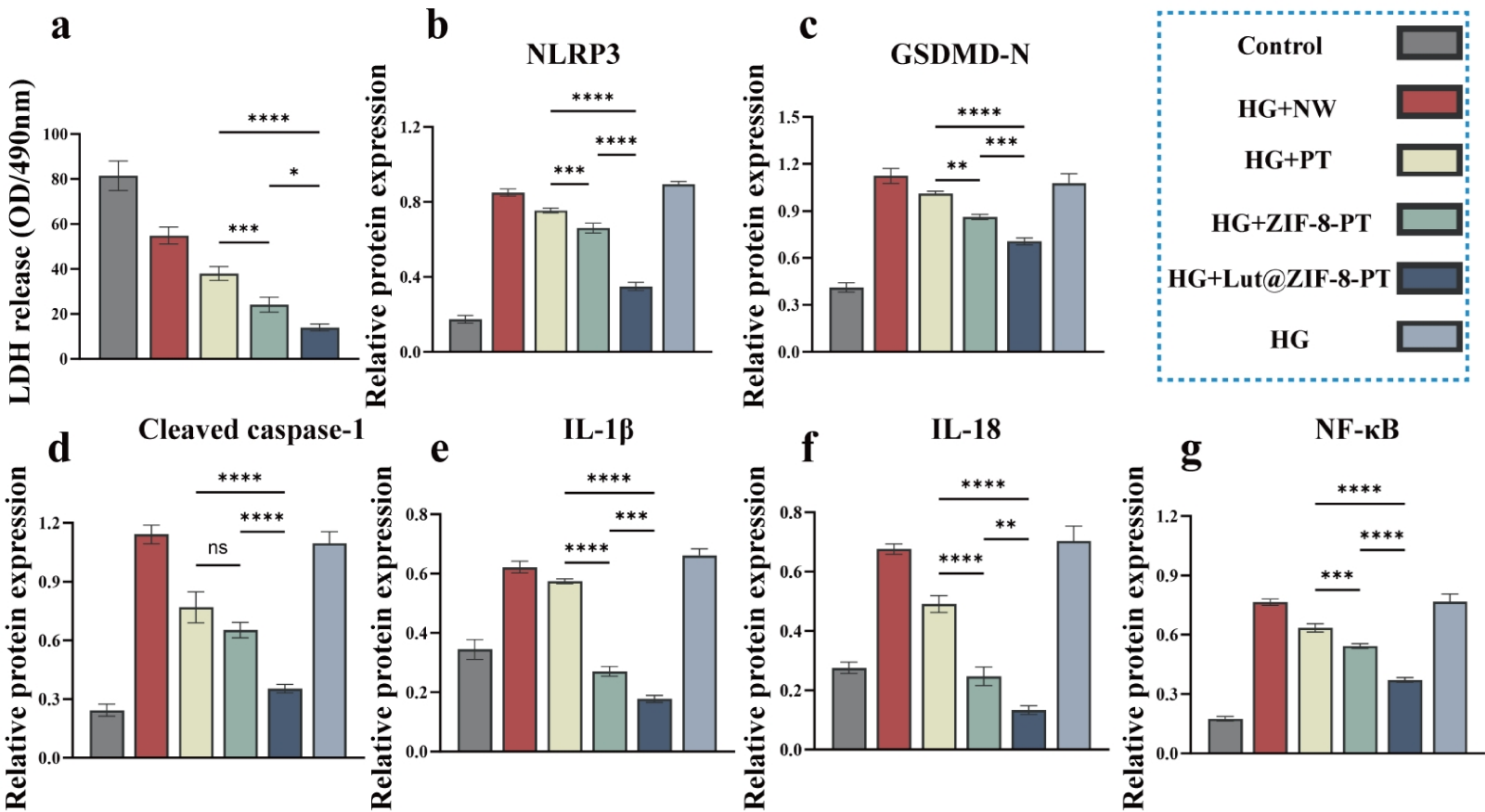

Supplement: Supplementary_Figure_15_tkag005 [file supplementary_figure_15_tkag005.pdf]

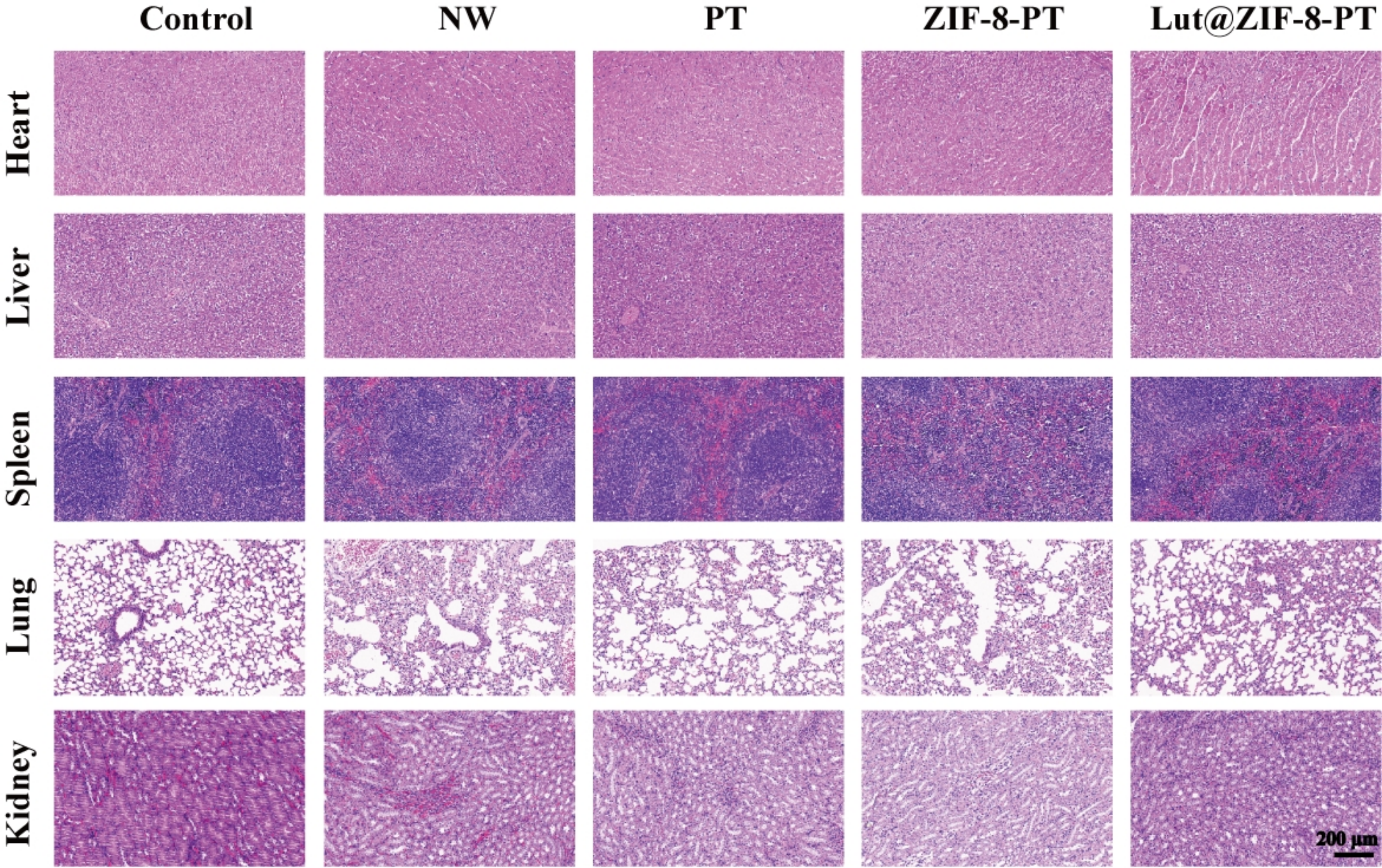

Supplement: Supplementary_Figure_16_tkag005 [file supplementary_figure_16_tkag005.pdf]
