# Supplementary material for: Multifunctional nanofibrous membranes enhance diabetic wound healing by inhibiting endothelial pyroptosis and regulating macrophage polarization
Source: Burns Trauma. 2026 Jan 19;14:tkag005. doi: 10.1093/burnst/tkag005 (PMC13011808; doi:10.1093/burnst/tkag005)
Supplement: Supplementary_Figure_14_tkag005 [file supplementary_figure_14_tkag005.pdf]

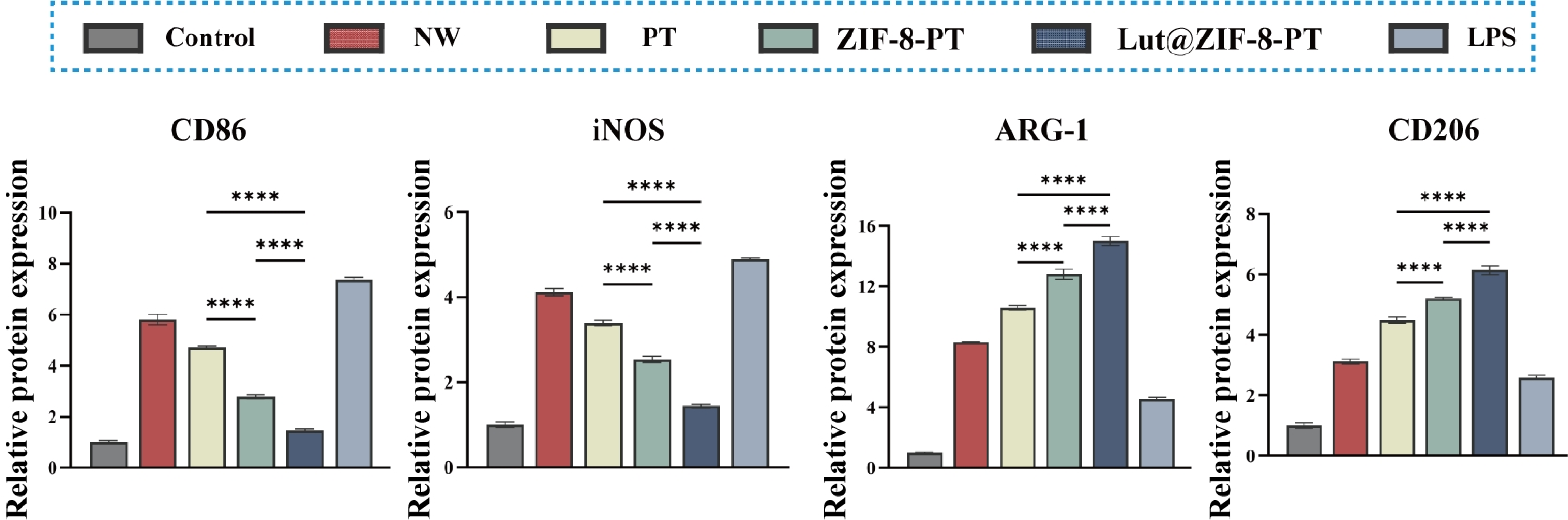
Control
NW
PT
ZIF-8-PT
Lut@ZIF-8-PT
LPS
CD86
iNOS
ARG-1
CD206
Relative protein expression
****
