# Supplementary material for: Multifunctional nanofibrous membranes enhance diabetic wound healing by inhibiting endothelial pyroptosis and regulating macrophage polarization
Source: Burns Trauma. 2026 Jan 19;14:tkag005. doi: 10.1093/burnst/tkag005 (PMC13011808; doi:10.1093/burnst/tkag005)
Supplement: Supplementary_table_1_tkag005 [file supplementary_table_1_tkag005.pdf]

Table 1. Effects of solvent type on drug loading and encapsulation efficiency.

Solvent type	Drug loading (mg/mg)	Encapsulation efficiency (%)
MeOH	0.63	34.28%
MeOH : H_2O=4:1(v/v)	0.98	53.32%
MeOH : H_2O=3:1(v/v)	1.27	69.10%
MeOH : H_2O=2:1(v/v)	1.06	57.67%
MeOH : H_2O=1:1(v/v)	0.75	40.80%
