# Supplementary material for: Multifunctional nanofibrous membranes enhance diabetic wound healing by inhibiting endothelial pyroptosis and regulating macrophage polarization
Source: Burns Trauma. 2026 Jan 19;14:tkag005. doi: 10.1093/burnst/tkag005 (PMC13011808; doi:10.1093/burnst/tkag005)
Supplement: Supplementary_table_2_tkag005 [file supplementary_table_2_tkag005.pdf]

Table 2. Effects of Lut/ZIF-8 mass ratio on drug loading and encapsulation efficiency.

| Luteolin/ZIF-8 mass ratio | Drug loading (mg/mg) | Encapsulation efficiency (%) |
|---|---|---|
| 4:1 | 1.58 | 39.50% |
| 3:1 | 1.27 | 42.33% |
| 2:1 | 1.13 | 56.50% |
| 1:1 | 0.78 | 78.00% |
