# Supplementary material for: Multifunctional nanofibrous membranes enhance diabetic wound healing by inhibiting endothelial pyroptosis and regulating macrophage polarization
Source: Burns Trauma. 2026 Jan 19;14:tkag005. doi: 10.1093/burnst/tkag005 (PMC13011808; doi:10.1093/burnst/tkag005)
Supplement: Supplementary_table_3_tkag005 [file supplementary_table_3_tkag005.pdf]

Table 3. The structure parameters of ZIF-8 and Lut@ZIF-8.

	S_{BET} (m^2/g)	D_p (Å)	V_p (cm^3/g)
ZIF-8	1152.48	40.43	0.74
Lut@ZIF-8	407.89	25.87	0.41
