# Supplementary material for: Multifunctional nanofibrous membranes enhance diabetic wound healing by inhibiting endothelial pyroptosis and regulating macrophage polarization
Source: Burns Trauma. 2026 Jan 19;14:tkag005. doi: 10.1093/burnst/tkag005 (PMC13011808; doi:10.1093/burnst/tkag005)
Supplement: SUPPORTING_INFOMATION_tkag005 [file supporting_infomation_tkag005.docx]

**SUPPORTING INFOMATION**

Supplementary Figure 1. Size distribution profiles of ZIF-8 and Lut@ZIF-8.

Table 1. Effects of solvent type on drug loading and encapsulation efficiency.

| Solvent type | Drug loading (mg/mg) | Encapsulation efficiency (%) |
| --- | --- | --- |
| MeOH | 0.63 | 34.28% |
| MeOH : H_2_O=4:1(v/v) | 0.98 | 53.32% |
| MeOH : H_2_O=3:1(v/v) | 1.27 | 69.10% |
| MeOH : H_2_O=2:1(v/v) | 1.06 | 57.67% |
| MeOH : H_2_O=1:1(v/v) | 0.75 | 40.80% |

Supplementary table 1. To evaluate the effects of solvent ratio changes and the mass ratio of Lut/ZIF-8 on drug loading and encapsulation efficiency.

Table 2. Effects of Lut/ZIF-8 mass ratio on drug loading and encapsulation efficiency.

| Luteolin/ZIF-8 mass ratio | Drug loading (mg/mg) | Encapsulation efficiency (%) |
| --- | --- | --- |
| 4:1 | 1.58 | 39.50% |
| 3:1 | 1.27 | 42.33% |
| 2:1 | 1.13 | 56.50% |
| 1:1 | 0.78 | 78.00% |

Supplementary table 2. To evaluate the effects of solvent ratio changes and the mass ratio of Lut/ZIF-8 on drug loading and encapsulation efficiency. Encapsulation efficiency (%) = (Drug loading*ZIF-8 _mass_)/Initial Luteolin _mass_*100%

Table 3. The structure parameters of ZIF-8 and Lut@ZIF-8.

|  | S_BET_ (m^2^/g) | D_p_ (Å) | V_p_ (cm^3^/g) |
| --- | --- | --- | --- |
| ZIF-8 | 1152.48 | 40.43 | 0.74 |
| Lut@ZIF-8 | 407.89 | 25.87 | 0.41 |

Supplementary table 3. The corresponding average pore diameters were 40.43 Å and 25.84 Å, and the total pore volumes were 0.74 cm^3^/g and 0.41 cm^3^/g. These results collectively confirm the successful encapsulation of luteolin within the ZIF-8 framework.


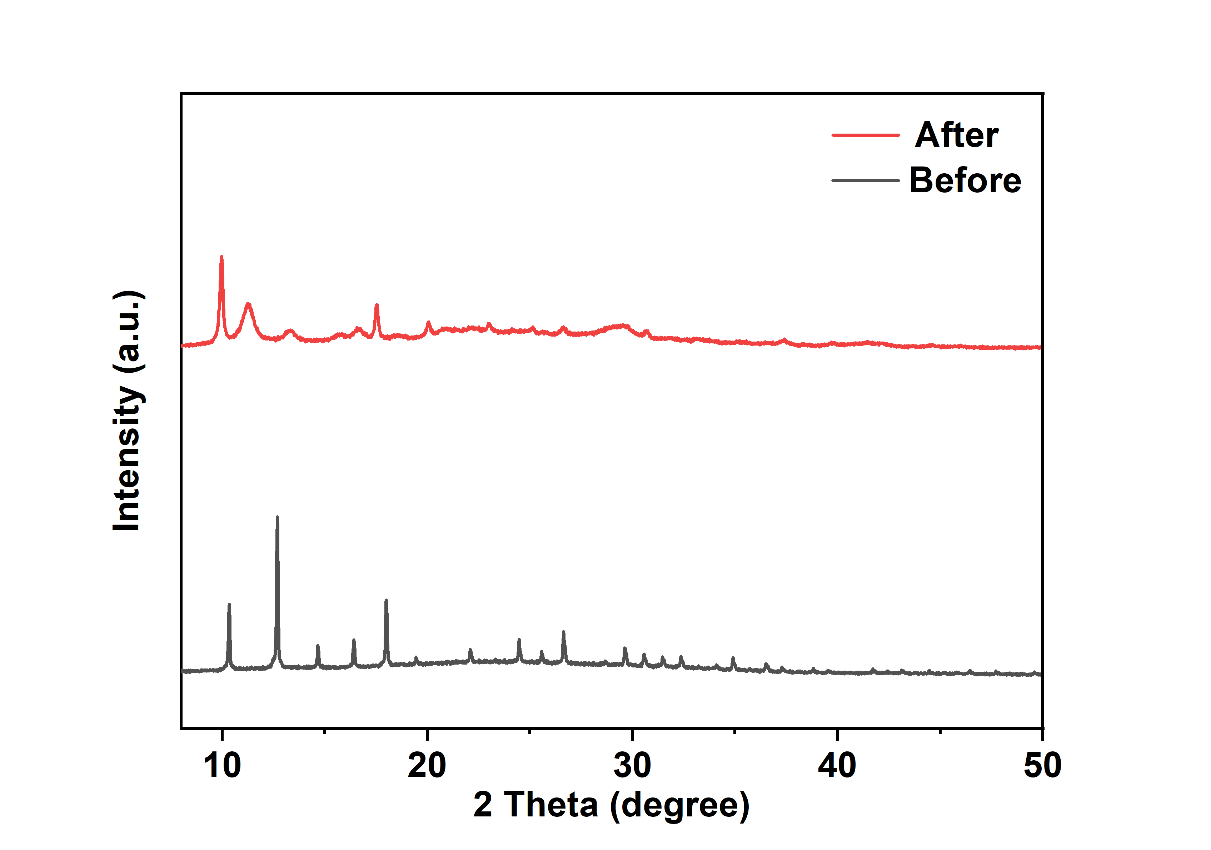


Supplementary Figure 2. XRD patterns of ZIF-8 before and after immersed in Span 80.


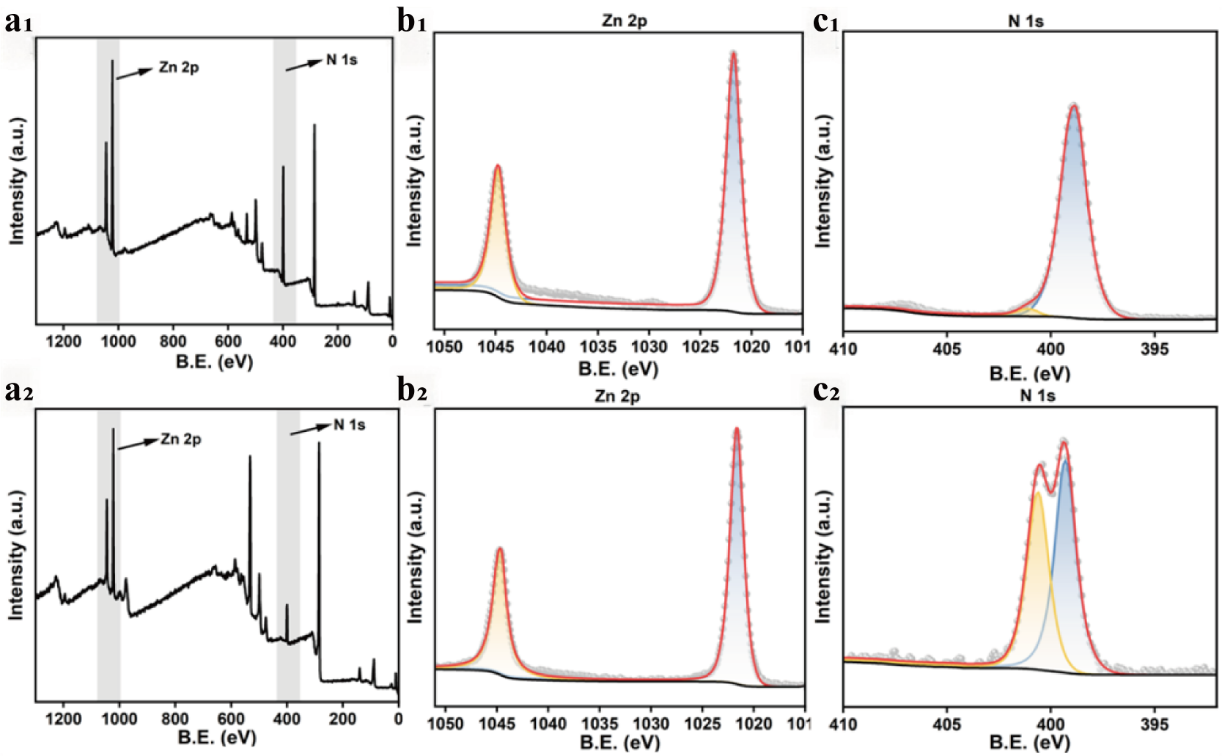


Supplementary Figure 3. High-resolution XPS spectrum of ZIF-8 (a_1_-c_1_) and Lut@ZIF-8 (a_2_-c_2_).


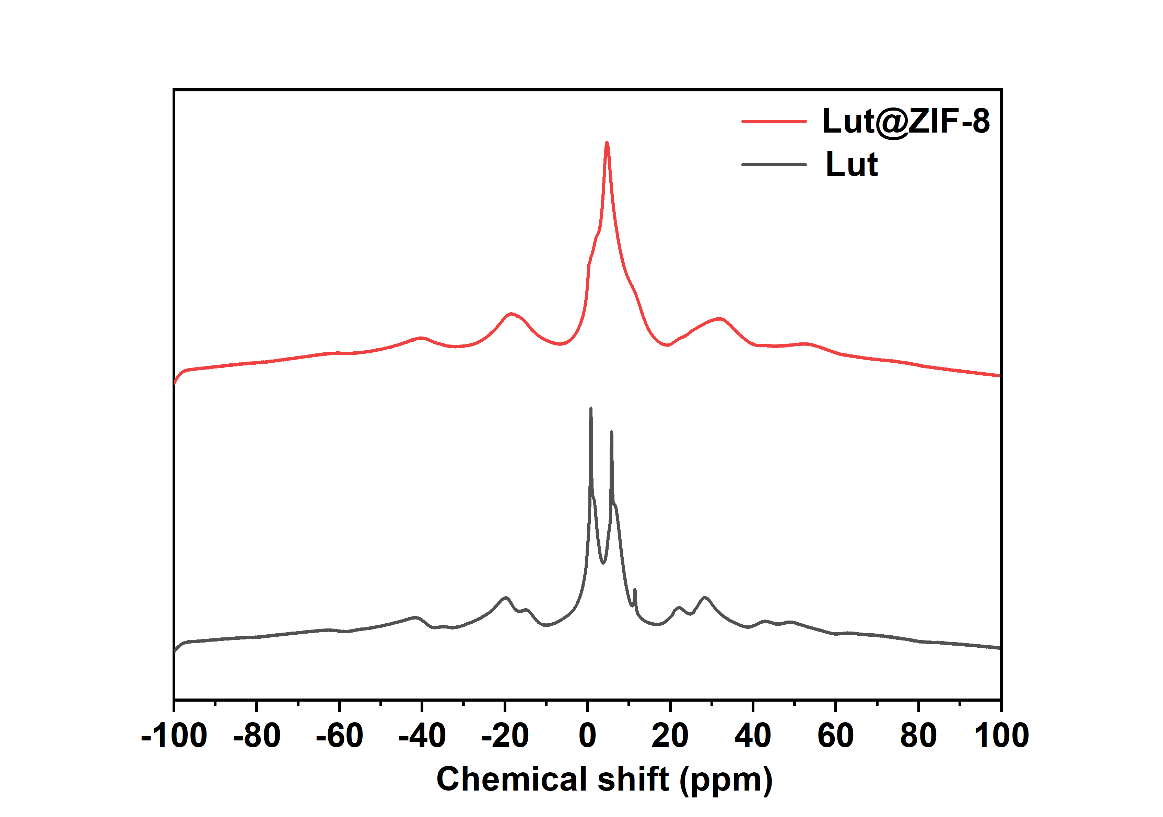


Supplementary Figure 4. Solid-state ^13^C NMR of Lut and Lut@ZIF-8.


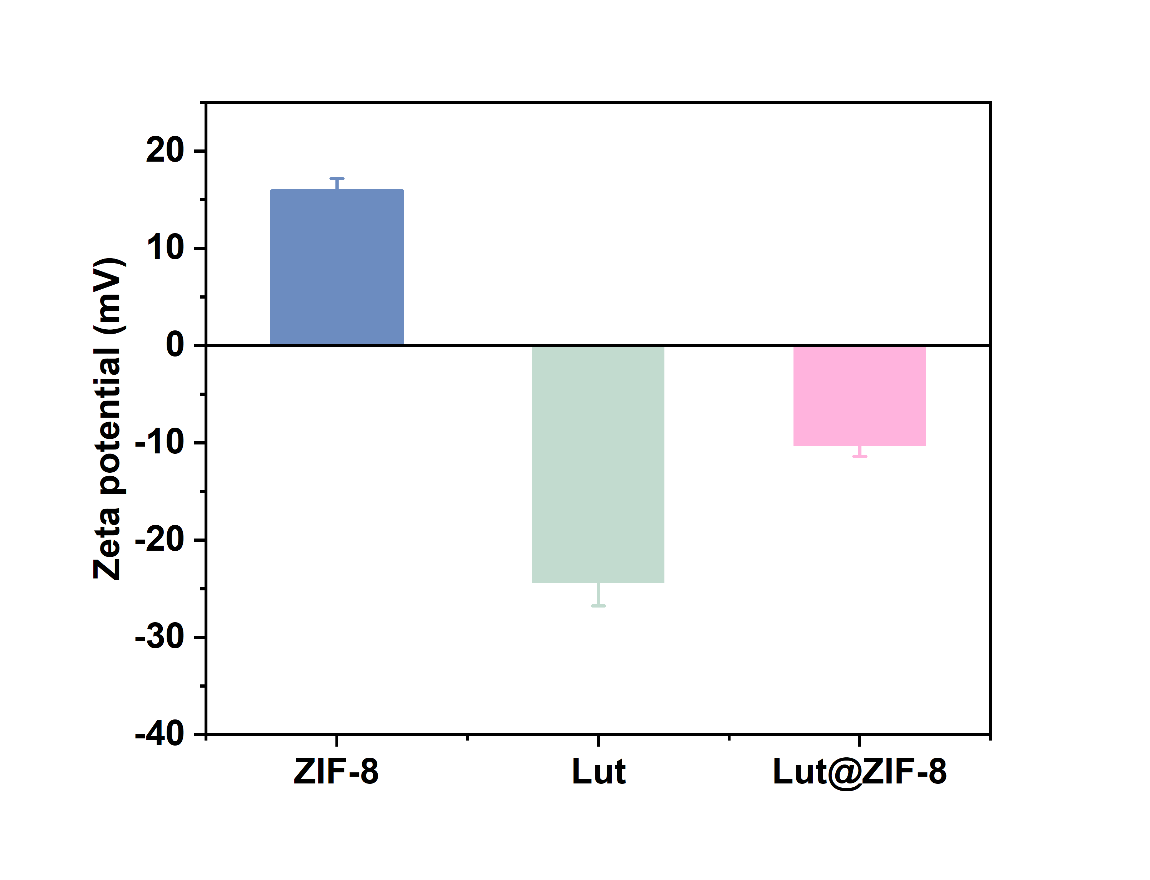


Supplementary Figure 5. Zeta potential measurements.


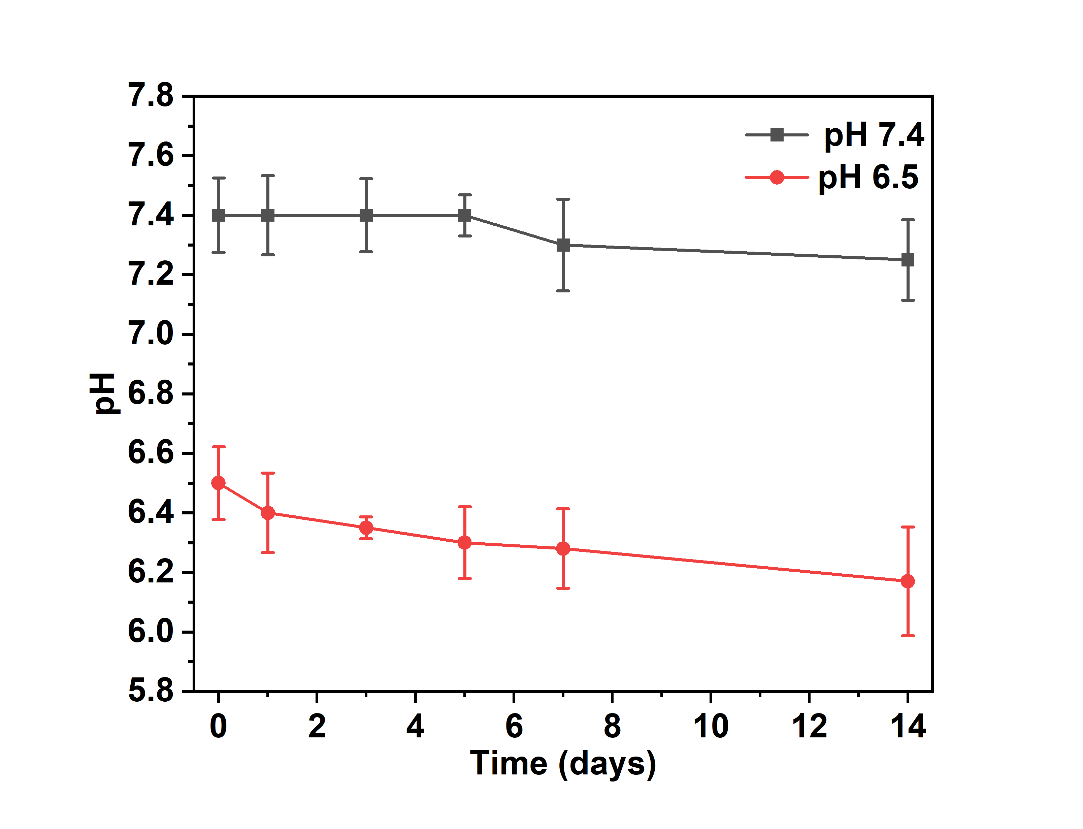


Supplementary Figure 6. The pH measurement during degradation.


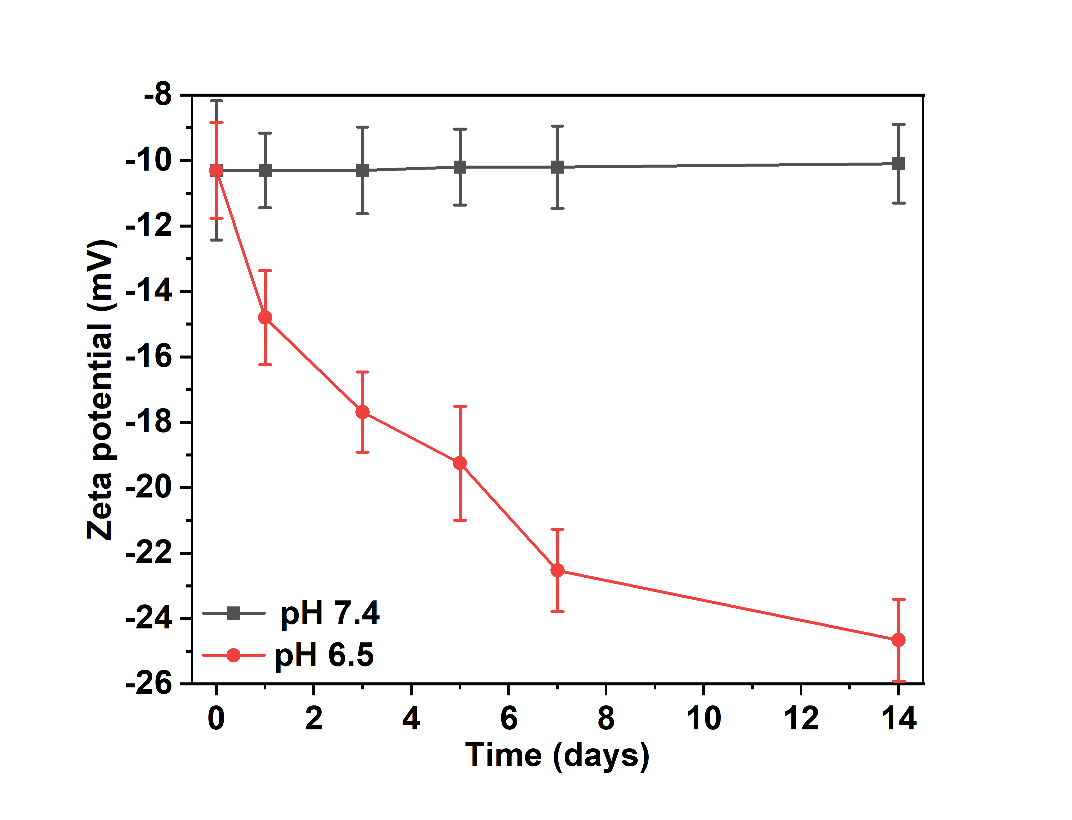


Supplementary Figure 7. The pH measurement during degradation.


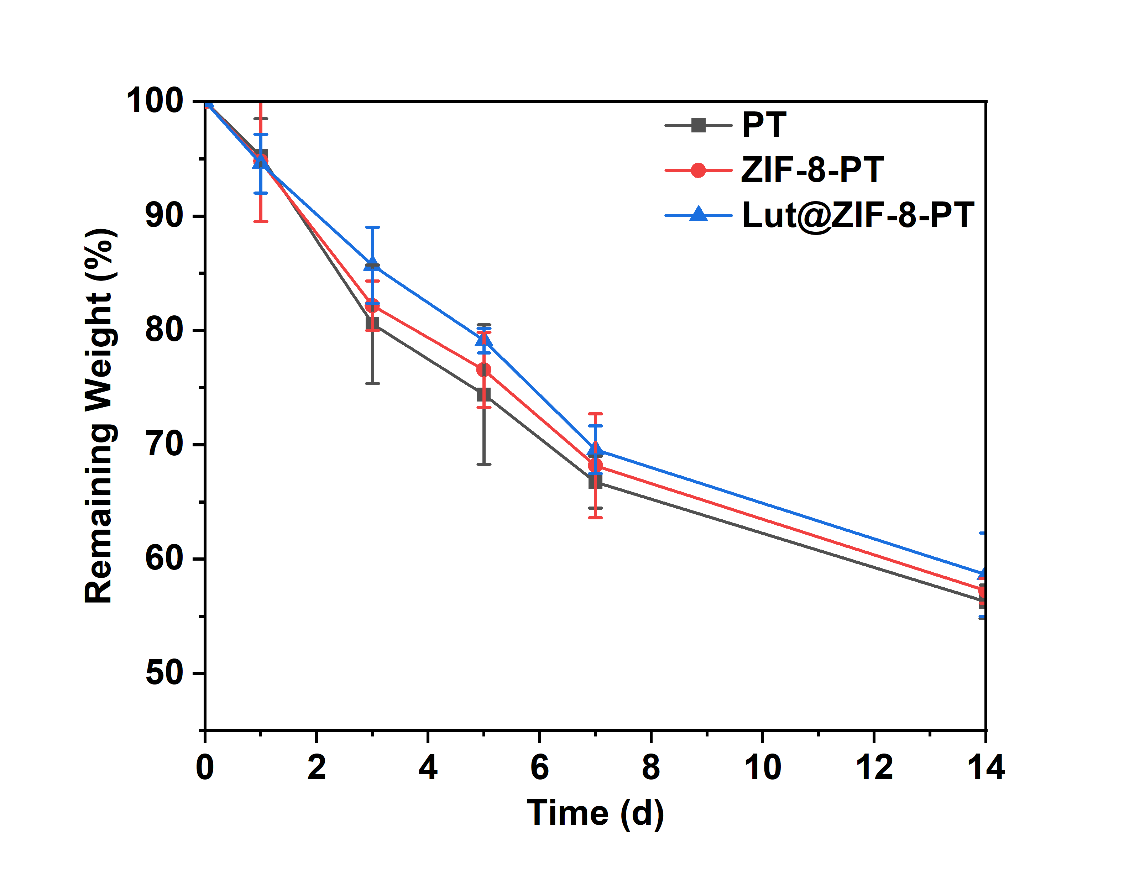


Supplementary Figure 8. Degradation rate of the PT, ZIF-8-PT and Lut@ZIF-8-PT.


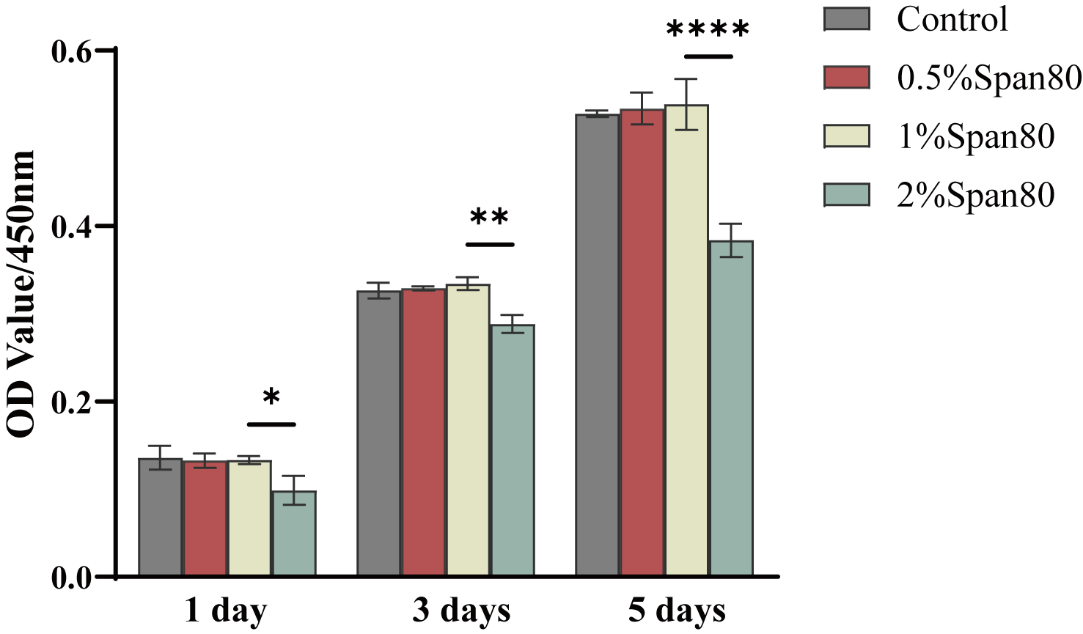


Supplementary Figure 9. Cell viability of HUVEC cells cultured on different concentration of Span 80 evaluated by CCK assay at days 1, 3, and 5.


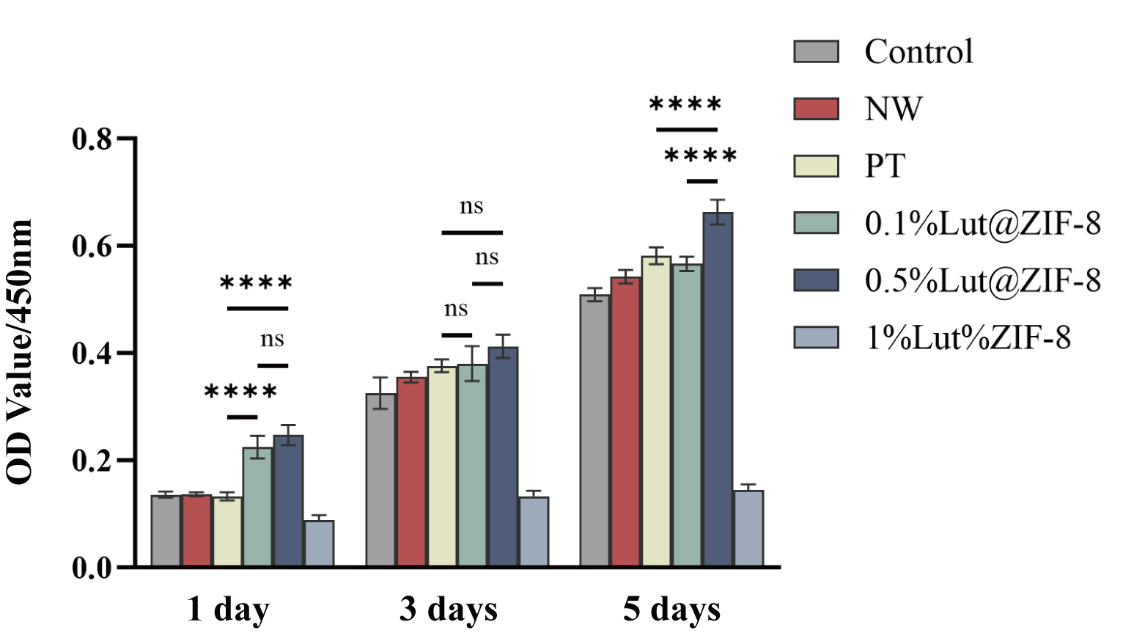


Supplementary Figure 10. Determination of optimal loading concentration of Lut@ZIF-8 by evaluating cell metabolic activity. Schematic diagram of experimental groups: Control, NW, PT, and Lut@ZIF-8-PT at 0.1%, 0.5%, and 1% loading concentrations. HUVECs Cells were incubated for 1, 3, and 5 days, and metabolic activity was measured by CCK-8 assay. Data are presented as mean ± SD (n = 3 independent experiments). Data are shown as mean ± SD. **P* < 0.05, ***P* < 0.01, ****P* < 0.001, *****P* < 0.0001.


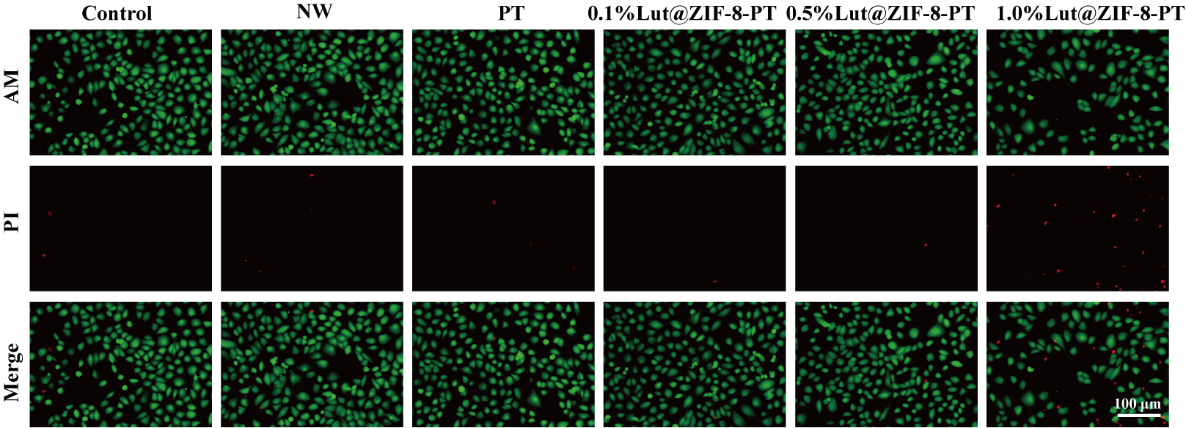


Supplementary Figure 11. Live/dead staining of HUVECs cultured on NW, PT, Lut@ZIF-8-PT at 0.1%, 0.5%, and 1% loading concentrations.

**
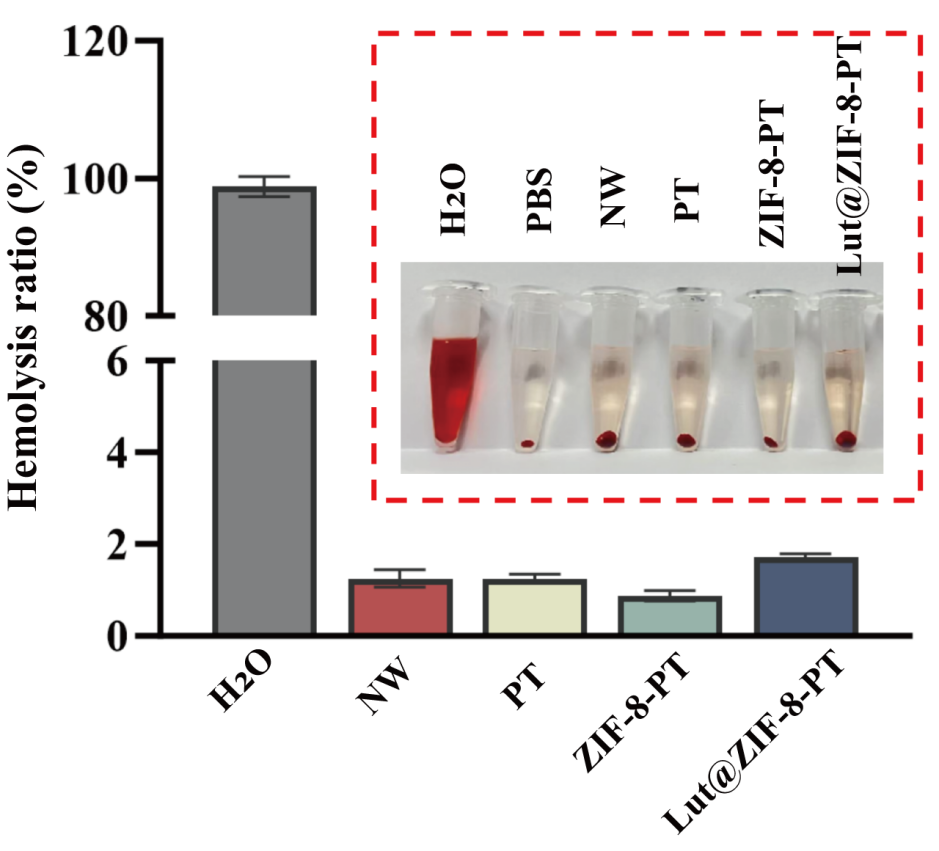
**

Supplementary Figure 12. Hemocompatibility evaluation of materials via in vitro hemolysis assay.​


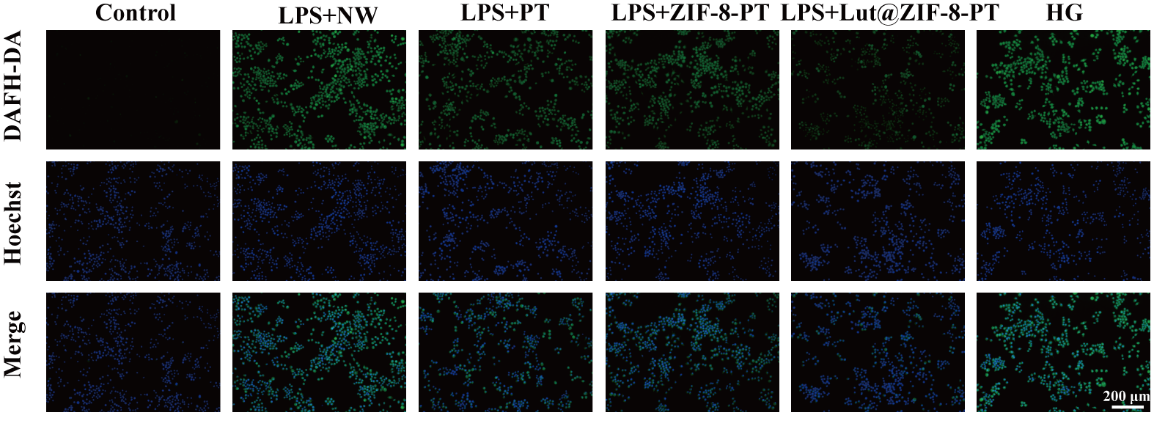


Supplementary Figure 13. Representative fluorescence microscopy images demonstrating intracellular ROS levels in treated RAW264.7 cells. Control: Control group; LPS: LPS treated group(200 ng/ml); LPS+NW: LPS treated+NW; LPS+PT: LPS treated+PT; LPS+ZIF-8-PT: LPS treated+ZIF-8-PT; LPS+Lut@ZIF-8-PT: LPS treated+Lut@ZIF-8-PT.


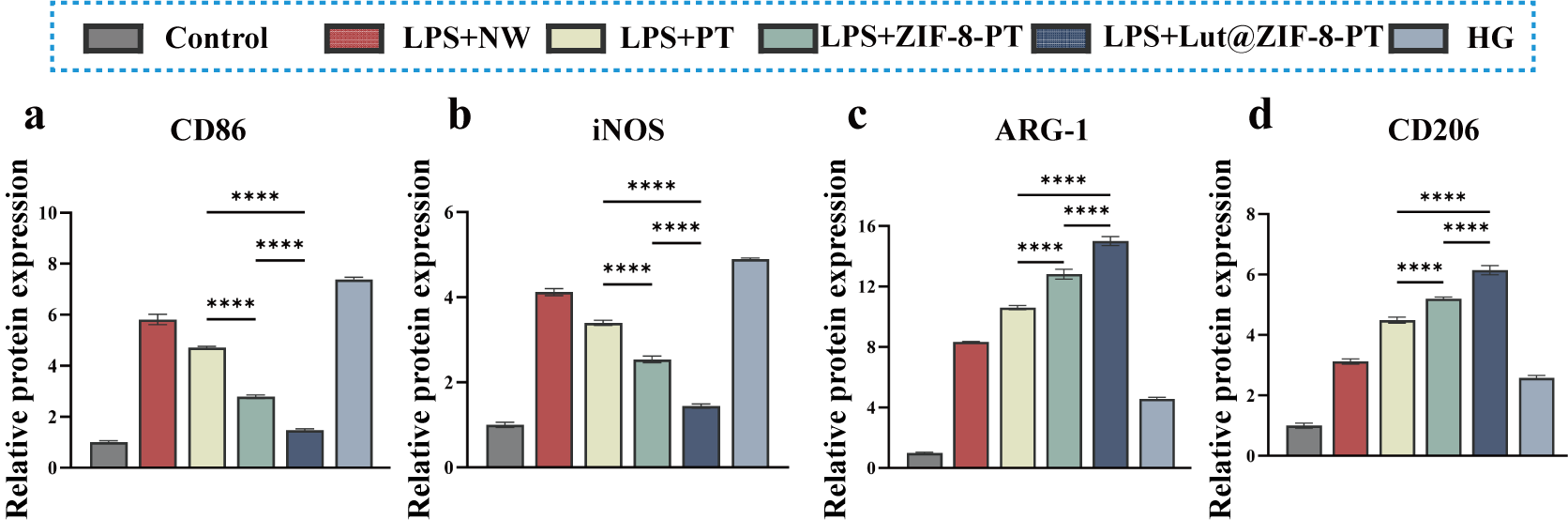


Supplementary Figure 14. Densitometric analysis of protein bands from western blot in figure 4. Control: Control group; LPS: LPS treated group(200 ng/ml); LPS+NW: LPS treated+NW; LPS+PT: LPS treated+PT; LPS+ZIF-8-PT: LPS treated+ZIF-8-PT; LPS+Lut@ZIF-8-PT: LPS treated+Lut@ZIF-8-PT, Data are shown as mean ± SD. **P*< 0.05, ***P* < 0.01, ****P* < 0.001, *****P* < 0.0001.


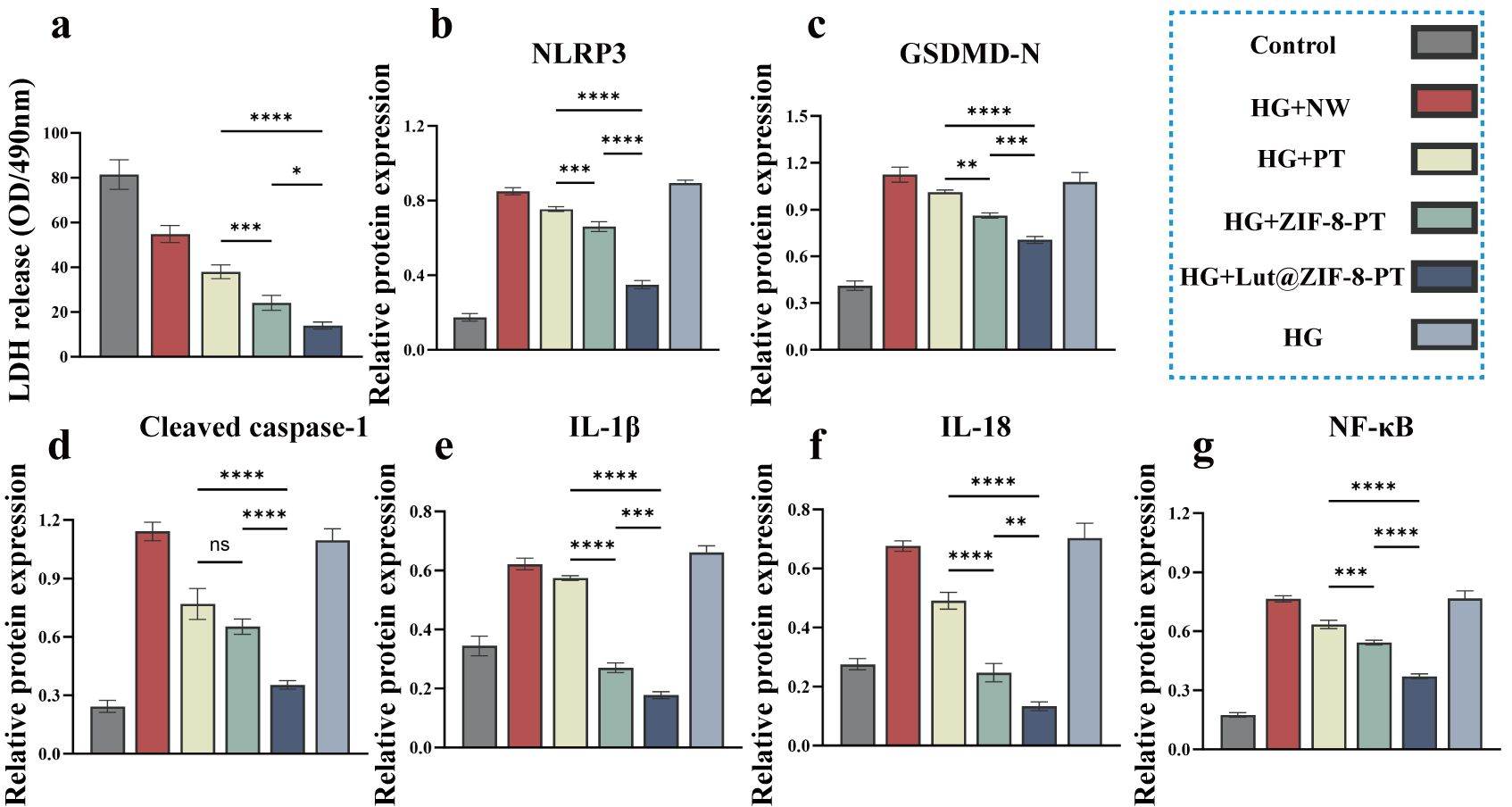


Supplementary Figure 15. LDH releasing level. Quantification of GSDMD-N, GSDMD, C-Caspase-1, NLRP3, IL-1β, IL-18, and NF-κB protein expression in Figure 6. Control: Control group; HG: High-glucose model group(30 mM); HG+NW: High-glucose+NW; HG+PT: High-glucose+PT; HG+ZIF-8-PT: High-glucose +ZIF-8-PT; HG+Lut@ZIF-8-PT：High-glucose+Lut@ZIF-8-PT, Data are shown as mean ± SD. **P* < 0.05, ***P* < 0.01, ****P* < 0.001, *****P* < 0.0001.


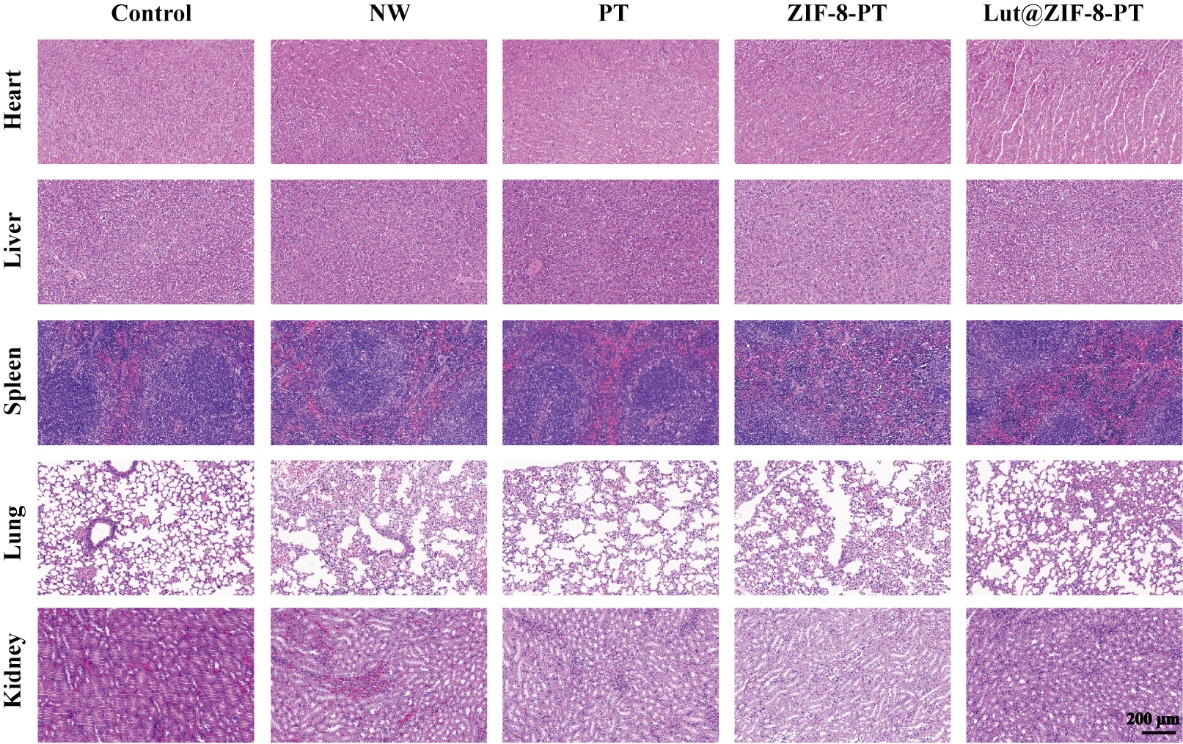


Supplementary Figure 16. H&E staining images of major organs from the five groups. Control: STZ group; NW: STZ+NW group; PT: STZ+PT group; ZIF-8-PT: STZ +ZIF-8-PT group; Lut@ZIF-8-PT: STZ+Lut@ZIF-8-PT group,
